# Indications for tonsillectomy stratified by the level of evidence

**DOI:** 10.3205/cto000136

**Published:** 2016-12-15

**Authors:** Jochen P. Windfuhr

**Affiliations:** 1 Department of Otolaryngology, Head & Neck Surgery, Allergology, Kliniken Maria Hilf, Mönchengladbach, Germany

**Keywords:** tonsillectomy, tonsillotomy, tonsillitis, sleep-related breathing disorders, IgA nephropathy, psoriasis, peritonsillar abscess, complications, post-tonsillectomy hemorrhage

## Abstract

**Background:** One of the most significant clinical trials, demonstrating the efficacy of tonsillectomy (TE) for recurrent throat infection in severely affected children, was published in 1984. This systematic review was undertaken to compile various indications for TE as suggested in the literature after 1984 and to stratify the papers according to the current concept of evidence-based medicine.

**Material and methods:** A systematic Medline research was performed using the key word of “tonsillectomy“ in combination with different filters such as “systematic reviews“, “meta-analysis“, “English“, “German“, and “from 1984/01/01 to 2015/05/31“. Further research was performed in the Cochrane Database of Systematic Reviews, National Guideline Clearinghouse, Guidelines International Network and BMJ Clinical Evidence using the same key word. Finally, data from the “Trip Database” were researched for “tonsillectomy” and “indication“ and “from: 1984 to: 2015“ in combination with either “systematic review“ or “meta-analysis“ or “metaanalysis”.

**Results:** A total of 237 papers were retrieved but only 57 matched our inclusion criteria covering the following topics: peritonsillar abscess (3), guidelines (5), otitis media with effusion (5), psoriasis (3), PFAPA syndrome (6), evidence-based indications (5), renal diseases (7), sleep-related breathing disorders (11), and tonsillitis/pharyngitis (12), respectively.

**Conclusions:** 1) The literature suggests, that TE is not indicated to treat otitis media with effusion. 2) It has been shown, that the PFAPA syndrome is self-limiting and responds well to steroid administration, at least in a considerable amount of children. The indication for TE therefore appears to be imbalanced but further research is required to clarify the value of surgery. 3) Abscesstonsillectomy as a routine is not justified and indicated only for cases not responding to other measures of treatment, evident complications, or with a significant history of tonsillitis. In particular, interval-tonsillectomy is not justified as a routine. 4) TE, with or without adenoidectomy, is efficacious to resolve sleep-related breathing disorders resulting from (adeno)tonsillar hypertrophy in children. However, the benefit is reduced by co-morbidities, such as obesity, and further research is required to identify prognostic factors for this subgroup of patients. Further research is indicated to clarify selection criteria not only for this subpopulation that may benefit from less invasive procedures such as tonsillotomy in the long-term. 5) Further trials are also indicated to evaluate the efficacy of TE on the clinical course in children with psoriasis guttata as well as on psoriasis vulgaris in adults, not responding to first-line therapy. 6) Conflicting results were reported concerning the role of TE in the concert to treat Ig-A nephropathy, mandating further clinical research. 7) Most importantly, randomized-controlled clinical trials with an adequate long-term follow-up are desirable to clarify the benefit of TE in patients with recurrent episodes of tonsillitis, with or without pharyngitis. Factors like age, spontaneous healing rate and postoperative quality of life have to be included when comparing TE with antibiotic therapy.

## 1 Introduction

“A convincing demonstration of the absurdity of indiscriminate tonsillectomy was given ten years ago by the American Child Health Association.” This citation originates from an article written by Harry Bakwin in 1945 entitled “Pseudoxia pediatrica” [1]. The author refers to a report of 1,000 schoolchildren in New York, 61% of them had already undergone tonsillectomy (TE) at the age of about 11 years. Three examination procedures performed by school doctors revealed that only 65 children had not had such an intervention. This example shows very clearly how the former developments in medicine together with seemingly logic conceptions of a so-called focus theory led to a completely undifferentiated indication of TE as routine surgery [2], [3]. The term “routine” is found literally as justifying indication of surgery as well as cough, stomach ache with fever, arthritis, pyelitis, uveitis, debility, and bronchopneumonia [4]. Meanwhile we are far from these indiscriminate indications, but TE is still acknowledged as a common, minor procedure by the general population. Among the most common 20 diagnoses in children, *chronic diseases of the palatal and the pharyngeal tonsils* were the second most affecting 54,790 pediatric patients (15 years and younger) in 2013 in German hospitals. The same ranking mentions acute tonsillitis as number 19 with 11,066 cases [5]. Doctors in practical training learn this surgical technique at a very early stage of training and perform tonsillectomies mostly during their whole professional life, more or less frequently. The broad distribution and high frequency of surgery in single hospitals can be found in their structured quality reports [6], [7]. Especially due to the high number of interventions and the seemingly low difficulty, the intervention is assumed to be a trivial matter not only by non-medicals but also by professionals, which sometimes leads to a complete misjudgment of the risk of postoperative bleeding and to misconduct [8].

### 1.1 Opinion-based vs. evidence-based medicine

Regarding the question of indication, the treating physician is in a crucial situation since he has to synthesize therapy recommendations in the individual case based on theoretical knowledge and own experience (internal evidence) and the flood of literature and studies (external evidence). Besides the problem of time to deal with the permanently renewing literature, there is often also a discrepancy between the internal and the external evidence (opinion-base vs. evidence-base). This is the reason why evidence-based medicine is often perceived as a limitation of the medical freedom of opinion, also because the health insurances use evidence-based medicine as an argument to justify retention of payments. The Medical Service of the National Association of Statutory Health Insurance Funds (Medizinischer Dienst des Spitzenverbandes Bund der Krankenkassen) states: “By applying evidence-based medicine, underuse, overuse, or misuse of medical care will be avoided or remedied if such an inappropriate treatment has already occurred. The application of evidence-based medicine does not automatically mean that the patient has to undergo even more diagnostic or therapeutic procedures – in contrary, evidence-based medicine shall also lead to avoiding useless or even harmful applications or to stop such procedures” [9]. The situation of the treating physicians is intensified by the expectations of the patients/parents to be treated rapidly and without any failure which seems to be assured by evidence-based medicine. In addition, there is the half-knowledge gained through internet research by patients/parents that leads to an opinion already before medical consultation. A good example in this context is the assumption often expressed by non-medicals that TE causes damage to the immune system, which has never been proven scientifically [10], [11], [12]. Even discussions regarding the significance of the patients’ age for indication of TE [13], [14], [15] or tonsillotomy (TOTO) [16] are not supported by scientific data.

**Figure 1:**
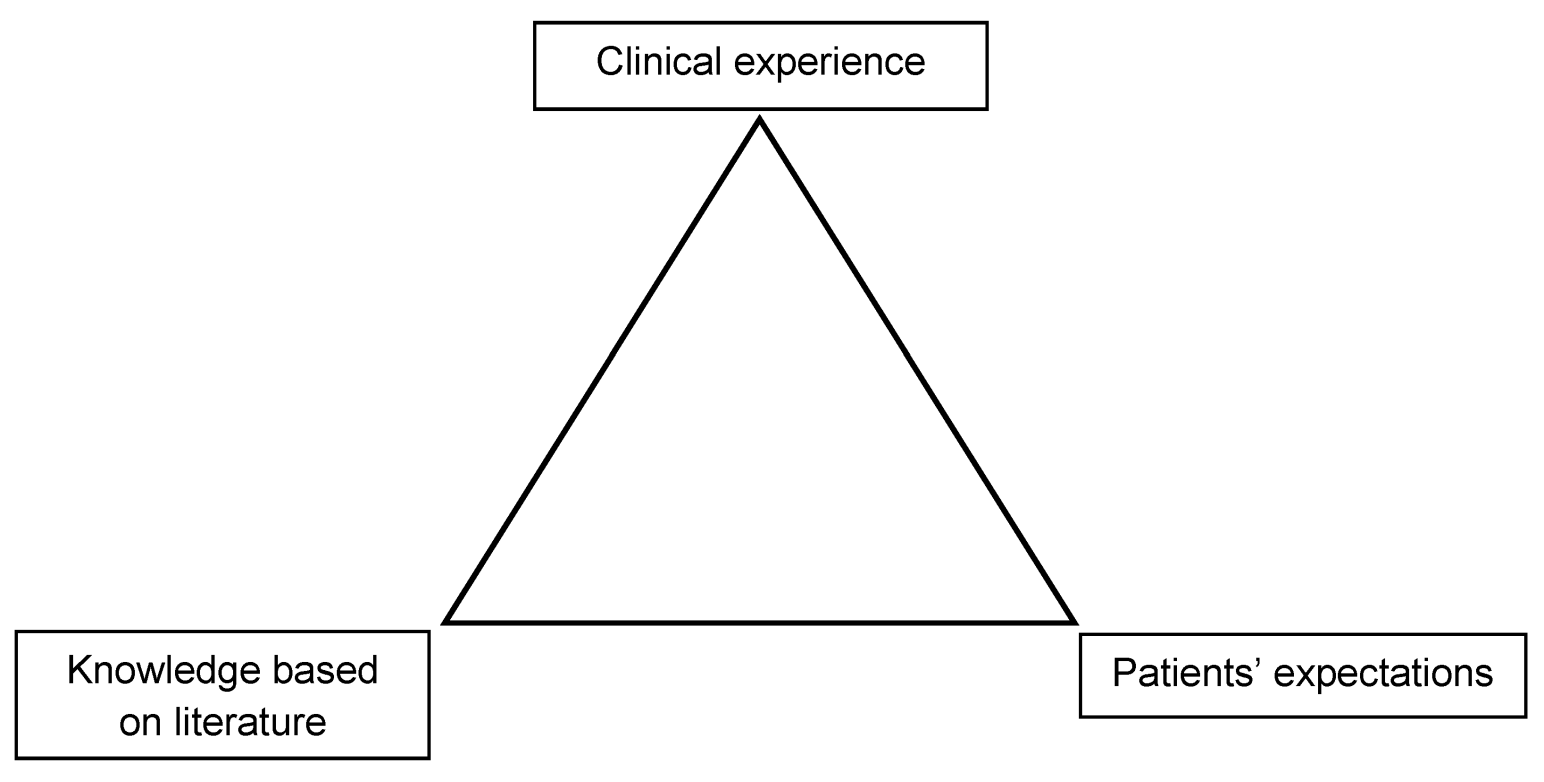
Conflict situation of medical consultation

Finally, it is a dilemma that a high percentage of different health care services is not confirmed by valid scientific data. However, the inverse conclusions does not logically mean that the majority of interventions is not useful (“absence of evidence is not evidence of absence”) (Figure 1 (Fig. 1)).

### 1.2 Evidence-based medicine

In Germany, different institutions apply evidence-based medicine to assess surgical and diagnostic procedures, pharmaceutical products, treatment guidelines, and disease management programs (DMP). This occurs either on a scientific level (German Network of Evidence-Based Medicine, Deutsches Netzwerk Evidenzbasierte Medizin DNEbM e.V.) or on a political stage as for example the *Institut für Qualität und Wirtschaftlichkeit* (*IQWiG, Institute for Quality and Cost Effectiveness*), the *Gemeinsamer Bundesausschuss* (*G-BA, Federal Joint Committee*), the Federal Ministry of Health, or the *Ärztliches Zentrum für Qualität in der Medizin* (*Medical Center for Quality in Medicine*) [17].

A very ambitious analysis and evaluation of the scientific literature in the sense of evidence-based medicine is performed by the Cochrane Collaboration founded in 1993, named after the British physician and epidemiologist Sir Archibald Leman Cochrane (1909–1988). His book entitled “Effectiveness and Efficiency. Random reflections on health services” (1972) was the basis for the foundation of the Cochrane Collaboration and a model for the reference work on “Effective care in pregnancy and childbirth” (1989) by Iain Chalmers [18], [19] that is still regularly updated. This work is based on systematic review articles and study registers and is considered as the first evidence-based therapy manual [17]. A co-founder of the Cochrane Collaboration is David Sackett (1934–2015) who had the first professorship worldwide for clinical epidemiology and biostatistics at the McMaster University of Hamilton, Canada, in 1967 and who founded the Oxford Centre for Evidence-Based Medicine in England in 1994 [20]. Meanwhile, more than 28,000 physicians, scientists, employees of the health care system, and patient representatives in more than 120 countries work for the Cochrane Collaboration with the aim of providing current medical information and evidence for prevention, treatment, and rehabilitation of certain health problems or fields to the public and thus facilitating decisions on treatment options for all people involved, physicians, patients, and relatives. For this purpose, systematic review articles are elaborated, actualized, and distributed and listed in the Cochrane Library [21].

According to David Sackett, evidence-based medicine is “the conscientious, explicit, and judicious use of current best evidence in making decisions about the care of individual patients. The practice of evidence based medicine means integrating individual clinical expertise with the best available external clinical evidence from systematic research“ [17].

The procedure in evidence-based medicine follows 5 steps:

1. clinical problem as question for scientific investigation
2. systematic research of the literature to find appropriate trials
3. critical assessment of evidence of all identified trials
4. application of gained knowledge in consideration of the concrete clinical situation
5. self-critical evaluation and adaptation if needed of the current procedures

Evidence-based medicine does not carry out trials but primarily analyzes and assesses already existing clinical studies and their results. The assessment of study concepts is a crucial issue because the significance of the conclusions and the consequences for medical practice depend on them. The basis of trial assessment is a systematic research of the literature. In this context it is important to analyze the data situation as exhaustively and completely as possible in order to avoid mistakes already in a first step that might be caused by an incomplete and biased evaluation of data. The research, the assessment, and the interpretation of the sources should be made in a comprehensible and reproducible way in the sense of transparency [9].

### 1.3 Objective

This systematic research of the literature aims to compile and classify systematic review articles, meta-analyses, and guidelines referring to different indications of TE that were published after 1983.

## 2 Material and methods

In reference to the recommendations of the German Cochrane Center [22], this article is based on selected primary literature (here: *Medline*; Cochrane Database Clinical Trials), secondary literature (here: Clinical Evidence; Trip Database), and summaries (here: Guidelines). Similar recommendations can be found at the *Centre for Evidence-Based Medicine Oxford* (CEBM) [23]. Regarding actualizations of already published contributions of the same authors/institutions, only the most current version was considered in the research. Announced actualizations of guidelines or hints on already existing guidelines were assessed in form of a separate research contacting the respective scientific society.

### 2.1 Primary literature

The Medline research [24] was carried out with the keyword “tonsillectomy” combined with the filters of “systematic review”, “meta-analysis”, “English”, “German”, and “from 1984/01/01 to 2015/05/31”. On June 7, 2015, the keyword of “tonsillectomy” was used for the Cochrane Database Systematic Reviews for the published results of systematic literature researches including electronic database researches and manual search of controlled clinical trials [25].

### 2.2 Secondary literature

The secondary literature research included the literature research on May 31, 2015, on the database of *BMJ Clinical Evidence* [26] with the keyword “tonsillectomy” and on June 7, 2015, on the database of *Trip Database* [27] with the keywords “tonsillectomy” and “indication”, and “from: 1984 to: 2015” combined with “systematic review” or “metaanalysis” or “meta-analysis”, respectively.

### 2.3 Synthesis of evidence

This terms means guidelines in particular [28]. The keyword “tonsillectomy” was searched in the register of the *National Guideline Clearinghouse* [29] and the *Guidelines International Network* [30] on May 31, 2015. 

### 2.4 Inclusion and exclusion criteria

Systematic review articles, meta-analyses, and guidelines referring to the indication of TE in German or English language were included. Publications were excluded when the reports dealt with the following aspects:

- Surgical procedures
- Adjuvant therapies
- Routine histology
- Microbiological examinations
- Postoperative results/complications
- Analysis of risk factors for bleeding complications
- Treatment errors
- Tonsillotomy (synonymous: intracapsular/partial tonsillectomy; tonsillar ablation)
- Outpatient vs. inpatient treatment
- Single case reports
- Single prospective/retrospective mono-/multicenter trials
- Risk of surgical measures in pediatric patients
- Medico-legal questions
- Effect on postoperative laboratory parameters
- Postoperative quality of life
- Different postoperative care for children
- Risk factors for surgical failure
- Reports without abstract
- Postoperative examination procedures
- Benefit of preoperative diagnostics
- Side effects of tonsillectomy
- Actualization of reviews/guidelines by the same first author or the same group
- Duplicates
- Question-answer documents with short citations of already identified literature
- Implementation of guidelines
- Articles without references
- Treatment suggestions in cases of unilateral tonsillar augmentation
- General review articles with only cursory mentioning of tonsillectomy
- eTextbooks (Trip Database)
- Malignomas

### 2.5 Presentation of the literature

The results of this literature research was summarized in a narrative way. The identified publications were assigned to the single diseases. If a meta-analysis of the Cochrane Collaboration was found among the assigned literature, it was summarized first in detail including the trials assessed there. Afterwards, other publications on the respective topic were reported, starting with the most recent one. The presentation of the methods, results, and summaries was performed as written by the authors. The content of the national guidelines as well as of review articles on indications of surgery (“evidence-based indications”) was described in a separate chapter, if present.

### 2.6 Evaluation of studies

The evaluation of the evidence was performed within the concept of the “Oxford 2011 Levels of Evidence” [31].

- Level 1: systematic review of randomized controlled trials (RCT)
- Level 2: RCT or observational study with dramatic effect
- Level 3: non-randomized controlled cohort trial/follow-up study
- Level 4: case series or retrospective trials without control groups
- Level 5: case reports, expert opinions (Table 1 (Tab. 1))

## 3 Results

### 3.1 Research of the literature

**Figure 2:**
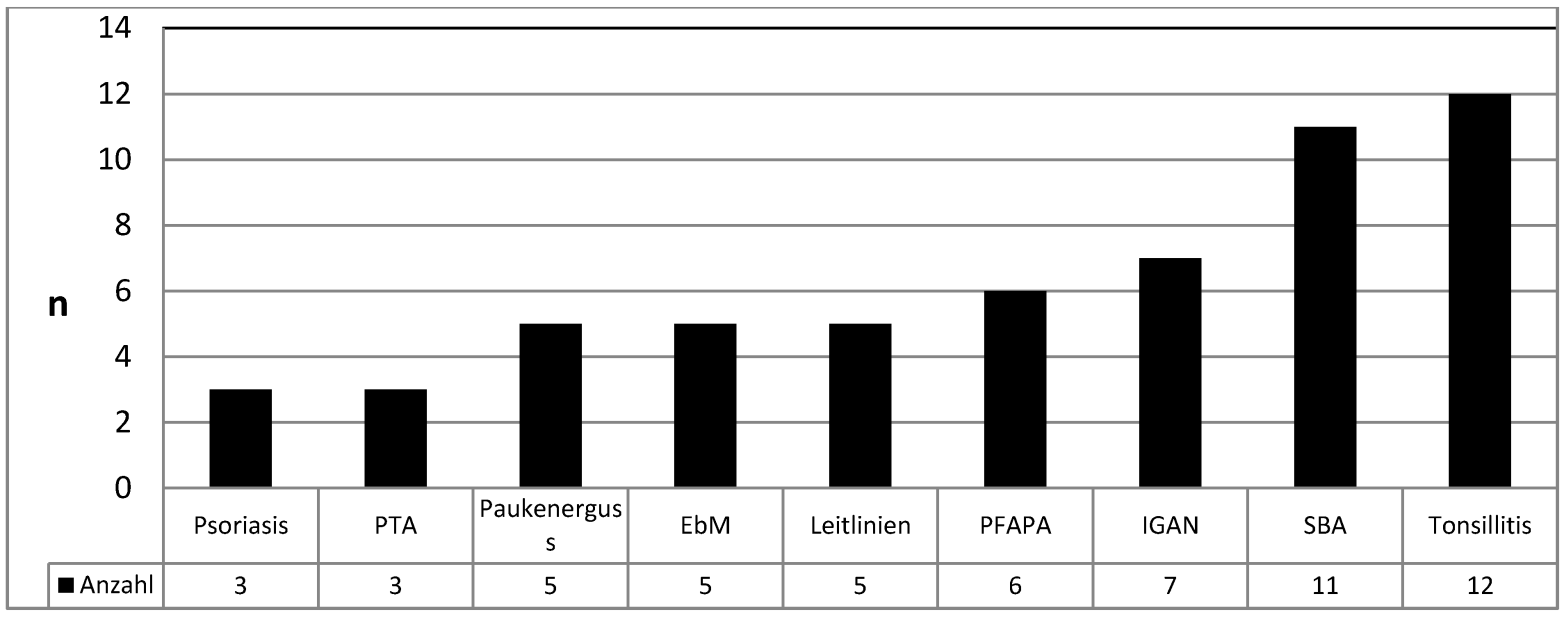
Topics of 57 publications analyzed in this systematic review PTA = peritonsillar abscess; PFAPA = periodic fever, aphthous stomatitis, pharyngitis, and adenitis syndrome; EbM = evidence-based medicine indications; IGAN = IgA nephropathy

Thirty-eight of 218 hits of the Medline research were eligible, among those, 5 were published by the Cochrane Collaboration [32], [33], [34], [35], [36] as well as 1 short version [37] of a separately included doctoral thesis (so-called grey literature) [38]. After evaluating the 23 articles listed in the *Cochrane Database of Systematic Reviews*, there was one additional article matching the inclusion criteria [39]. The research of *Trip Database* led to 249 hits, 16 of which were eligible including another guideline [40]; the research of *BMJ Clinical Evidence* did not provide any article adding further information. Only one of 12 guidelines listed in the *National Guideline Clearinghouse* had not been found with the previous research strategies [41]. The register of the *Guidelines International Network* did not reveal any guideline that had not been found by the previous research. Including the secondary literature, thus 57 publications were the basis for this review article that could be assigned to 10 different topics: peritonsillar abscess (3), psoriasis (3), guidelines (5), otitis media with effusion (5), evidence-based indications (5), PFAPA syndrome (6), renal diseases (7), sleep-related breathing disorders (11), and tonsillitis/pharyngitis (12) (Figure 2 (Fig. 2)).

**Figure 3:**
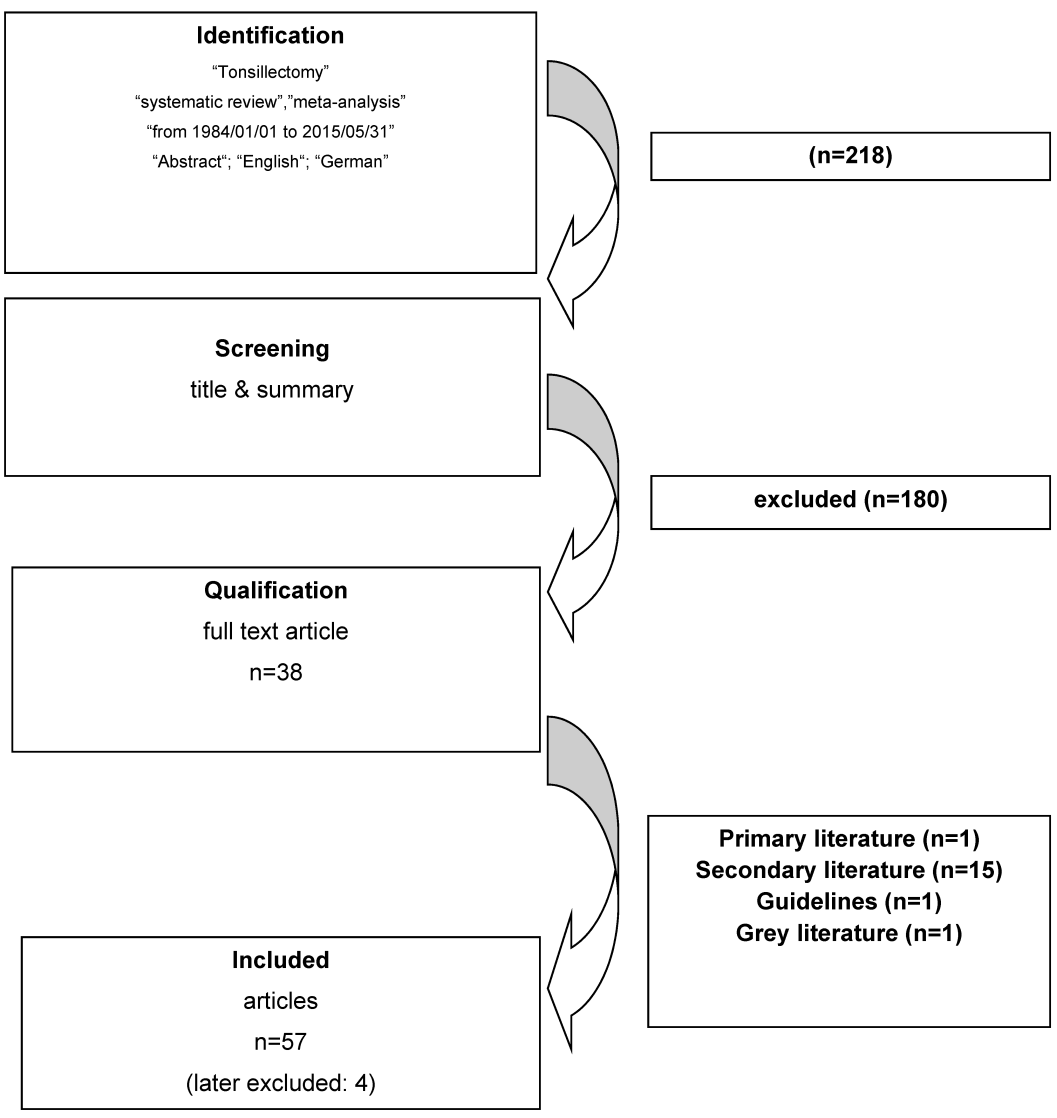
PRISMA flow diagram The Medline literature research was performed according to the validated PRISMA recommendations [42]. PRISMA = preferred reporting items for systematic reviews and meta-analyses. The diagram shows the information flow during the different phases of literature analysis. Inclusion criteria: guidelines and review articles and articles on the indication of tonsillectomy and postoperative quality of life. Exclusion criteria: eTextbooks (in Tripdatabase) as well as reports on: surgical methods, pain therapy, adjuvant therapy, routine histology, microbiological examinations, postoperative effects/complications, risk factor analysis for bleeding complications, treatment failure, tonsillotomy (syn.: intracapsular/partial tonsillectomy, tonsillar ablation), implementation of guidelines, outpatient treatment options, single case studies, single prospective/retrospective mono-/multicenter trials, general risks of surgical measures in pediatric patients, medico-legal questions, effect on postoperative laboratory parameters, quality of life after different ENT specific interventions, different postoperative types of care for children, risk factors for surgical failure, articles without abstracts, postoperative examination procedures, benefit of preoperative diagnostics, side effects of tonsillectomy, publications not in German or English language, actualizations of previously published review articles by the same first author, implementation of guidelines, articles without references, or treatment suggestions in cases of unilaterally increased tonsils.

The Medline research was performed according to the validated PRISMA recommendations (PRISMA = **p**referred **r**eporting **i**tems for **s**ystematic reviews and **m**eta-**a**nalyses) [42]. The diagram (Figure 3 (Fig. 3)) shows the information flow during the different phases of the literature analysis. Inclusion criteria: guidelines as well as review articles on indication of TE and postoperative quality of life. Exclusion criteria: eTextbooks (Trip Database) as well as reports on: surgical methods, pain therapy, adjuvant therapies, routine histology, microbiological examinations, postoperative effects/complications, analysis of risk factors for bleeding complications, treatment errors, TOTO (syn.: intracapsular/partial tonsillectomy; tonsillar ablation), implementation of guidelines, outpatient treatment forms, single cases, single prospective/retrospective mono-/multicenter studies, general risks of surgical measures in pediatric patients, medico-legal issues, effects on postoperative laboratory parameters, quality of life after different ENT-specific surgeries, different postoperative types of care in children, risk factors for postoperative failure, articles without abstracts, postoperative examination procedures, benefit of preoperative diagnostics, side effects of TE, publications not in German/English, actualization of a previous review article by the same first author, articles without references, malignomas, or treatment suggestions in cases of unilateral tonsillar augmentation.

### 3.2 Review articles on single diseases

#### 3.2.1 Otitis media with effusion

##### 3.2.1.1 Practice essentials of otitis media with effusion

The main symptom of otitis media with effusion is hearing impairment because of a collection of non-purulent fluid in the middle ear behind an intact tympanic membrane. Typically, there are no signs of inflammation and the patient is pain-free. If the collection consists of mucous secretion, the disease is called* glue ear* (mucotympanum). Otitis media with effusion occurs in up to 80% of the children until school age, the prevalence in the second year of life is 20%. Adults are affected more rarely. Regarding pathophysiology, the disease is based on a dysfuction of the Eustachian tube, almost always secondary adenoids in pediatric patients. But also different infection pathogens seem to play a role that lead to mucosal swelling of the tube and the middle ear. Besides, also other origins are mentioned such as allergy-induced occlusion of the tubal ostia, deviation of the nasal septum, hyperplasia of the turbinates, chronic sinusitis, cleft lip and palate, nasopharyngeal tumors, radiation, endocrinological reasons (myxedema), transnasal intubation, nasogastric tube, nasal packing, barotrauma, immune deficiency, gastro-esophageal reflux as well as ciliary dysfunction [43].

##### 3.2.1.2 Evaluation of the Cochrane Collaboration

There was no evaluation published by the Cochrane Collaboration.

##### 3.2.1.3 Evaluation of the Clinical Practice Guideline

In 2004, a committee consisting of the *American Academy of Pediatrics (AAP)*, *American Academy of Family Physicians (AAFP)*, and the *American Academy of Otolaryngology – Head and Neck Surgery (AAOHNS)* published a revised guideline on diagnostics and therapy of otitis media with effusion focussing on pediatric patients between 2 months and 12 years of age [44], [45]. The recommendation issued in 1994 was confirmed: symptomatic otitis media with effusion persisting for more than 4 months is no indication for TE, as well as myringotomy alone. The insertion of tympanostomy tubes is recommended [46], [47], [48], hearing improvement of 6 to 12 dB can be expected [49], [50]. Because of the higher invasiveness, adenoidectomy is recommended only in cases of clinically relevant symptoms [46], [51] or indicated revision surgery [46], [52], [53]. The basis for this assessment was the risk-benefit ratio influenced by the risk of postoperative bleeding and the evaluation of the following 3 trials [51], [52], [54].

##### 3.2.1.3.1 assessed in the Clinical Practice Guideline: Coyte

In this study of Coyte [52], the hospital discharge documents for 1992 through 1997 of patients up to the age of 19 from Ontario were identified who underwent at least one of the following interventions: myringotomy, myringotomy with the insertion of a tympanostomy tube, TE, adenoidectomy, or adenotonsillectomy (ATE). Records were excluded only if it was not possible to identify the patient’s place of residence (true for 0.7% of records) or if a valid patient identifier was not available, thereby preventing follow-up (true for 0.9% of records).

Children were included who had their first admission between 1995 and 1997 in which a tympanostomy tube was inserted, either as solitary or as combined intervention. A total of 37,316 interventions of 117 hospitals were evaluated. The common demographic data, facts about the surgeries and comorbidities (Charlson Comorbidity Index between 0 and 6) were assessed. In the subgroup analysis of 31,463 children, all comorbidities and procedures such as turbinectomy, correction of cleft palate, ear irrigation, lingual frenotomy, puncture of nasal sinus, and intranasal antrotomy were excluded. Two outcome measures were examined: the time to the first reinsertion of tympanostomy tubes and the time to the first readmission for a condition related to otitis media. Children receiving myringotomy/insertion of tympanostomy tubes as solitary intervention were younger than those receiving myringotomy/insertion of tympanostomy tubes as combined intervention. Adjuvant adenoidectomy (AT) was associated with a reduction in the likelihood of reinsertion of tympanostomy tubes. The risk was further reduced if an adjuvant adenotonsillectomy was performed (330 vs. 6,147), and there were broader confidence intervals (CI) (0.3–0.8 vs. 0.5–0.6). Readmission within the first postoperative year after insertion of tympanostomy tubes alone was observed in 10% of cases, after 2 years in 24% compared to 4% and 12% in the group with adjuvant AT. Additional TE further enhanced the effect, the relative risk for re-insertion as well as recurrent therapy was reduced of 20%. Recurrent therapies and re-insertions decreased with higher ages, other factors did not play a significant role. From the patients of 12 months or younger having undergone insertion of tympanostomy tubes, 5% received additional AT and 2% ATE, from the patients up to 3 years, those were 13% and 22%, respectively. AT in addition to insertion of tympanostomy tubes in children up to the age of 2 reduced the relative risk of recurrent therapy by 40% (relative risk: 0.6; 95% CI: 0.4–0.8; P<0.001). For ATE, better values could be achieved (relative risk: 0.5; 95% CI: 0.3–0.8; P=0.007), most significant in children of 3 years and older. Comparable results were found for the endpoint of re-insertion. Complications in the sense of nausea, vomiting, postoperative bleeding, and others were observed after insertion of tympanostomy tubes (0.2%), AT (0.5%), TE (0.5%), and ATE (2.6%), but they were not analyzed in detail. There were no deaths among the patients. Among the 91 children who were readmitted to hospital within 30 days after discharge, 43% were aged 12 months or younger. Rehospitalization occurred more frequently after solidary interventions than after adjuvant surgeries.

**Conclusion drawn by the authors:** Performing an AT or ATE at the time of the initial insertion of tympanostomy tubes substantially reduces the likelihood of additional hospitalizations and operations related to otitis media among children two years of age or older.

##### 3.2.1.3.2 assessed in the Clinical Practice Guideline: Paradise

Similar to his frequently cited trial on the effectiveness of TE, Paradise [51] established criteria encompassing the following aspects: age between 3 and 15 years, no previous surgery, at least 3 episodes of acute otitis media during the preceding 6 months, or at least 4 episodes during the preceding 12 months including at least 1 episode during the preceding 6 months, with at least 1 of the episodes having been documented with a recorded description of symptoms and tympanic membrane findings or confirmed by tympanometry, or myringotomy; or middle-ear effusion in 1 or both ears extending over at least 180 days during the preceding year and documented by at least 2 clinical observations.

Of 2,122 children evaluated, 461 met the eligibility criteria. They were stratified into 3 age categories (age 3 and 4, 5 and 6, and 7 to 15 years) and further classified by means of clinical and radiographic criteria as to whether they had adenoidal nasal obstruction. The children were then assigned randomly, within age and nasal obstruction categories, within 1 of 2 clinical trials. Separate, computer-generated random number lists were used for the assignments. 

In one trial, 304 children without apparent tonsil-related indications for TE were assigned, in balanced blocks of 6 subjects, to 1 of 3 treatment groups: AT, ATE, or control (the 3-way trial). In the other trial, 157 children whose tonsils appeared potentially obstructing or who had a history of recurrent throat infection that met or exceeded entry criteria used in previously reported or concurrent TE trials were assigned, in balanced blocks of 4 subjects, to 1 of 2 treatment groups: ATE or control (the 2-way trial). 

In patients who had middle ear effusion at the time of surgery, myringotomy was performed. For all surgical subjects, the trial starting point was the first postoperative day. In the control group, the trial starting point was the day after assignment for effusion-free patients; for those with effusion, it was the first effusion-free day. Control subjects in whom effusion had been present for 90 days or longer without improvement underwent myringotomy and aspiration, and their trial starting point was the following day.

Follow-up procedures included biweekly inquiries about day-to-day status and clinical assessments by study-team pediatric nurse practitioners and/or pediatricians using standardized procedures and algorithms at 6-week intervals; at the time of acute illnesses and for episodes of otitis media, at 1- to 4-week intervals until resolution. Pneumatic otoscopes with airtight lens assemblies were used for examining the tympanic membrane. When the membrane was intact, the diagnosis of otitis media and its classification as either acute or otitis media with effusion were based on criteria reported previously. Tympanometry was performed at most visits. Doubtful otoscopic diagnoses were decided by an otolaryngologist on the basis of otomicroscopic examination. Audiometry was performed at the time of trial entry, at the first postsurgical visit, during and following episodes of otitis media as deemed clinically advisable, and at maximum intervals of 6 months. For each subject the cumulative proportions of days were estimated on which unilateral and bilateral otitis media, respectively, were present, based on diagnoses at individual visits and interpolations for intervals between visits provided that the intervals did not exceed 90 days when the otitis status on the 2 visits was the same. In all subjects, each new episode of otitis media of any type was treated with an antimicrobial in conventional dosage for 10 days to 6 weeks, depending on recent clinical course and response to treatment. Amoxicillin was used whenever feasible; second-line drugs mainly used were erythromycin-sulfisoxazole and amoxicillin-clavulanate potassium. When middle-ear effusion persisted for 90 days without improvement, myringotomy with aspiration was performed. When effusion recurred within 6 months after myringotomy and persisted for 60 days without improvement, myringotomy with tube placement was performed. Secondary otorrhea was treated with an antimicrobial orally for up to 2 weeks, and if persistent thereafter, with polymyxin B-neomycin-hydrocortisone ototopical suspension. The primary outcome measure was the number of episodes of acute otitis media within a follow-up year. Secondary measures were the estimated proportion of time with otitis media, the numbers of myringotomies and tube procedures, and the numbers of days, respectively, on which ear pain occurred and antimicrobial treatment was received.

In total, the records of 410 children could be evaluated, 266 from the 3-way trial and 144 from the 2-way trial. 354 (86.3% were follow-up for at least one year, 308 (75.1% for at least 2 years, and 250 (61.0%) for 3 years. 374 (91.2%) were eligible on the basis of recurrent acute otitis media, 22 (5.4%) on the basis of persistent middle-ear effusion, and 14 (3.4%) on the basis of both conditions. However, of the 374 subjects enrolled on the basis of recurrent acute otitis media only, 119 (31.8%) had prior histories of middle-ear effusion of at least 2 months’ duration and 189 (50.5%) had middle-ear effusion at entry that had not been previously diagnosed. The only significant difference in the subpopulations was registered in the 2-way trial, in which the ATE group contained proportionately more girls and fewer boys than the control group (40% vs 58%; P=0.04). In the 3-way trial, primarily in the ATE group, socioeconomic status was higher among subjects who completed 3 years of follow-up than among subjects who did not. 

The median and mean intervals from assignment to starting point in the 3-way trial were 73 and 84.0 days, respectively, among AT subjects; 60 and 66.4 days, respectively, among ATE subjects; and 8 and 22.6 days, respectively, among control subjects. Corresponding values in the 2-way trial were 60 and 63.3 days, respectively, among ATE subjects; and 0 and 19.0 days, respectively, among control subjects.

Differences in outcome between the groups in the 3-way trial were generally small. The mean rate of episodes of acute otitis media in AT subjects was actually higher than in control subjects in the second follow-up year. In ATE subjects the rate was lower than in control subjects in the first follow-up year and for the 3 follow-up years combined. The mean rate also was lower than in AT subjects in the first follow-up year and for the 3 follow-up years combined. Both AT subjects and ATE subjects had, on average, less estimated time with otitis media than control subjects in the first follow-up year but not thereafter. The first-year difference between ATE and control subjects also was responsible for a difference between these 2 groups for the 3-year follow-up period as a whole. During the first follow-up year ATE subjects received, on average, less antimicrobial treatment than control subjects; no other between-group differences during the 3-year follow-up period were significant.

Differences in outcome in the 2-way trial again were generally small. Differences favoring ATE subjects over control subjects in the mean rate of episodes of acute otitis media were not significant in any individual follow-up year, but for the 3 follow-up years combined the difference was significant. ATE subjects had, on average, less estimated time with otitis media than control subjects during the first follow-up year; this resulted in a difference between the 2 groups for the 3 follow-up years combined. During the first and second follow-up years, ATE subjects received less antimicrobial treatment than control subjects, but no other differences during the 3-year follow-up period were significant.

In both the 3-way and 2-way trials, among control subjects who eventually underwent surgery, mean rates of acute otitis media and estimated proportions of time with otitis media during the segments of follow-up years that preceded the surgery were modestly higher than the corresponding whole-year values among control subjects who remained under surveillance without change in status. Outcomes were generally less favorable in younger than in older subjects and in subjects with bilateral effusion at the time of assignment than in subjects with unilateral or no effusion. However, surgical-vs-control outcomes did not change substantially after adjusting the therapy individually. Analysis indicated that hearing acuity was related consistently only to whether otitis media was present and not to subjects' treatment groups. Postoperative sore throat in AT subjects was 1.1 days (range, 0–7 days), and in ATE subjects 5.8 days (range, 0–21 days). Postoperative hemorrhage only occurred in 2.2% after ATE. The benefit of surgery is estimated as moderate and limited to the first postoperative year. The highest difference could be observed in the 3-way trial between the ATE group and the control group. The mean annual number of episodes was 1.4 vs. 2.1 (p<0.001) and the mean time with effusion was 18.6% vs. 29.9% (95% CI: 4.4–18.2%; p=0.002).

**Conclusion drawn by the authors:** Because of the cost-benefit risk as well as the postoperative morbidity neither AT nor ATE are justified as adjuvant interventions in children with effusion without further disease symptoms.

##### 3.2.1.3.3 assessed in the Clinical Practice Guideline: Maw

The study of Maw was conducted during July 1979 to March 1982 [54]. At regular intervals throughout the period 103 children aged between 2 and 11 years suffering from bilateral otitis media with effusion were examined. At the first appointment the conditions and trial were explained and an antihistamine-sympathomimetic amine mixture (Dimotapp elixir) in appropriate dosage for age prescribed until the second appointment six weeks later. Repeat examination and investigations were then performed to confirm the presence of bilateral otitis media with effusion. For the next six weeks no treatment was prescribed. If fluid in the middle ear was still present on both sides, the child was admitted to hospital within two weeks for operation. During this time a lateral cephalometric radiograph of the nasopharynx was taken. Surgery to the tonsils and adenoids was randomly allocated (procedure not further described), as follows: ATE 34 cases; AT 36; no surgery 33. Additionally, in all cases on a randomly allocated basis (randomization is not described) unilateral myringotomy was performed. The children were re-examined six weeks, three months, six months, nine months, and one year after surgery. Clinical examination was performed in all cases together with pure tone audiometry and impedance studies. The unoperated ear was assessed for fluid in the middle ear with a Siegle's pneumatic otoscope.

Compared with the no-surgery group the effect of AT alone after one year was highly significant (p<0.001), and similarly the effect of ATE was significant (p<0.01). There was, however, no increased benefit from the addition of TE compared to AT alone. Thus there was resolution of 36–46% of chronic effusions as a result of AT. Only one patient was lost to follow-up after three months. The effect of AT one year after surgery was still significantly higher than after conservative therapy (p<0.01). CI were not given.

**Conclusion drawn by the authors:** Adjuvant AT significantly increases the effect of myringotomy, additional TE does not further increase the benefit.

##### 3.2.1.4 Table and conclusion on the indication of otitis media with effusion

See Table 2 (Tab. 2).

**Conclusion:** TE is not appropriate for treating otitis media with effusion.

#### 3.2.2 IgA nephropathy

##### 3.2.2.1 Practice essentials of IgA nephropathy (IGAN)

In 1968, Berger und Hinglais were the first to describe this disease [55]. Histologically, the deposition of immunoglobulin A (IgA) can be observed in the mesangium of the diseased glomeruli. It is the most frequent primarily glomerular disease that in 30–40% leads to terminal renal failure within 20 years. The clinical key symptoms range from low persisting microscopic hematuria to recurrent episodes of macroscopic hematuria, mostly associated with infection of the upper airways. Hematuria is often accompanied by mild proteinuria (0.5–2 g per day). More rarely, nephrotic proteinuria occurs (>3 g/24 h). IGAN is observed more frequently in male than in female patients, and peaks at an age between 16 and 35 years. Another clinical key symptom is arterial hypertension in more advanced stages. In the early stage of the disease, often a nearly regular or only mildly impaired kidney function is observed. For diagnosis, renal biopsy with examination of the specimen by light, immune, and electron microscopy is mandatory. The most important physiological function of the IgA molecule is the defense of inhaled or transoral contamination with antigens. For this purpose, IgA globulin synthesized in the tonsils is bound to the so-called secretory component. The secretory component develops in the epithelial cells and is responsible for the transcellular transport of the IgA. The secretory IgA globulin is present as polymer IgA and mostly consists of IgA1 and IgA2 molecules. IgA identified in the serum compartment is synthesized in the plasma cells of the bone marrow. It is mainly monomer and consists predominantly of IgA1. Between both compartments, there is usually no significant exchange. Patients with IGAN only have IgA1 in the glomeruli, which is present in polymer form, i.e. bound by a J chain. This indicates that the IgA in the serum compartment plays an important role for the development of the IgA immune complex in the glomeruli. It is supposed that patients with IGAN have a disorder of the secretory IgA component generally defending infections and that then infections of the upper airways or the gastro-intestinal tract lead to a stimulation of the new synthesis of IgA in the bone marrow. In the bone marrow, there seems to be an additional defect of the stem cells that is not characterized in detail up to now which is responsible for the overproduction of IgA. Beside the immunosuppressive glucocorticoid therapy (1 mg/kg/d), adjuvant therapies with fish oil (1.8 g/d or eicosapentaenoic acid; 1.2 g docosahexaenoic acid), ACE inhibitors, and antihypertensive agents turned out to be effective. Additionally, prophylaxis must be started in time to cope with the developing secondary hyperparathyroidism, vitamin D deficiency, renal anemia and acidosis, dyslipidemia and dietetic guidance of the patients. These recommendations are taken from a publication by Thaiss and Stahl published in 2000 without even mentioning TE as therapeutic option [56]. Regarding pathogenesis, still a dysregulation between mucosa and bone marrow is assumed with resulting auto-antibody production and deposition in the glomeruli. Tonsillitis episodes seem to have a trigger function so that TE might have a prophylactic benefit [57]. Recurrences after kidney transplantation of healthy individuals [58], the disappearance of IgA immune complexes after kidney transplantation of sick subjects [59], and the detection of IgA complexes in the skin, gut, and lung [60] support this assumption.

##### 3.2.2.2 Evaluation of the Cochrane Collaboration

In the meta-analysis of Reid et al. from 2011, different, non-immunosuppressive therapies of IGAN were assessed, among others also including TE [36]. The authors come to the conclusion that there is no RCT that proves the benefit of TE. With their research of the literature, the authors had identified 2 publications from Japan with a total of 79 patients [61], [62]. The data of 77 patients were eligible for calculations. The only significant observation was a normalization of the hematuria in patients having undergone TE (relative risk: 1.54; 95 CI: 1.05–2.25). This was mainly due to the significant effect on the microscopic hematuria [62] (relative risk: 1.83; 95% CI: 1.04–3.22). The macroscopic hematuria was not significantly changed by TE [61] (relative risk; 95% CI: 0.80–2.23). Heterogeneity index I²: microscopic hematuria and proteinuria 0%; creatinine clearance 76%.

**Conclusion drawn by the authors:** The data situation is insufficient to justify TE.

##### 3.2.2.2.1 assessed in the review article of the Cochrane Collaboration: Kawasaki

In this trial, 32 patients were included who had not received immuno-suppressive pretreatment with bioptically confirmed first diagnosis of IGAN. After the 2-year follow-up period, they were 15 years old or younger. A second biopsy was performed in the course (average 24.8 ± 2.3 months after treatment onset). Histologically, an activity index was determined as the sum of the following aspects: mesangial proliferation (grades 0–3; normal=0, slight=1, moderate=2, severe=3), interstitial mononuclear cell infiltration (none=0; 1%–20%=1; 21%–50%=2; >50%=3), and crescent formation (grades 0–3 according to the proportion of glomeruli involved: none=0; 1%–20%=1; 21%–50%=2; >50%=3). The chronicity index (CI) was determined as follows: the number of glomeruli demonstrating fibrous crescents or segmental or global sclerosis was counted and each scored as 0–3, according to the proportion of glomeruli involved (none=0, 1%–20%=1, 21%–50%=2, >50%=3); tubular atrophy and interstitial fibrosis were graded as 0–3. The sum of these numbers was the chronicity score (maximum=12). The histological sections were reviewed by two independent investigators. The patients came to the hospital for examination of upper airway infections (fever, sore throat, rhinorrhea, or cough) in order to undergo blood and urine examination. The patients were randomly assigned to 2 therapy groups. Group A underwent TE and methylprednisolone pulse therapy regardless whether they had a history of tonsillitis (three courses, each consisting of high–dose methylprednisolone, 20–25 mg/kg/day for 3 days per one) with methylprednisolone therapy starting about 10 days after TE. Prednisolone was given orally at a dose of 2 mg/kg body weight per day in three divided doses, not exceeding a total dose of 60 mg/day, for 2 weeks, followed by 1.5 mg/kg per day for 2 weeks, 1.0 mg/kg per day for 4 weeks, 0.5 mg/kg per day for 4 weeks, 1.0 mg/kg per 2 days for 9 months, and 0.5 mg/kg per 2 days for 21 months. Warfarin was given orally in a single morning dose of 1–2 mg/day. Dipyridamole was given orally in a dose of 5 mg/kg body weight per day (max. 300 mg) in three divided doses for 24 months. The patients assigned to group B did not undergo TE plus pulse therapy and were treated with PWDM alone. PWDM consisted of the above PWD regimen plus MZB. MZB was given orally, 5 mg/kg body weight per day in two divided doses, for 24 months. All 16 patients in group A and 16 of the 18 patients in group B completed the trial. Patients were tested for proteinuria by quantitative determination of protein in 24-h urine specimens. “Diffuse mesangial proliferation” was defined on the basis of the World Health Organization criteria as more than 80% of the glomeruli showing more than three mesangial cells per peripheral mesangial area. The clinical classification of the stages was as follows: stage 0: Normal findings of the physical examination, and the patient had normal urine and normal renal function. Stage 1 represented minor urinary abnormalities: the results of the physical examination were normal, but the urinalysis revealed microscopic hematuria or proteinuria less than 20 mg/m²/h. Stage 2 meant persistent nephropathy: the patient had 20 mg/m²/h or greater proteinuria, and 24-h creatinine clearance (24-h Ccr) was 60 ml/min/1.73 m² or greater. Stage 3 was a renal insufficiency: the patient had a 24-h Ccr value less than 60 ml/min/1.73 m². Tonsillitis was defined as reddening and swelling of the tonsils or a membranous exudate on the tonsils in patients who experienced symptoms of an upper respiratory tract infection, such as fever, sore throat, rhinorrhea, or cough. Acute exacerbation of IGAN by tonsillitis was defined as an increase in the severity of the patient’s clinical status by more than one stage. The therapy groups were comparable with regard to epidemiological and clinical data, the time interval between the onset of study and the last follow-up examination was 36.1±7.9 (group A) and 37.6±8.5 months (group B). Proteinuria varied between 40–170 mg/m²/h (average 97±45 mg/m²/h) in group A and 41–195 mg/m²/h (average 93±36 mg/m²/h) in group B. The value of creatinine clearance was low in 6 patients of group A and 5 patients of group B. In both groups, the average renal protein loss was significantly reduced after 6 months (group A: from 97±45 to 26±15 mg/m²/h; group B: from 93±36 to 25±17 mg/m²/h; p<0.05). After 24 months, the values were further reduced with 13±12 in group A and 12±8 mg/m²/h in group B. The difference between both therapy groups was not significant. After 6 and 24 months, none of the patients had low values of creatinine clearance (<60 ml/min/1.73 m²). In both groups the parameters of proteinuria and hematuria were improved, the comparison of both groups revealed no significant difference in the stages. The mean protein loss amounted to 8±8 mg/m²/h in group A and 10±8 mg/m²/h in group B. In stage 0 or 1, there were 12 and 4 patients of group A and 9 and 6 patients of group B, respectively. None of the patients had stage 3 or 4 at the end of the trial. The activity index in both groups was positively influenced by the therapy with a statistical significance (3.9±0.8 vs. 6.8±1.9 in group A; p<0.01; 4.1±1.0 vs. 6.6 ±1.4 in group B, p<0.01). Regarding the chronicity index, no therapeutic effect could be observed in both groups. In group A, 14 patients had a peritonsillar abscess (PTA), otherwise all had “chronic tonsillitis”. There were no significant differences between the groups in the incidence of cushingoid changes, glaucoma, or arterial hypertension. Only three patients in group B developed hyperuricemia, but it was well controlled by treatment with allopurinol. Six patients in group B experienced an acute exacerbation of IGAN as a result of tonsillitis (p<0.05). In their conclusion, the authors indicate a positive effect of the therapy regimen that is, however, without difference. There was only one difference in the tonsillitis-associated exacerbation in 6 patients of group B. To some extent, TE seems to play a prophylactic role.

**Conclusion drawn by the authors:** There is no additional therapeutic benefit of adjuvant TE.

##### 3.2.2.2.2 assessed in the review article of the Cochrane Collaboration: Hotta

Forty-five patients with biopsy-proven active IGAN and chronic tonsillitis were included in this study [62] with a follow-up period of 3 years. The diagnosis of “chronic tonsillitis” was made by an otolaryngologist, the criterion was exclusively pus in tonsillar cysts. The patients were divided into 2 groups; the two groups were well matched in terms of age, gender, the duration and stage of the disease before the onset of treatment. In group A (n=19), the patients were treated with cortisone (1g/d; 1–3 times, then 30 mg/d orally for 4 weeks followed by a gradual decrease over 1–2 years), cyclophosphamide (50 mg/d for 4 months), dipyridamole (150–300 mg/d administered throughout the observation period) and warfarin (thrombotest 20–40% during cortisone therapy). In addition to the same regimen in group A, the patients of group B (n=26) underwent TE prior to medication. In monthly intervals, the following parameters were measured: creatinine clearance, 24 hour urinary protein excretion, urine sediment erythrocytes, serum total protein, serum creatinine concentration, and blood pressure. Normalization of the urinary values for at least 3 months was defined as remission (hematuria: <3 erythrocytes/high power field; proteinuria: <100 mg/d). Patients requiring antihypertensive agents during the observation period, were excluded from the study. The pre- and post-therapeutic blood pressure were not different in both groups A and B. While the pre- and post-therapeutic creatinine clearance did not change in group A, it significantly improved in the TE group from 80.5 ml/min to 92.1 ml/min (p<0.05). In group A, proteinuria decreased from 1.1 g/d to 0.6 g/d and in group B from 1.5 g/d to 0.4 g/d. Proteinuria could no longer be detected in 5 patients of group A and in 14 patients of group B after the end of the observation period, however, there was no statistical significance. With regard to hematuria, 8/19 patients of group A and 20/26 patients of group B were inconspicuous after the end of the observation period, according to the authors, TE led to a significant difference within the 2-year follow-up time. The authors concluded that in both therapy arms proteinuria was reduced (group A: 26.3%, group B: 76.9%), and the kidney function could be significantly improved by adjuvant TE. The type of randomization remains unclear, the reference of 17 instead of 19 patients in the text as well as the different observation period of 2 and 3 years in the evaluation cannot be understood from the text.

**Conclusion drawn by the authors:** The therapeutic benefit can be increased by adjuvant TE in patients with active IGAN.

##### 3.2.2.3 Assessment by other systematic review articles or meta-analyses

##### 3.2.2.3.1 assessed by Liu

In this meta-analysis performed by Liu et al. [57] the above-mentioned publications were not included because of different inclusion and exclusion criteria. In this article, all controlled trials as well as retro-/prospective cohort studies limited to adults were included comparing therapy with and without TE. As primary endpoint, the remission (according to the individual definition of each study) and as secondary endpoint the end-stage renal disease (serum creatinine >8 mg/dl; hemodialysis, kidney transplant) were defined. Pretreated patients and patients with secondary IGAN were excluded from the trial. Fourteen of 428 publications were eligible for the meta-analysis. Most of them had a retrospective study design [63], [64], [65], [66], [67], [68], [69], [70], [71], [72], [73]; others were one prospective, non-randomized, but controlled study [74], one prospective cohort study [75], and one randomized-controlled trial [76]. The size of the patient populations varied between 41 and 388 patients, 16–250 of them underwent TE. The size of the control groups varied between 15 and 148 patients. All patients of the intervention group underwent TE, 7 received adjuvant cortisone pulse therapy [63], [64], [66], [71], [74], [75], [76]. The age of the patients varied between 27.3 and 46.5 years, 32.3–72.9% were male. The serum creatinine values and the values of glomerular filtration were comparable, apart from one exceptional case (3.45 g/d [65]) also the basic proteinuria (0.31–1.81 g/d) that was not mentioned by only one group of authors [73]. The follow-up varied between 12 and 197 months. Clinical remission was reported in 10/14 trials with 1431 patients, the odds ratio (OR) was 3.4 (95% CI: 2.58–4.48; p<0.001), so that a positive effect of TE could be confirmed. This benefit also persisted, if studies with unclear or uncommon therapy with ACE inhibitors were excluded and the calculation for the remaining 671 patients was performed [65], [66], [68], [75]: the OR changed to 2.80 (95% CI: 1.91–4.09; p<0.001). For the 7 studies with additional cortisone pulse therapy in 783 patients [63], [64], [66], [71], [74], [75], [76], the calculations revealed an OR of 3.15 (95% CI: 1.99–5.01; p<0.01). For 2 trials with conventional cortisone therapy in the intervention group [68], [72] an OR was calculated for 159 patients with 4.13 (95% CI: 1.23–13.94; p=0.02). In 3 studies, adjuvant therapy in the intervention groups consisted of the administration of agents such as antihypertensive, anticoagulant drugs, lipid reducers, immune-suppressive agents, or cytostatic drugs [66], [72], [75]. Also for this subgroup analysis, TE was associated with a clear benefit, the OR was 2.21 (95% CI: 1.2–4.05). With regard to the endpoint of end-stage renal disease, 9 studies with 973 patients were eligible for the analysis [65], [67], [68], [69], [70], [71], [73], [74], [76]. The risk could be significantly reduced by TE with an OR of 0.25 (95% CI: 0.12–0.52; p<0.001). In a subgroup analysis, all studies with a follow-up period of less than 5 years were excluded and analog calculations for the remaining 691 patients performed [65], [67], [68], [69], [70], [71]. However, there was no difference regarding the aforementioned statement, the OR changed to 0.2 (95% CI: 0.11–0.36). Even after exclusion of studies with uncommon or unclear studies. Furthermore, a generalization of the results is probably difficult because most of the trials came from Asia, in particular Japan. The calculation of the odds ratio instead of the hazard ratio could be problematic because of the different follow-up periods, but this affects intervention and control groups at the same time.

**Conclusion drawn by the authors:** TE as adjuvant but also as solitary therapy is beneficial for remission and prophylaxis of end-stage renal disease.

##### 3.2.2.3.2 assessed by Wang et al.

In contrast to Liu [57], some studies were not included in the meta-analysis by Wang et al. [77] just because of the publication date; TE was not analyzed as independent factor and the heterogeneity of the studies was not mentioned such as the different therapy with ACE inhibitors or different follow-up periods. From 127 hits of the literature research, the authors selected 7 studies with a total of 858 patients with a controlled study design and a follow-up period of at least 36 months, and investigated patients between 15 and 60 years who did not suffer from other basic diseases. In none of the studies, the patients were randomly assigned to one therapy arm [67], [68], [69], [71], [72], [74], [75]. 534 patients had undergone TE, 324 patients were in the control groups. The authors defined remission as the condition of missing hematuria, proteinuria of <1.5 g/d, ≤4 erythroytes/high power field in the urinary analysis, and a regular kidney function. If those parameters were not fulfilled, >10,000 urinary erythrocytes/ml were found as well as a serum creatinine value of ≥1.3 mg/dl, remission was excluded. The end-stage renal disease was defined as a serum creatinine value of >8 mg/dl or hemodialysis or kidney transplant. In all studies, the follow-up period was 5 years or more, and a significantly positive therapeutic effect proven. Since the trials were already discussed in the previous chapter, a description of the statistical calculations (OR, CI) is not made here. The authors could also prove a significant therapeutic benefit due to TE with regard to avoiding end-stage renal disease and a remission after 5 and 10 years. Cortisone pulse therapy was superior to conventional cortisone application if it was combined with TE. The combined therapies were superior to monotherapy (conventional cortisone or pulse therapy). There was no difference between TE alone and general measures (ACE inhibitors, cytostatic agents, dipyridamole). Only when TE was performed in combination with cortisone pulse therapy, the patients benefited significantly.

**Conclusion drawn by the authors:** TE should only be performed as adjuvant therapy in combination with other treatment modalities. Combined therapy is superior to monotherapy. The data situation does not allow an estimation of the prophylactic value of TE.

##### 3.2.2.3.3 assessed by Wyatt and Hogg

This systematic research of the literature [78] aimed to limit the review to children and adolescents and included Henoch-Schönlein purpura nephritis as well [78]. For the mentioned age groups, the development of chronic or end-stage renal disease is very rare so that therapy at first diagnosis has a more important prophylactic task. However, since it was not possible to set the endpoint of end-stage renal disease because of the long course of the disease, other endpoints such as hematuria/proteinuria were accepted. The explanation of the research strategy is inadequate and there are several mistakes in citations or data transfer. In context of IGAN, the authors deal with studies with most different therapeutic options (cortisone, fish oil, ACE inhibitors) in adults and children and list 6 [79], [80], [81], [82], [83], [84] of 11 publications [73], [79], [80], [81], [82], [83], [84], [85], [86], [87], [88] identified for TE. Those studies were published between 1985 and 1999 and correspond to 4 case series and 2 retrospective case control studies with populations of 7–45 patients.

**Conclusion drawn by the authors:** Explicitly for children there are only few data, the indication of TE in this age group cannot be justified.

##### 3.2.2.3.4 later excluded: Nolin and Courteau

The authors [89] performed a research of the English and French literature starting in 1976 on different therapeutic options for IGAN (cortisone, cyclophosphamide, dipyridamole, warfarin, cyclosporine, fish oil, azathyioprine cortisone) and for the management of arterial hypertonia [89]. The description of the research strategy is inadequate and the authors do not explain according to which classification they performed the literature assessment (levels I–IV are mentioned) and how recommendations resulted (grades A to D are listed). Regarding the publication by Béné et al., the authors only state that in patients with recurrent infections TE could reduce proteinuria, hematuria, and IgA serum concentration in 34 patients, which however had no impact on the renal function. The authors recommend TE in cases of recurrent tonsillitis with grade D.

##### 3.2.2.4 Table and conclusion on the indication of IGAN

See Table 3 (Tab. 3).

**Conclusion:** TE is probably appropriate for the treatment of IGAN.

#### 3.2.3 Sleep-related breathing disorders

##### 3.2.3.1 Practice essentials of sleep-related breathing disorders (SBD)

The symptoms of obstructive sleep apnea syndrome (OSAS) are explained by the term of SBD: disturbed breathing patterns during sleep with prolonged interruption of breathing by partly or complete obstruction of the upper airways. The prevalence is estimated to be 1.2–5.7%. In children, daytime sleepiness is not typically present, but frequently snoring (≥3 nights/week), enforced breathing during sleep, wheezing breathing, phases of apnea, secondary enuresis (after acquired continence for more than 6 months), sleeping in sitting position or hyperextension of the neck, cyanosis, headache on waking up, attention deficit/hyperactivity syndrome, and learning problems. Examination of the children often reveals over- or underweight, adenoid facies, micro-/retrognathia, high gothic palate, failure to thrive, and arterial hypertension [90]. Even if polysomnography (PSG) is considered as gold standard to verify OSAS, in most cases the diagnosis is established on a clinical basis [91]. But the significance of the parents’ reports is clearly lower than PSG [92]. The success rate of ATE varies enormously between 24.2% and 100% [93], [94], [95], [96], [97] and is biased by numerous parameters (body weight, age, gender, neuromuscular and syndromic basic diseases), especially by the inconsistent use of a cut-off value for a pathologic pediatric apnea-hypopnea index (AHI). Successful outcome after surgery was rated by some only if the AHI is < 1, others choosed <2 or <5 as cut-off value. Moreover, PSG was not repeated to verify the “success” in every study [98]. The therapeutic benefit of ATE is superior to AT alone or TE alone [99], [100]. This is certainly true for the typical age (average 4 to 8 years [101]) and turned out to be effective also for younger children between 3 and 24 months [102]. But also AT alone can significantly reduce the AHI in children between 5 and 12 months [103]. According to the literature research by Garetz [104] and Baldassari et al. [105], the quality of life, the behaviour, and the perception could be improved by ATE in children with OSAS. However, the data situation is considered as very heterogenic so that prospective, larger multicenter trials are necessary to confirm these results.

##### 3.2.3.2 Assessment by the Cochrane Collaboration

In August 2010, Lim and McKean performed an update of the literature research [35]. They analyzed the outcome after AT, TE, and ATE for OSAS confirmed by PSG or pulse oximetry in children (≤16 years of age) in randomized trials. They were compared to other medical, mechanical, or no interventions. An AHI >1 and an AHI >5 in PSG or deoxygenation in pulse oximetry in relation with SDB was defined as inclusion criterion. Primary endpoint of the research was the AHI, additionally different secondary endpoints were reviewed (daytime sleepiness, deoxygenation, lowest oxygen saturation, relative decrease of oxygen, average deoxygenations per hour, average duration of deoxygenations, quality of life, postoperative morbidity/complications/mortality/physical size and changes of the body weight as well as one-year mortality). Among 239 hits, only one single publication was eligible for analysis [106]. The data evaluation was not possible in 4 of 27 children because they did not appear for surgery. Uncertainties regarding the transferability of the OSAS classification of adults to children made it difficult to assess the pediatric disease as well as to evaluate short-term apnea of different severity in those ages. The only significant difference in comparison of both surgical procedures was the postoperative weight. After radiofrequency-induced reduction of the tonsillar volume, the children were able to return to normal diet 7 days after surgery (95% CI: 4.14–2,253–2,253.15) and gained weight more rapidly (95% CI: 0.23–3–3.01). There were no differences regarding the secondary endpoints such as daytime sleepiness (95% CI: –2.17–2–2.17), snoring (95% CI: –0.8–1–1.05), speaking (95% CI: –3.29–0.89), and swallowing (95% CI: –0.50–1–1.30).

**Conclusion drawn by the authors:** ATE as well as radiofrequency-induced reduction of the tonsillar volume with simultaneous are comparably beneficial for pediatric OSAS. The initiation of further studies to evaluate the superiority of surgical procedures is recommended and there is a need for research regarding the definition and staging of pediatric OSAS as well as the location of the airway obstruction. The scientific data suggest, that surgery should be indicated with great care.

##### 3.2.3.2.1 assessed in a review article of the Cochrane Collaboration: Coticchia

The population of this study [106] comprised 23 previously untreated pediatric patients aged 2.6 to 12.5 years with a body mass index of less than 30 and symptoms of upper airway obstruction due to hypertrophic adenoids and tonsils who underwent radiofrequency-induced reduction of the tonsillar volume (n=13; AHI 7.6) or TE (n=10; AHI 7.7) with simultaneous AT, respectively. Exclusion criteria were acute or chronic lung disease, Down syndrome, neurological, speech, or swallowing disorders, craniofacial abnormalities, an AHI >30, or comorbidities. All children underwent preoperative PSG and were randomly assigned to one of the two surgical procedures one week before the planned intervention (the randomization procedure is not further described). The tonsillar size was classified according to Brodsky and photo-documented. The quality of life was assessed by means of a questionnaire and all patients received amoxicillin for 3 days (40 mg/kg three times per day; in cases of allergy they received clindamycin 25 mg/kg four times per day). Before application of the radiofrequency probe, 5.0 ml of 0.25% bupivacaine HCl without epinephrine were locally injected and 5 ml physiological saline solution was applied into the tonsil tissue. Five to seven insertions of the radiofrequency probe were performed per tonsil (average 12.6 ±1.50 insertions per patient with 994.68±91.88 J per insertion). During surgery, the children received intravenous dexamethasone (0.5 mg/kg; maximum dose: 20 mg) as well as 6 and 12 hours after the intervention. Regarding injections into the palatal arches/tonsils and administration of dexamethasone the procedure was exactly the same as in the control group; the dissection was performed by means of monopolar electrosurgery, however, without standardized settings. These patients were treated with amoxicillin or clindamycin for 7 days and received acetaminophen and codeine (0.5–1.0 mg/kg codeine every 4 hours) for 7 days. The patients/parents were asked to fill out a questionnaire every day and then 4, 8, 12, 24, and 52 weeks after the intervention in order to collect data on pain, diet, pharmaceutics intake, activities, and signs of airway obstructions and to undergo follow-up examinations. PSG was repeated 3 months after the intervention and revealed an AHI reduction of 5.6 after radiofrequency application and 6.5 after conventional ATE. The stage of the tonsillar size was reduced by 1.7±0.8 on average, which corresponds to a volume reduction of 57%. On the 4-point visual analogue scale, the patients treated with radiofrequency complained about mild pain (n=10), moderate pain (n=1) or were free of pain (n=2). In the control group, pain was rated as severe (n=1), mild (n=5), or moderate (n=4). After one week, the patients of the first group had only mild pain (n=6) or were free of pain (n=7). After conventional surgery, one patient experienced severe pain. Daytime sleepiness, snoring, speaking, and swallowing was improved in both groups, however, there was only a clear difference regarding speaking since the result after conventional surgery was poorer than after radiofrequency technique. In the radiofrequency group, the weight loss was 0.45±1.58 kg after 7 days compared to 2.07±1.76 in the control group. On day 7 after radiofrequency intervention, 11/13 patients could eat normally, 2 preferred soft food, and one child only had liquid food. In contrast, none of the children in the control group ate normal food but liquid (n=3) and soft food (n=10).

**Conclusion drawn by the authors:** Apart from an earlier pain-free period after tonsillar reduction by radiofrequency, both surgical procedures had a comparable outcome.

##### 3.2.3.3 Assessment by other systematic review articles or meta-analyses

##### 3.2.3.3.1 assessed by Wang et al.

This meta-analysis [107] was limited to the English literature and compared the outcome of TE vs. TOTO for OSAS in pediatric patients (≤18 years). Exclusively prospective studies with at least 10 patients were included and a last update was carried out in 2014. The authors assessed the quality of the literature based on the Newcastle-Ottawa score and performed statistical calculations with the Review Manager Software of the Cochrane Collaboration. Out of 199 publications, the authors selected 10 [108], [109], [110], [111], [112], [113], [114], [115], [116], [117]; 5 of them had a randomized design [108], [109], [110], [112], [116], the others had no random assignment of the patients [111], [113], [114], [115], [117]. In total, there were 459 patients for TOTO and 570 patients for TE. Regarding TOTO, the populations varied between 14 and 88, regarding TE between 15 and 133 patients. On average, the patients were 5.37 years old (2–15; TOTO: 4.95; TE 5.79). The mean follow-up time was 22.18 months (10 days to 72 months). The endpoints such as postoperative bleeding risk [108], [111], [112], [114], [115], [117], OSAS recurrence [108], [109], [111], [112], [115], [117], duration of surgery [108], [114], [115], [117], results of PSG [114], [117], and quality of life [112], [113] were statistically analyzed. The relative postoperative bleeding risk after TOTO in comparison to TE was 28% (95% CI: 0.1–0.78; p=0.01). In contrast, the relative risk of recurrence after TOTO in comparison to TE increased by factor 3.33 (95% CI: 1.62–6.82; p=0.001). This effect was even more obvious when only the non-randomized trials were evaluated: the relative risk increased even by factor 12.56 (95% CI: 2.28–69.15; p=0.004). There were no significant differences regarding the outcome calculating the following endpoints: immune function measured with the parameters of IgA, IgM, and IgG (relative risks: –0.06, –0.08, –0.3, respectively), results of PSG (relative risk: –0.04; 95% CI: –0.16–0.08; p=0.49), quality of life (relative risk: –0.97; 95% CI: –18.63–16.70; p=0.91), and duration of surgery (relative risk: –3.87; 95% CI: –9.26–1.53; p=0.16). The authors consider TOTO as beneficial with regard to postoperative pain, duration of surgery, and postoperative bleeding rate. Regarding recurrent symptoms of obstruction, quality of life, and immune function, TOTO was equal to TE. With regard to the long-term success of OSAS therapy, TOTO was inferior to TE.

The meta-analysis, however, does not clearly explain what the only disadvantage of long-term poorer success exactly means. It can only be assumed that it refers to SBD recurrence. Therefore, a closer look to those publications with a follow-up of least one year is advisable. We identified studies with a follow-up of 12 months [108], [115], on average 16 (6–24) months [111], on average 64.3 (60–72) months [117], and 72 months [109].

For clarification, this aspect from the publications is explicitly described here:

- Hultcrantz [108]: after 6 months 2/22 children snored again and still after 12 months, even with lower intensity. One of the children was severely overweight, another one had tonsillar regrowth (9.1%).
- Hultcrantz [109]: none of the children snored 6 months after TOTO (n=21), but 2 of them after 1 year. 6 years after TE, 4/20 and after TOTO 8/21 snored again, however with lower intensity. 14/20 after TE and 11/21 after TOTO did not snore any more 6 years after the intervention (20%).
- Reichel [111]: 2/49 children underwent TE because of airway obstruction caused by recurrent tonsillar hyperplasia (4.1%).
- Morinière [115]: 3/88 children developed tonsillar hyperplasia after an average of 30.2 (15–48) months, 4/5 were younger than 5 years. Only 2/5 children underwent revision surgery (TE) (2.4%).

**Conclusion drawn by the authors: **Tonsillotomy may be advantageous over tonsillectomy in the short term measures and there are no significant difference of resolving obstructive symptoms, quality of life and postoperative immune function. For the long run, the dominance of tonsillotomy may be less than tonsillectomy with regard to the rate of sleep-disordered breathing recurrence.

##### 3.2.3.3.2 assessed by Marcus et al.

This publication [118], [119] is an update of a guideline that was initially written in 2002 [90]. The paper focussed on the diagnosis, therapy, and follow-up of children (>1 year) with OSAS, exclusively in cases of adeno-tonsillar hyperplasia with/without obesity (body mass index >95.percentile, adapted for age/gender) without concomitant morbidities. The literature review was limited to the period from 1999 to 2008. Regarding the patients’ history, the authors recommended to systematically evaluate the symptoms (see 3.2.3.1) in order to indicate PSG (preferably) or alternative procedures such as pulse oximetry, nighttime videos, outpatient somnography (in exceptional cases) in time. If adenotonsillar hyperplasia is confirmed, ATE is the therapy of choice. The co-factor of obesity limits the surgical outcome but this aspect cannot be predicted in the individual case. The single measure of ATE is clearly preferred in contrast to permanent therapy with CPAP mask. Risk factors such as an age of less than 3 years, secondary cardiac diseases, obesity, craniofacial anomalies, neuromuscular diseases, and respiratory infection are mentioned. Since the criteria of severe alterations in the PSG depend on the age and gender and since there are no high-quality trials on this topic, the committee considers as severe: maximal oxygen desaturation <80% or AHI ≥24/h or hypercapnia (pCO2≥60 mmHg) in the PSG (preoperatively or in the wake-up room). In the Technical Report belonging to this article, the references are listed [119], among others also for TOTO and TE. After very short summaries of some trials [120], [121], [122], [123], [124], [125], [126], [127], [128], the authors identified a lack of high-quality studies comparing the effect of TOTO vs. TE on OSAS. The risk of adeno-tonsillar regrowth is estimated to range between 0.5% and 16%, depending on the study design. Based on the existing trials, the authors cannot give a recommendation for a particular surgical TOTO technique. Because of the remaining risk due to the residual tonsillar tissue to develop recurrent hyperplasia or tonsillitis, regular follow-up visits are recommended. An extensive review article on the different types of partial TE was published in 2014 [128]. A table listing further trials shows the heterogeneity (percentage of obese and syndromic patients, severity of OSAS) and a high percentage of respiratory complications that become mainly obvious by a low oxygen saturation SpO_2_<80% and an AHI >24/h [93], [95], [96], [97], [129], [130], [131], [132], [133], [134], [135], [136], [137], [138], [139], [140], [141], [142], [143], [144].

**Conclusion drawn by the authors:** ATE remains therapy of choice for adeno-tonsillar hyperplasia and signs of upper airway obstruction.

##### 3.2.3.3.3 assessed by Stuck et al.

This publication [145] is also an update of an S1 guideline [146], [147] now published as S2k guideline limited to adult patients. In this context, TE is only considered as part of uvulo-palato-pharyngo-plasty (UPPP) if tonsils are present. Less invasive methods and a very strict indication of TE are mentioned, which, however, is not specified in the guideline. “Because of the relatively high morbidity, the indication of TE and uvulo-palato-pharyngo-plasty (UPPP) should be made cautiously” is repeated in the updated guideline [146]. Two retrospective trials are mentioned that could confirm a reduction of the therapeutic success in the further course, in one of the studies the intervention was only successful in every second patient after 2 years [148], in the other one, the reduction of the therapeutic effect mainly manifested in the second half of the first postoperative year. The survey was carried out after an average of 44 months (16–75) after the intervention [149].

**Conclusion drawn by the authors:** Regarding TE as part of UPPP, a very strict indication is advisable.

##### 3.2.3.3.4 assessed by Jeyakumar et al.

This systematic research of the literature [150] dealt with the correlation between SBD and enuresis as well as the efficacy of ATE. Primary (continence at no time) or secondary enuresis (continence already present for at least 6 months) may result from insufficient physical/psychical maturation, genetic factors, functional bladder disorders, and altered vasopressin secretion. In 5–10% of the pediatric cases, also SBD may be the origin. The authors included only English literature published between 1998 to 2010 and young patients (≤19 years of age) with SBD accompanied by enuresis, treated with ATE. From 39 identified trials, 14 matched the search criteria encompassing 3680 patients aged 18 months to 19 years, 52% of whom were male [94], [151], [152], [153], [154], [155], [156], [157], [158], [159], [160], [161], [162], [163]. Two studies had to be excluded leaving the data of 3550 patients with simultaneous enuresis in 1113 cases (33%) for further evaluation [94], [153], [154], [155], [156], [157], [158], [159], [160], [161], [162], [163]. In 7 trials with 1274 patients (2 to 18 years) with SDB and simultaneous enuresis in 427 cases, 587 were followed after ATE. Postoperatively, only 97 of the original 427 patients with SBD and simultaneous enuresis (22.7%) were identified. The only study with a controlled design and randomization [153] investigated 257 children in the intervention group (33% with enuresis) and 69 in the control group (35% with enuresis). After 6 months, the difference of the healing rates with 50% vs. 48%, respectively, was not statistically significant.

**Conclusion drawn by the authors:** There is a significant coincidence of enuresis and SBD in children. For therapy, ATE turned out to be effective in a large percentage of patients. Further studies are required to clarify, whether the data are biased by spontaneous remission or other factors. High-quality trials have to consider precise diagnostics of SBD by PSG and a differentiation between primary and secondary enuresis. 

##### 3.2.3.3.5 assessed by Friedman et al.

This meta-analysis [98] included English trials with young patients (<20 years of age) with an OSAS diagnosed by preoperative PSG who were scheduled for postoperative PSG. Patients with obesity were included whereas patients with chromosomal, craniofacial, or neuromuscular diseases were excluded. From 100 hits, the authors filtered 23 articles that were assessed in detail after subgroup classification regarding the surgical success (postoperative AHI<1; AHI<5; special constellations) [93], [94], [97], [100], [120], [134], [136], [137], [138], [141], [142], [143], [164], [165], [166], [167], [168], [169], [170], [171], [172]. Only 6 of those 23 articles did not reach level 2b of evidence-based medicine [94], [138], [165], [166], [167], [171]. The diagnostic criterion of OSAS was AHI <1 (9), AHI <2 (2), AHI <5 (11), or it was not specified (2). The cohort sizes varied between 10 and 199 patients with an average age of 6.5 years (2.2 to 9.3 years; age was not stated in one publication) with a percentage of male patients between 48% and 86% (gender not stated in 2 publications). According to the authors of the respective studies, the success rate varied between 24.2% and 100%. From the 23 studies, Friedman et al. calculated an overall success rate of 66.3% (95% CI: 57.5–74.1%; p<0.0001). If an AHI <1 was considered as criterion of success, only 59.8% of all patients were successfully treated (95% CI: 43.6–74%; p=0.234). In analogy, the success rate changed to 66.2% (95% CI: 54.5–76.3%; p=0.007) based on an AHI cut-off of <5. Evaluating all 23 studies, the preoperative AHI was 18.6 (6.9–69.3) and the postoperative AHI was 4.9 (0.39–14.2). The average reduction of the AHI was 12.4 (95% CI: 10.7–14.2).

Nine studies included 340 patients with particular constellations, among those were an AHI >30 [144], age <3 [137], [142], and obesity [93], [134], [136], [138], [143], [171]. The success rate of 38.7% was significantly smaller compared to 867 children without comorbidities (73.8%; p<0.0001). The average reduction of the AHI by 22.0, however, was significantly better compared to the pediatric patients without comorbidity factors (11.7; p<0.0001). It should be emphasized, that publication bias, i.e. preferred publication of studies with positive/significant results, can not be excluded.

**Conclusion drawn by the authors:** ATE can be seen as therapy of choice for treatment of OSAS based on adeno-tonsillar hyperplasia. However, surgery is successful in only 2/3 on average. If comorbidities occur, especially obesity, the success rate decreases to less than 50%. Regarding prognosis, the cut-off values of AHI and BMI are crucial. Further trials concerning concepts for therapy failure are necessary.

##### 3.2.3.3.6 assessed by Costa and Mitchell [130]

Between 2003 and 2004, 17.1% of the children and adolescents in the USA were overweight. Compared to 1999 and 2000, a significant increase of the percentage of obese children was observed, of girls (from 13.8% to 16%) as well as of boys (of 14.0% to 18.2%) [173]. Between 2011 and 2012, at least some children and infants (8.1%) and children and adolescents (16.9%; age 2–19 years) were obese. So there was no difference, but the percentage of the children between 2 and 5 years was reduced (from 13.9% to 8.4%; p=0.03) [174]. 27% of asymptomatic children and 25–40% of symptomatic children had OSAS [175], [176]. Since obesity is a prognostically relevant factor for the efficacy of ATE on OSAS, Costa and Mitchell used the following filters: age ≤18 years, BMI >95.percentile, ATE, pre- and postoperative PSG, and no neurological or craniofacial comorbidities, respectively [130]. From 100 publications, 4 matched the search criteria, 3 with prospective [134], [136], [143] and 1 [138] with a retrospective study design. In total, 110 children had undergone surgery, 18 to 33 per trial. The average age was 8.4 years (7.3–9.3 years), the average BMI was 29.7 (28.3–32.1; not mentioned by O’Brian [134]). On average, the preoperative BMI was near the 99.75.percentile. In 3 studies, PSG was performed on average 4.8 months after surgery. The AHI was reduced from 29.4 (22.2–34.3) to 10.3 (6–12.2), the average reduction of the AHI was 18.3 (95% CI: 11.2–25.5; p<0.00001). Preoperatively, the oxygen saturation was 78.4% compared to 85.7% postoperatively (83.6–89.9%, the average gain was 6.3% (95% CI: 3.9–8.7). Since AHI <5 was considered as success in 3 publications, the success rate in those publications [134], [138], [143] was higher (39–46%) compared to the children examined by Mitchell and Kelly [136] with an AHI <2 (24%) as criterion of success. This is why Costa and Mitchell used the raw data of 81 children and calculated the rate of the postoperative AHI values of <5 (49%), <2 (25%), and <1 (12%). For therapy failure, treatments such as cPAP-masks and weight loss were discussed, for assessment of the success rate of UVPPP, studies have not been conceived so far.

**Conclusion drawn by the authors: **ATE may improve the quality of sleep in obese children, but it is successful only in 12% of the cases with success defined as AHI <1.

##### 3.2.3.3.7 assessed by Brietzke and Gallagher

This meta-analysis [95] included exclusively studies in Englsih language with pre- and postoperative PSG. The authors excluded studies with patients aged 18 years or older and those with comorbidities. It was a precondition that the difference between pre- and postoperative AHI could be calculated based on the study data as well as the success rate (AHI <1; AHI <5). From 55 hits of the literature research, the authors filtered 14 case series (evidence level 4) with an average of 28 (2–114) patients with a mean age of 4.9 years [94], [100], [140], [141], [164], [165], [166], [167], [168], [177], [178], [179], [180], [181]. The postoperative PSG was performed after a mean time of 98 days. The preoperative PSG revealed an average AHI of 16.8 (6.375–26.9), and 2.42 after surgery (0.3–7.5). Applying the random-effects model, a reduction of the AHI of 13.92 on average (95% CI: 10.05–17.79; p<0.001) could be calculated. The success rate was 82.9% (95% CI: 76.2–89.5%; p<0.001). The heterogeneity of the retrieved articles becomes obvious considering the fact that the success criterion was variably defined (AHI of 0.5 to 5). Other statistical calculations to reduce the effect of small populations on the result showed a highly significant positive effect of ATE on OSA (Hedge’s G parameter: 1.43; 95% CI: 1.25–1.60; p<0.001). A publication bias was excluded.

**Conclusion drawn by the authors:** Despite the heterogenic study situation, ATE turned out to be successful in most of the cases. There is a great need of research for the development of treatment concepts for therapy failure.

##### 3.2.3.3.8 assessed by Lipton and Gozal

The search strategy is poorly described in this literature review [182] with an own evaluation system. The authors differentiated between blinded-controlled (A; 20 points), prospective (B; 10 points) and retrospective (C; 5 points) observational studies, and case reports/expert opinions (D; 1 point). 0.1 point was added to the score of each study for each subject in a given study. If the diagnosis was based on clinical history and physical examination, the overall score was multiplied by 0.5, if a single or multiple channel home recording was conducted, the score was multiplied by 0.75, and in cases of conducted PSG, it was multiplied by 1. If the outcome was based on parental report, the study score was multiplied by 0.5, if a reduced overnight study was conducted, the score was multiplied by 0.75, and if PSG was used for determination, it was multiplied by 1.

In total, the authors identified 21 trials but only 20 are found in a table that lists the studies with a score of 13.2 to 2.3) (A: 0; B: 14; C: 6; D: 0) [99], [100], [108], [164], [165], [166], [167], [168], [178], [183], [184], [185], [186], [187], [188], [189], [190], [191], [192], [193]. Further calculations (CI, significance level) are not provided. Prospective randomized trials were not found among the studies and the authors were not able to confirm the superiority of one surgical procedure in comparison to others (ATE vs. AT vs. TE). In addition, the authors stressed, that results were presumably biased by different factors (severity of OSAS; ethnics; obesity; family history; population sizes). Furthermore, they emphasize, that postoperative PSG is mandatory identify therapeutic failure and treat those patients in an alternative way. From 11 trials with instrument-based diagnostics in 401 patients after surgery, the authors were able to calculate a success rate of 80%. This percentage is in contrast to a success rate of 97% in 251 patients based on a clinical assessment. 

**Conclusion drawn by the authors:** ATE as therapy of choice for pediatric OSAS is associated with the risk of therapeutic failure. Further research is required for better selection criteria for indication as well as for the transferability of studies conducted in specialized departments to general hospitals.

##### 3.2.3.4 Table and conclusion on the indication of SBD

See Table 4 (Tab. 4).

**Conclusion:** TE performed as ATE is appropriate for treatment of SBD in pediatric patients.

#### 3.2.4 Psoriasis

##### 3.2.4.1 Practice essentials of psoriasis guttata

The first description of a correlation between acute streptococcal infection and psoriasis was made by the English dermatologist Winfield in 1916. The acute exacerbation is characterized by light red or red flat papules and plaques measuring 0.1–1.5 cm. Mostly commonly affected are children and adolescents 1–2 weeks after pharyngeal streptococcal infection. As origin, streptogenic antigens with similar structures of keratinocyte proteins are discussed [194], [195], [196], [197], [198], [199], [200], [201], [202], [203]. More rarely, this clinical variation of psoriasis develops after viral infection or vaccination. Most often, the patients reveal a primary manifestation of psoriasis, but sometimes also as episodic activity of an originally stationary chronic plaque psoriasis. The acute skin exacerbations are then found in addition to preexisting psoriasis plaques. The association with the high-risk PSORS1 gene (HLA-Cw6) is described, which is located on the 6p21.3 gene. 

The efflorescences are typically localized on the trunk and the extremities, rarely the face. The scalp may be affected as well. The name of the disease alludes to the drop-like shape of the lesions like under a shower. Psoriasis guttata responds well to topical steroids of cortisone classes 2–3. Additional phototherapy with small spectrum UVB light (311 nm) shows good results, however, it is contraindicated in children. TE is recommended when a clear correlation between the episodes of psoriasis and tonsillitis is confirmed. In cases of first manifestation in children, the prognosis is favorable regarding spontaneous healing or healing with sufficient local therapy within a few weeks. In 30–70% of the cases, the course becomes chronic [204], [205].

##### 3.2.4.2 Assessment by the Cochrane Collaboration 

For therapy of psoriasis guttata, 2 meta-analyses of the Cochrane Collaboration [34], [39] and 1 systematic research of the literature by the Cochrane Collaboration Skin Group [206] were found. Both meta-analyses were published in 2000, the same group published a systematic literature review in 2001 without additional information. Chalmers et al. published the last update without changing the original version [39] in 2015, and the original version of Owen et al. [34] was published in 2013. All three publications were elaborated by the same authors. In the meta-analysis by Chalmers et al., prospective RCTs were not obtainable from the literature comparing the different treatment modalities of psoriasis guttata. The authors found only one single study with 21 adult patients who were treated with fatty acid infusions. TE was not the subject of this meta-analyses [39]. The last update of July 17, 2013, announces a new version with a new team of reviewers (author’s note: psoriasis is mostly observed in children and regresses after removal of the triggering factor, therefore prospective trials are not retrievable from the literature).

**Conclusion drawn by the authors:** data are insufficient to prescribe antibiotics or indicate TE as a regular treatment modality.

##### 3.2.4.2.1 assessed in the review of the Cochrane Collaboration [34, 206]

The correlation of streptococci-associated pharyngeal infection and psoriasis vulgaris episodes (author’s note: not psoriasis guttata) suggests antibiotic therapy. Probably super-antigens on the cell membranes of the bacteria cannot be detected by the immune system and at the same time trigger a malfunction that is directed against own keratinocytes. Also TE is performed repeatedly in order to remove the typical location of confrontation with streptococci. Historical publications in this context are:

- the retrospective trial by Nyfors who followed 74 patients for 4.5 years on average. He observed remission in one third of the patients, another third had improved symptoms, even without tonsillitis episodes in their history [207], [208]
- Rosenberg reported healing of psoriasis after TE in 9 of 14 patients with confirmed streptococcal infection [209]
- Hone reported 6 patients with psoriasis guttata and 7 patients with chronic psoriasis, acutely exacerbating by tonsillitis. In 5 of 6 and 2 of 7 patients, respectively, TE resulted in a remission, in 1/6 and 2/7 the symptoms were at least improved, and in only 3/7 TE had no efect [210]
- McMillin reported of 2 children with recurrent streptococci-associated tonsillitis/pharyngitis and concomitant psoriasis guttata that did recur within 16 months after ATE [211]
- Ozawa in Japan scheduled 385 patients with palmoplantar psoriasis for TE and registered improvement in 16.7% of all cases, Tsubota even in 72% of 289 patients after TE [212] (author’s note: pustulosis palmoplantaris is an own disease that nowadays does no longer belong to psoriasis, however, about 25% of those patients also suffer from chronic psoriasis).

**Conclusion drawn by the authors:** There are no high-quality studies indicating a strong benefit from TE in patients with psoriasis.

##### 3.2.4.3 Assessment by other systematic reviews or meta-analyses

##### 3.2.4.3.1 assessed by Rachakonda

This systematic narrative literature review [213] included all controlled trials or observational studies in which patients with psoriasis vulgaris (author’s note: not psoriasis guttata) had undergone TE and were examined at least once after surgery. From 674 hits, 20 studies were eligible for further analysis, including one RCT [214], one retrospective study [208], 4 prospective observational studies [215], [216], [217], [218], 7 case reports [200], [211], [219], [220], [221], [222], [223] (<5 patients per study), and 7 case series [209], [210], [224], [225], [226], [227], [228] (≥5 patients per study). 12 articles were published before 1980, 8 since 1994. Because of the heterogenic quality of the 545 patient data and individually unprecise definitions, a meta-analysis was not possible. However, in 290 of 410 patients, the intervention resulted in an improved outcome, the follow-up period varied between 2 months and 10 years. In some cases, improvement meant a longer psoriasis-free time after TE comparison to the episodes before the intervention [208] or the patients showed better response rates to pharmaceutics after TE [217].

The only RCT revealed a significantly (p<0.01) better course in the intervention group (n=15; mean age: 35.3 years) in comparison to the control group (n=14; mean age: 35.9 years).

**Conclusion drawn by the authors:** Tonsillectomy may be a potential option for patients with recalcitrant psoriasis associated with episodes of tonsillitis. Studies with long-term follow-up are warranted to determine more clearly the extent and persistence of benefit of tonsillectomy in psoriasis.

##### 3.2.4.4 Table and conclusion on the indication of psoriasis

See Table 5 (Tab. 5).

**Conclusion:** TE is probably appropriate for treatment of psoriasis guttata.

#### 3.2.5 Periodic fever, aphthous stomatitis, pharyngitis, and adenitis syndrome (PFAPA syndrome)

##### 3.2.5.1 Practice essentials of PFAPA syndrome

The first description of this disease in 1987 and the introduction of the acronym of PFAPA two years later was made by Marshall [229], [230]. It is acknowledged as the most common non-hereditary fever syndrome of unknown origin, presumably resulting from immunologic dysregulation [231], [232], [233]. The syndrome typically develops before the age of 5 years and is characterized by symptomatic intervals of 30 (14–50) days with fever recurrences (>39°C) for 4 (2–7) days with simultaneous aphthous stomatitis, pharyngitis, and/or cervical lymphadenopathy [234]. Sometimes also splenomegaly is diagnosed. Often headaches and stomach pain, more rarely also joint pain, are observed. Between the fever episodes, the children are asymptomatic. The diagnosis requires the exclusion of infectious, immunologic, and malignant disease as well as hereditary periodic fever syndromes. The interpretation of the clinical signs and the typical history play a key role [230], [233]. The treatment is primarily performed based on the symptoms by administration of non-steroidal analgesics. Prednisone is also applied and is effective within 12 to 24 hours, however, it sometimes results in a reduction of the fever-free intervals [233], [235], [236], [237]. For one third of the patients, success was reported with a 6 to 12 month cimetidine prophylaxis (20 mg/kg/d) [238], also ATE turned out to be effective [239], [240], [241]-. According to Feder et al. the syndrome disappears spontaneously in the course of an average of 33 months (8–92 months, median: 24 months) without causing permanent damage [234].

##### 3.2.5.2 Assessment by the Cochrane Collaboration [32]

The meta-analysis from 2014 [32] is an update of the first version from 2010 [242]. All RCTs published until October 2013 were included to analyze the efficacy of TE in comparison to non-surgical procedures. In both meta-analyses two of 192 studies matched the search criteria. Both articles encompassed a total of 67 children aged between 1.5 and 14 years [243], [244]. The primary endpoints were the success rate and surgical complications, secondary endpoints were the number and the severity of episodes, corticoid application, absences from school as well as the quality of life. 

In the study by Renko et al. the children had a mean age of 4.1 years and had an average of 9.3 (4–20) episodes per year [244]. In the study by Garavello et al., the children had a mean age of 5.1 years and had 8.7 (4–12) episodes per year [243]. The diagnosis in Renko’s article was made less strictly and included patients with ≥5 attacks, which generally meant fever of >38.5°C of unclear origin recurring in intervals of 2–5 weeks. Other associated symptoms were not regularly present which led to uncertain diagnoses and lacking differentiation of streptococci-associated tonsillitis that was made exclusively based on clinical criteria. The pediatric patients of this study underwent TE. Garavello et al. included children when they were less than 5 years old and developed acute fever for about 5 days and had at least simultaneous aphthous stomatitis (61% of the intervention group vs. 58% of the control group) or pharyngitis (98% vs. 97%) or cervical lymph node disease (89% vs. 82%). An additional criterion was the spontaneous remission after corticoid application and symptom-free intervals as well as regular development. The children of the intervention group of this trial underwent ATE. For the control group in both studies it remains unclear whether or not they received cimetidine as prophylaxis. Renko et al. treated symptomatically without and Garavello et al. with corticoids. The follow-up period was 12 months with evaluation after 6 [244] and 18 months [243]. A criterion for success was the disappearing of fever episodes 6 months after randomization, which was achieved in 10 of 14 operated children and in 4 of 12 non-operated children (lost to follow-up: 2) [244]. Garavello et al. considered success as the immediate symptom-free condition for more than 18 months, which was observed in 12/19 operated and in 1/20 non-operated children (no child lost to follow-up) [243]. Regarding the difference of the follow-up period, it remains unclear if an evaluation already after 6 months in the trial of Garavello et al. may have resulted in a higher success rate, and, if between the 6^th^ and 18^th^ month after randomization in the study of Renko et al. recurrences may have had occurred. Feder et al. reported a spontaneous remission rate of 20% when follow-up was made for a mean time of 33.2 (8–92) months [234], Thomas et al. registered a rate of 41% within 4.5 years [233]. All operated children in both studies were symptom-free until the end of the follow-up period, no data are provided for the control group of Garavello et al. Regarding the success rate, a relative risk was calculated for all patients from both trials of 4.38 (95% CI: 0.64–30.11), an odds ratio of 11.04 (95% CI: 1.77–69.08), and a risk difference of 0.52 (95% CI: 0.32–0.72). The heterogeneity of the data is reduced to an acceptable measure only for the risk difference. The number-needed-to-treat benefit was given with 2 which corresponds to an absolute risk reduction of 50%. Regarding complications, absences from school, number of cortisone applications, and quality of life, no data were obtainable from the studies. The number of fever attacks and associated symptoms per month per patient was 0.05 in the intervention group and 0.47 in the control group of Renko et al., which corresponds to a relative risk of 0.1 (95% CI: 0.04–0.28). Garavello et al. calculated the average number of episodes for the 18 month follow-up ± standard deviation which was 0.7±1.2 for the intervention group and 8.1±3.9 for the control group, respectively. This difference of 7.4 (95% CI: –9.19–5.61) was statistically significant. The summary of the data reveals a rate ratio of 0.08 (95% CI: 0.05–0.13) corresponding to a 92% reduction of the monthly episodes per patient by surgery, which is equal to 1.1 instead of 31.3 disease days within 18 months (0.06 instead of 1.7 days per month). Only Garavello et al. counted the disease duration per episode after randomization: to 1.7 (2–4) days in the intervention group vs. 3.5 (2–5) days in the control group. Regarding cortisone application, Renko et al. give no information and Garavello et al. give only unprecise data so that 53% of the intervention group and 90% of the control group (relative risk: 0.58; 95% CI: 0.37–0.92) were calculated with great caution. Heterogeneity index I^2^ was found to be 71% concerning complete remission and 0% in relation to the number of fever episodes and symptoms.

**Conclusion drawn by the authors:** The evidence for the effectiveness of tonsillectomy in children with PFAPA syndrome is derived from two small randomised controlled trials. These trials reported significant beneficial effects of surgery compared to no surgery on immediate and complete symptom resolution (number needed to treat to benefit = 2) and a substantial reduction in the frequency and severity (length of episode) of any further symptoms experienced. However, the evidence is of moderate quality (further research is likely to have an important impact on our confidence in the estimate of effect and may change the estimate) due to the relatively small sample sizes of the studies and some concerns about the applicability of the results. Therefore, the parents and carers of children with PFAPA syndrome must weigh the risks and consequences of surgery against the alternative of using medications. It is well established that children with PFAPA syndrome recover spontaneously and medication can be administered to try and reduce the severity of individual episodes. It is uncertain whether adenoidectomy combined with tonsillectomy adds any additional benefit to tonsillectomy alone.

##### 3.2.5.2.1 assessed in the review article of the Cochrane Collaboration: Garavello et al. [243]

The computer-based randomization of pediatric patients (<5 years of age) with PFAPA syndrome was conducted between February 2003 and December 2006 [243]. The recurrent abrupt fever episodes had to develop from complete healthy condition and last for approximately 5 days. At least one of the following symptoms had to occur simultaneously: aphthous stomatitis, pharyngitis, or cervical lymph node swelling without additional signs of airway infection. Furthermore, the fever had to respond directly to corticoid application and disappear. Between the fever episodes, symptom-free condition and regular development were required. Patients with cyclic neutropenia and other autoinflammatory syndromes (hereditary Mediterranean fever, hyperimmunoglobulinemia D syndrome, Behçet’s disease) as well as clinical and chemical proof of immune deficiency, autoimmune disease, or chronic infection were excluded. The children either underwent ATE or conservative therapy and were regularly followed-up in 3 months intervals for 18 months or contacted by telephone. Parents and physicians regularly documented the body temperature, in both therapy arms cortisone was allowed. Primary endpoint of the study was the success rate permanently within the 18-month follow-up period. Secondary endpoint was the remission of the symptoms in the course. According to statistical calculation, 40 patients (20 per therapy arm) patients were required.

19 patients were allocated to the intervention group (ATE) vs. 20 of the control group (wait-and-see) with a percentage of male patients of 47 vs. 35%, respectively. The mean age of the patients was 2.9 (2.2–4.1) vs. 3.1 (2.4–4.4) years, respectively, the number of episodes per year was 8.6 (4–12) vs. 8.8 (5–12) with an average duration of 3.3 (2–4) vs. 3.5 (2–5) days and a maximum temperature of 39.6 (38.7–40.6) vs. 39.8 (38.9–41.0) °C, respectively. Pharyngitis was observed in nearly every child (98 vs. 97%), more rarely cervical lymph node swellings (89 vs. 82%), and even more rarely aphthous stomatitis (61 vs. 58%) occurred.

Postoperative complications did not occur. A rapid and complete remission was achieved in 13 patients, 12 of them had undergone surgery. The significantly different success rate was 63% vs. 5% (p<0.001). The only child with complete remission in the control group was a 9-year-old girl with PFAPA syndrome persisting for 4 years who presented in the year before randomization every 2 months for an average of 2.5 (2–3) days with a maximum body temperature of an average of 39.3°C (38.9–39.6°C). During 2 episodes she had received cortisone. In the intervention group, no clinical signs were found that were relevant for prognosis. In the ATE group, the average number of episodes (± standard deviation) was 0.7±1.2 vs. 8.1 ±3.9 in the control group (p<0.001). The percentage of children with less than 3 episodes during the 18 months of follow-up was significantly higher than in the control group. Recurrences were observed after ATE mainly during the first 6 months after surgery: after the first postoperative year, no recurrences had developed.

**Conclusion drawn by the authors:** PFAPA syndrome can be treated effectively with ATE.

##### 3.2.5.2.2 assessed in the review article of the Cochrane Collaboration: Renko et al. [244]

The data of this multicenter trial from Finland were collected between 1999 and 2003 [244]. It included children with at least 5 regularly occurring fever episodes of unclear origin (temperature ≥38.5°C) and symptom-free intervals of 2 to 5 weeks. Additionally, aphthous stomatitis, pharyngitis, and cervical lymph node swelling were documented. Before participating in the study, the selected 28 children had an average of 9 fever episodes (4–20) for 3.6 (2–6) days on average and symptom-free intervals of 25.6 (18–28) days. In 41%, fever was the only symptom, 29% had at least once concomitant tonsillitis, and 21% either had cervical lymph node disease, aphthous stomatitis, or pain in mouth or throat. The collective also encompassed 1 pair of twins. Another pair of twins whose parents refuted participating in the study, was symptom-free after TE. One child was lost to follow-up after TE. Therefore, the data of only 26 children were evaluated. In the control group, acute leukemia prevented participation in one child.. In the intervention group, the patients exclusively underwent TE, the therapeutic concept for the control group was not described. The groups encompassed 14 vs. 12 patients, 57% vs. 67% were male with an average age of 4.2 (1.5–14) vs. 4.0 (1.5–7.2) years. Fever lasted for 9.0 (5–20) vs. 9.5 (4–20) days on average with a median value of 3.4 (2–4) vs. 3.8 (2.5–6) days. The symptom-free interval was 25.9 (21–28) vs. 25.0 (18–28) days on average. 7/14 vs. 4/12 children reported only recurrent episodes of fever. After randomization (method unclear: “balanced randomization was used at each center to minimize bias”), TE was performed within 1 months or the patients were observed. The symptoms had to be documented in a diary, an evaluation was scheduled after 6 months. If the symptoms persisted in the control group, the children were scheduled for TE. Primary endpoint of the study was the remission of the fever episodes at the follow-up examination. All children having undergone TE and 6/12 children from the control group were symptom-free after 6 months which represents a highly significant difference (CI 23%–75%; p<0.001). In the control group 5/6 of the therapeutic failures underwent successful TE, as confirmed after further 6 months. Postoperative complications did not occur. One child with persisting symptoms from the control group did not undergo TE due to the parents’ wish because the severity of the symptoms had decreased. 4 of the 14 children from the intervention group had a fever episode during the first 6 months after surgery (0.005 monthly episodes per child), in the control group 34 episodes were registered in 12 children (0.47 monthly episodes per child). This difference is statistically significant (risk difference: 0.40, 95% CI: 0.17–0.62; p<0.007). The accompanying symptoms had no effect on the outcome. Failure of therapy was not associated with any clinical sign.

**Conclusion drawn by the authors:** Tonsillectomy appeared to be effective for treating PFAPA syndrome. The fever episodes ceased without any intervention in half of the control subjects. We conclude that although the mechanisms behind this syndrome are unknown, tonsillectomy can be offered as an effective intervention for children with PFAPA.

##### 3.2.5.3 Assessment by other systematic reviews or meta-analyses

##### 3.2.5.3.1 assessed by Garavello et al. [245]

The authors reviewed the English literature from January 1987 to May 2010 and selected 15 from initially 33 publications [245], most of them were retrospective [233], [234], [237], [239], [240], [241], [246], [247], [248], [249], [250] or prospective [236], [251] case series; 2 studies had a randomized controlled design [243], [244]. The sizes of the collectives varied between 2 and 27 patients. In 2 trials, diagnosis was established by unclear criteria [241], [244]. Follow-up periods (1–118) months and the type of intervention (exclusively TE (8), exclusively ATE (3), ATE and TE (4)) also varied considerably. According to the varying definition of success, rates ranged between 0% and 100%, the pooled success rate is 83% (95% CI: 77–89%). In 124 of 149 cases, surgery was successful, for definition of treatment failure, however, no sufficient data were obtainable. Only 2 case reports with 2 [248] and 5 [247] patients reported failure [248] or only slight improvement [247].

**Conclusion drawn by the authors:** Surgery appears to be a possible option for management of PFAPA syndrome. Available evidence is limited, however, and the precise role of surgery remains to be clarified. We suggest considering this option when symptoms markedly interfere with the child's quality of life and medical treatment has failed.

##### 3.2.5.3.2 assessed by Peridis et al.

This meta-analysis [252] included all trials published between 1987 and 2010, analyzing the outcome of conservative therapy vs. TE/ATE. From 64 studies, the authors filtered 1 randomized [243], 1 prospective [251], and 12 retrospective trials [233], [234], [235], [236], [237], [239], [246], [247], [248], [249], [250], [253]. The quality was assessed according to the Newcastle-Ottawa scale regarding selection, comparability of the groups, and evaluation of the results. In total, the studies encompassed 374 children who were treated with surgery (n=124; 33.15%), antibiotics (n=143; 38.24%), cimetidine (n=70; 18.72%), and cortisone (n=257; 68.72%). The collective size varied between 1 and 105 patients, the follow-up period was between 1 and 120 months. Apart from fever that was always measured in the affected children, simultaneous pharyngitis (84.18%), lymphadenitis (72.88%), aphthous stomatitis (5.199%), skin exanthema (10.74%, nasal obstruction (33.89%), headaches (46.13%, stomach pain (45%), joint pain (23.64%, and diarrhea (2.5%) were observed.

The meta-analysis revealed that:

- antibiotics were ineffective (odds ratio: 0.01; 95% CI: 0.00–0.01; p<0.00001).
- prophylaxis with cimetidine was ineffective (odds ratio: 0.15; 95% CI: 0.03–0.75; p=0.02). Cortisone was effective (odds ratio: 43.82; 95% CI: 10.68–179.69; p<0.00001).
- TE/ATE were effective therapies (odds ratio: 27.26; 95% CI: 6.7–110.91; p<0.00001).
- TE/ATE were superior to cimetidine (odds ratio: 11.89; 95% CI: 2.36–60.02; p≤0.0003).
- TE/ATE were superior to antibiotics (odds ratio: 106.49; 95% CI: 30.289–374.44; p<0.00001).
- TE/ATE were as effective as cortisone (odds ratio: 0.9; 95% CI: 0.36–2.26; p=0.83).

Those statements were also applicable for studies with at least 8 patients, publications after 2005 as well as studies of high quality according to the Newcastle-Ottawa scale.

**Conclusion drawn by the authors:** The most effective non-surgical therapy is corticosteroids. However, they do not prevent future fever cycles. The results of this meta-analysis showed that tonsillectomy (+/- adenoidectomy) is the most effective intervention for long-term resolution of PFAPA syndrome symptoms.

##### 3.2.5.3.3 assessed by Leong et al. [254]

The search strategy of this literature research [254] is described imprecisely; 27 publications were mentioned, 20 of them were published in English language and published since 1989. Among them, 5 single case reports are identified, 2 case reports with 2 patients each, and 6 retrospective studies which were presented in 3 tables with clinical and demographic data [233], [239], [246], [249], [250]. Tasher et al. reported the youngest patient (1 month) suffering from PFAPA syndrome. Also in this context, the predominance of the male gender is registered with an onset of the disease before the age of 5 years (1.9–4.2 years), duration of the episodes ranging between 2 to15 days (average 4.3–5.3 days), and symptom-free intervals of varying length (25.9 to 35.7 days). The authors emphasize the following characteristics to distinguish PFAPA from other diseases:

- first manifestation before the age of 5 years
- aphthous stomatitis, pharyngitis, cervical lymph node swelling
- exclusion of cyclic neutropenia
- complete symptom-free intervals between the fever episodes
- regular physical/psychical development

The aforementioned limitations in retrospective studies are worth to be repeated: possibility of spontaneous healing, imprecise diagnosis, successful therapy with cortisone, insufficient duration of follow-up, variable results (success rate of 0–100%), small patient populations (2–15 patients).

**Conclusion drawn by the authors:** PFAPA usually resolves without any long-term adverse effect, and as such, there is no role for tonsillectomy in these patients.

##### 3.2.5.4 Table and conclusion on the indication of PFAPA syndrome

See Table 6 (Tab. 6).

**Conclusion:** TE is probably appropriate for treatment of PFAPA syndrome.

#### 3.2.6 Peritonsillar abscess

##### 3.2.6.1 Practice essentials of peritonsillar abscess (PTA)

In cases of peritonsillar abscess (PTA) pus is accumulated between the tonsils and the surrounding muscles. The symptoms are commonly very different from acute tonsillitis and usually occur in patients with a specific susceptibility [255] as well as preferably in smokers [256], [257], [258], [259], [260], [261], [262], [263], [264], [265], [266], [267], [268], which, however, was not obligatorily observed [269], [270]. Infectious mononucleosis, compromised immune system, and Kawasaki’s syndrome are acknowledges as risk factors for PTA [259], [267], [271], [272], [273], [274], [275], [276], [277], [278], [279]. Key symptoms are odynophagia, trismus, muffled voice, fever, and a reduced physical state. Clinically, there is an asymmetry of the oropharynx with protrusion of the soft palate and a displacement of the uvula to the contralateral side [267]. The symptoms present in most cases very clearly. Diagnostic difficulties are infrequently occur in very young patients because of the specific anatomy and incompliance during examination. To increase the diagnostic certainty, sometimes imaging techniques were applied that did not become standard and thus remained decisions for single cases [280], [281], [282], [283], [284], [285], [286], [287]. Peritonsillitis, Plaut-Vincent angina, lymphadenitis colli, infectious mononucleosis, malignant lymphoma, foreign body, aneurysm of the internal carotid artery, or dentogenic abscess have to be included in the differential diagnosis. Especially peritonsillitis is difficult to differentiate and sometimes requires needle aspiration or incisional drainage to be distinguished. Unexperienced physicians have difficulties in this regard, in particular if the PTA is located in the retrotonsillar space, so that the findings can be interpreted also in a false-negative way [288]. According to Templer, diagnosis of PTA is based on the evidence of pus, otherwise the term of peritonsillitis should be used [289]. Bilateral PTA has been reported in rates of 0.8% [260], [266], [290], 1.0% [267], [291], 1.8% [270], 3.88% [292] and even 4.9% [293], [294], respectively.

PTA is commonly acknowledged as an infection of the connective tissue between the tonsillar capsule and the posterior wall of the tonsillar fossa is assumed starting from the tonsillar crypts. Other authors consider PTA as result of an infection of the supratonsillar Weber’s glands or remnants of the second pharyngeal pouch [295], [296], [297], [298], [299], [300]. Two observations contradict the statement that PTA is a complication of recurrent tonsillitis:

- Only 7.9 to 56% of the patients with PTA repoted recurrent episodes of tonsillitis their history [257], [260], [266], [270], [290], [291], [301], [302], [303], [304], [305], [306], [307], [308], [309], [310], [311], [312], [313], [314], [315], [316], [317], [318], [319]. This aspect is confirmed by current studies where a correlation between tonsillitis in the patients’ history and PTA was found in only about half of the PTA patients [269], [320]. Wang from Taiwan analyzed 28,836 patient records and revealing a correlation was in only 69.9% of all cases. The group of Wang calculated a 2.82-fold higher risk for the development of PTA if patients had at least 5 tonsillitis episodes within the preceding year requiring antibiotic treatment. In the context of 1 to 4 episodes, the risk was only increased by factor 1.59 [321]. Kronenberg et al. estimated a 4-fold higher risk for patients with a positive history of recurrent tonsillitis in contrast to patients without throat infections in their history (40 vs. 9.6%) [309]. Savolainen identified a cut-off value of 3 tonsillitis episodes per year as significant risk factor and registered 89.5% of the recurrences within 2 months after initial diagnosis of PTA [306].
- In 1995, Herzon emphasized that TE does not prevent PTA and quoted 3 reports [315], [322], [323] which have been confirmed recently [267], [295].

In rare cases, PTA may be followed by severe complications including arrosion of the carotid artery [324], [325], [326], [327], jugular vein thrombosis [328], mediastinitis [329], [330], sepsis [331], pericarditis [331], meningitis [332], or deep fasciitis [333], [334], [335], [336]. Infants are at risk to be dehydrated, on the one hand because of the swallowing related pain leading to a refusal to eat, on the other hand because of the fever [337]. Therapy of PTA consists of abscess drainage and empiric broad-spectrum antibiotic therapy [301], [307], [320], [338], [339], [340], [341], [342], [343].

Different surgical procedures have been described in the past, however, a gold standard still does not exist [256], [301], [304], [344], [345] for needle aspiration (NA), incision and drainage (ID), abscesstonsillectomy, also called TE *à chaud* (TAC) [256], [301], [346]. Already in 1859, Chaissaignac reported on his experiences with abscesstonsillectomy [347], later recommended by Winckler as standard therapy for PTA [348], confirmed by a first study of Virtanen in 1949 [349]. Arguments for abscesstonsillectomy include: definitive removal of the abscess focus, removal of an occult contralateral abscess, only one inpatient treatment, diagnosis of a previously undetected malign disease, avoiding painful secondary drainage, needle aspiration and treatment of retrotonsillar spreading of the abscess [345], [350]. Occult malignomas are very rare (0.3%), the diagnosis depends on histopathologic examination [351], [352], [353], [354], [355] and is therefore impossible after NA or ID. First reports of ID date back to Paulus from Aegina (625–690 A.C.) and Guy de Chauliac in 1362 [356]. This procedure avoids the disadvantages associated with abscesstonsillectomy, including the risks of abscess opening during intubation, post-tonsillectomy hemorrhage, sepsis resulting from surgery, general anesthesia. Moreover, the is no waiting time required for ID. During the last decades, the results of NA and ID were repeatedly reported, even their application in children [288]. The advantages of NA, introduced by King in 1961 [357], encompass: easy-to-learn, easy-to-perform, rapid confirmation of a clinical suspicion, minimal trauma, good tolerance, immediate relief, no surgical/anesthesiological risks, low pain intensity [301], [304], [305], [358]. However, some disadvantages have to be mentioned, such as: painful procedure, aspiration of leaking pus, potential risk of painful re-draining, and the risk of damaging the internal carotid artery [288].

##### 3.2.6.2 Assessment by the Cochrane Collaboration

No entries were found on this subject.

##### 3.2.6.3 Assessment by other systematic reviews or meta-analyses

##### 3.2.6.3.1 assessed by Powell and Wilson [359]

The authors reviewed the English literature, published between 1991 and April 2011 [359]. From 424 articles, 45 were considered as relevant and classified according to their level of evidence [360]. Different subjects of basic diagnostics, screening in (suspected) cases of infectious mononucleosis, imaging, swab tests, antibiotic and surgical therapy, inpatient and outpatient treatment options as well as TE in the interval were covered by this review but without statistical calculations [359]. Variable procedures of therapy are mentioned in the text with TE recommended with evidence grade D for patients with risk factors such as an age <40 years [309], [361], or recurrent episodes of tonsillitis in the history [306], [309], [362]. The authors concluded for:

- needle aspiration (NA): a success rate of about 90% was confirmed by 2 randomized studies, prognostic criteria were not mentioned [304], [363]
- incisional drainage (ID): the success rate of about 90% is comparable to the one of NA [304], [364]
  Grade of recommendation B: the choice between NA and ID should be made depending of the therapist’s experience and the patient’s wish.
  Grade of recommendation D: pain reduction is more rapidly achieved after ID than after NA [365].
- Abscesstonsillectomy; TE à chaud (TAC): because of the necessary general anesthesia, the risk-benefit-ratio has to be weighed, especially in case of intubation problems. Liver involvement and trismus do not contradict surgery [364], children benefit from the definitive, painless treatment under general anesthesia [366]. Bleeding complications requiring revision occur in 0–6% of the cases [367], [368], [369], [370], [371], this rate, however, is not different from elective TE [367], [369].
  Grade of recommendation B: patients with reduced compliance and children as well as patients with PTA recurrences benefit from TAC.
- Interval TE (ITE): blood loss and absence from work were higher after ITE than after TAC, there was no difference regarding the days of hospitalization [364]. In the study published by Chowdhury and Bricknell, the inpatient stay was even longer after ITE than after TAC [263]. According to Raut and Yung, at least in 2000, the majority of ENT physicians (83%) wait after one single attack of PTA before ITE is indicated. The clinical data of this survey support such a wait-and-see strategy, because even after 2 to 8 years without ITE, almost all patients were free from disease. TAC should be preferred but only if conservative measures had repeatedly failed [372].
  Grade of recommendation D: if TAC is not possible but the above-mentioned risk factors for PTA recurrence exist, ITE is justified. However, patients should not undergo a useless intervention with a significantly increased difficult dissection.

**Conclusion drawn by the authors:** Peritonsillar abscess is a common condition with increasing incidence. We demonstrate the potential for evidence-based modifications in clinical management. However, lack of national consensus may mean that this evidence base is not being adequately exploited in current practice. A national audit of peritonsillar abscess management, in particular looking at recurrence rates and patient experience with different management strategies, appears indicated.

##### 3.2.6.3.2 assessed by Johnson

In this research of the literature [346] only English clinical studies were included that were published between 1966 and 2001 and answered at least one of 3 clinical questions:

1. Does adjuvant steroid application improve the outcome of the therapy of PTA?
2. Which surgical method of abscess drainage has the highest success rate?
3. How high is the PTA recurrence rate and what are the criteria for elective prophylactic TE?

The publications were analyzed according to the Oxford Centre for Evidence-base Medicine recommendations [373]. From 517 publications, the authors filtered 42 that, however, could not give an answer to the first question. Because of the moderate quality of the identified studies, only answers with recommendation grade C could be given for questions 2 and 3. Among those 42 publications, there were only 5 level I trials [263], [304], [345], [363], [364], the remainder were classified as level IV including 13 case series with only one treatment modality [301], [302], [303], [305], [314], [343], [358], [374], [375], [376], [377], [378], [379], 12 case series comparing different therapies [261], [264], [307], [308], [366], [380], [381], [382], [383], [384], [385], [386], and one economic analysis [301]. Concerning recurrence rates and indication, the authors found 2 level II studies [309], [313] and 13 level IV trials [301], [303], [310], [311], [312], [314], [343], [350], [361], [372], [387], [388], [389]. From the 5 randomized trials, 3 compared the outcome after NA vs. ID in cohorts of 5 [304], 60 [363], and 62 patients [345] and could not reveal a significant difference. At least a success rate of 87% after NA was achieved [363]. The apparently good results were not confirmed by the statistical calculations of the reviewers. The population sizes were too small. The number-needed-to-treat is given with 48 after ID, i.e. 48 patients would have to be treated in order to avoid 1 failure after NA. Fagan et al. compared the absence from work and time of hospitalization after ITE vs. TAC and revealed that TAC significantly reduced the absences from work [364]. Chowdhury and Bricknell performed surgery depending on the availability of experienced surgeons and could confirm a significantly shorter time of hospitalization after TAC vs. ITE [263]. The remaining cases series, did not provide any statistical calculations but the effectiveness of NA, ID, and TAC. The low data quality of two other studies impeded statistical calculations and solid conclusions. While Kronenberg et al. [309] identified recurrent episodes of tonsillitis as a risk factor for PTA in 280 patients, Wolf et al. [387] could not verify this statement after analyzing the data of 19 patients that. All trials are listed in a table but not discussed in detail.

**Conclusion drawn by the authors:** Overall, grade C evidence indicates that several methods of initial surgical drainage are equally effective, and the recurrence rate is low. The literature does not specifically address different treatments for children and adults.

##### 3.2.6.3.3 assessed by Herzon [301]

Among other aspects, this publication encompasses a narrative review, erroneously entitled “meta-analysis” [301]. However, the paper does not provide any statistical calculations such as odds ratio, risk ratio, information on the level of significance, and CI. At the same time, the publication provides information about a survey of ENT physicians in the USA enquiring the treatment concepts for PTA. Furthermore, Herzon performed a calculation of the treatment costs depending on the therapeutic modalities. Finally the data of a retrospective case series of his own department with 123 patients were presented who had undergone NA for PTA. In a historic overview, Herzon discussed the following aspects of PTA:

- Abscesstonsillectomy (tonsillectomy à chaud; TAC): In 1859, TAC was first promoted by Chaissaignac [347], it was recommended as standard therapy by Winckler in 1911 [348] and Barnes in 1915 [390], a first trial was published by Virtanen in 1949 [349]. In different studies, TAC turned out to be a safe measure that leads with only one single inpatient stay (later even performed on an outpatient basis) to the definitive problem solution in adults and children [261], [262], [263], [264], [377], [379], [391], [392], [393], [394] and to less disease-related losses of earnings in comparison to ITE [264]. A disadvantage of TAC is the waiting time from the diagnosis to perform surgery (6–72 hours) [302], [376], [377], [382].
- ID: Paul from Aegina (625–690 A.C.), Guy de Chauliac (1362), and Chiari (1889) reported results after ID [356], [395]. Different trials showed that NA and ID are followed by a comparable outcome [304], [345]. In the study of Wolf et al., ID does not prove to be more successful [308] since it is associated with a higher recurrence rate within the first 2 months [308].
- NA: initially described by King (1961) [357], further trials confirmed reliable and almost pain-free therapy in adults, frequently performed on an outpatient basis. Rarely, a second NA becomes necessary [303], [305], [306], [307], ,[358] [375], [396]. In different studies, NA and ID proved to have a comparable outcome [304], [345]; in the study by Wolf et al. [308] ID was initially more successful than NA and in the study by Savolainen et al. [306] it was better in the following course.
- Recurrence rate: The recurrence rate was given with 0% to 23% in the literature analyzed by Herzon [261], [302], [303], [304], [305], [307], [308], [309], [310], [311], [313], [314], [350], [358], [378], [383], [386], [397]. An age <30 years [398], previous PTA [306], and a history of recurrent tonsillitis episodes [309] proved to be risk factors for the development of PTA recurrences, mostly within 2 to 12 months [306], [309]. Other reports could not identify a correlation between tonsillitis episodes in the patients’ history and PTA [310], [312], [313], [314].
- PTA after TE: 3 studies were cited [315], [322], [323].

In a separate part of his publication, Herzon dealt with the following questions:

1. What is the success rate of NA?
  In 10 studies with 496 patients, PTA was treated in 85% to 100% of all cases [303], [304], [305], [306], [307], [308], [345], [357], [363], [396].
2. What is the recurrence rate of PTA after NA/ID?
  19 trials with a total of 2,083 patients were encompassed, 272 of whom experienced recurrences (13%). The recurrence rates were significantly different (p<0.001) ranging between 0% to 22%. Interestingly, a recurrence rate of only 10% was calculated for studies from the USA vs. 15% for studies from outside the USA [261], [302], [303], [304], [305], [306], [307], [308], [309], [310], [311], [313], [314], [350], [358], [376], [378], [379], [383], [386], [397].
3. What is the rate of penicillin-resistant pathogens?
  Because of the publication date (1995) and the missing reference to the subject of this contribution, the issue will not be discussed here.
4. Are recurrent episodes of tonsillitis a risk factor for PTA?
  Herzon identified 14 trials revealing significant differences concerning the follow-up period and terminology (definition of tonsillitis) [302], [303], [304], [305], [306], [307], [308], [309], [310], [311], [312], [313], [315], [398]. Throat infections were reported for 11% to 56%. However, there were only 460 of 1,455 PTA patients (31.6%) with a history of tonsillitis prior to PTA. 63% of these 460 patients reported a history of 1 to 3 tonsillitis episodes.

**Conclusion drawn by the authors:** NA should be used as the initial surgical drainage procedure for all patients with a PTA other than those who have indications for abscess tonsillectomy. Patients should be treated in an outpatient setting, should receive penicillin if they are not allergic to it, and should receive adequate pain medication. The evidence does not suggest that there is any benefit in examining the abscess contents for microorganisms. Approximately 30% of patients with PTA can be expected to exhibit relative indications for a tonsillectomy.

##### 3.2.6.4 Table and conclusion on the indication of peritonsillar abscess

See Table 7 (Tab. 7).

**Conclusion:** TE is appropriate for the treatment of PTA.

#### 3.2.7 Tonsillitis

##### 3.2.7.1 Practice essentials of tonsillitis

This disease is characterized by the acute onset of sore throat, caused by a mostly viral inflammation of the palatal tonsils so that involvement of the adjacent pharyngeal mucosa cannot always be excluded [399]. In Middle Europe, almost only streptococcal angina and scarlet fever are clinically relevant bacterial inflammations. In the acute stage, antibiotic therapy rather than TE is indicated. Other typical bacterial pathogens of the head and neck region such as Haemophilus influenzae, Moraxella catharrhalis, Staphylococcus aureus, or anaerobes can be isolated to a high percentage from tonsillar specimens, but their pathophysiological relevance for tonsillitis is still unclear. Terms like “sore throat”, “chronic tonsillitis” and “recurrent acute tonsillitis” mixed lossely and poorly defined [400].

##### 3.2.7.2 Assessment by the Cochrane Collaboration [33]

The last update of this meta-analysis was published in 2014 [33]. Burton et al. had searched for RCTs to compare the efficacy of TE vs. antibiotic therapy of recurrent episode of tonsillitis. Primary endpoints were the efficacy of the intervention on the number and severity of sore throat episodes, days with sore throat, morbidity, and mortality. Secondary endpoints were the consumption of analgesics, antibiotics, absences from school or work as well as the quality of life. The search strategy, methods of the literature assessment, and requirements for formulating recommendations were clearly explained (*high quality*: further research is very unlikely to change our confidence in the estimate of effect; *moderate quality*: further research is likely to have an important impact on our confidence in the estimate of effect and may change the estimate; *low quality*: further research is very likely to have an important impact on our confidence in the estimate of effect and is likely to change the estimate; *very low quality*: we are very uncertain about the estimate). The authors included all randomized trials published until June 30, 2014. After applying several inclusion and exclusion criteria, 5 studies remained with a total of 987 children [401], [402], [403], [404] and 2 studies with a total of 156 adults [405], [406]. The meta-analysis revealed that the quality of the data was not sufficient to draw conclusions exceeding the first postoperative year in children and 6 postoperative months in adults. Furthermore, the meticulous calculations of the authors revealed a reduction of 0.6 sore throat episodes (95% CI: –1.0– –0.1) in the intervention group (3.0 vs. 3.6 sore throat episodes of non-operated children) in the first postoperative year. However, 1 of those 3 sore throat episodes was caused by TE. Distinguishing in children being “more severely affected” and “less severely affected”, the difference between the intervention and control group for more severely affected children was 1.1 vs. 1.2 sore throat episodes (95% CI: –0.6–0–0.4), which is not significant. In less severely affected children, the pain associated obligatorily with TE resulted in an inversion of the effect: 1.2 sore throat episodes compared to 0.4 episodes in the control group (95% CI: 0.7–0.9), resulting from pain associated with the intervention. Regarding the adult patients, the number of sore throat episodes was reduced within the first 6 postoperative months by 3.6 (95% CI: –7.9–0.7). The studies, however, were described as very heterogenic, the effect of the intervention reduced the number of sore throat episodes in one study by 1.5 (95% CI: –2.3– –0.7) [405] and in another one by 5.9 (95% CI: –7.8 to –0.7) [406]. Since the preoperative severity of the disease was not imprecisely described, a more specific evaluation as in children (see above) was impeded. Regarding the calculation of the days with sore throat, the children gained 5 days after TE (23 vs. 18 days). Omitting the low-quality study by Lock et al. [401], this gain is reduced to 4.3 days (95% CI: –8– –1.3 days). In the context of the adults, the data quality was qualified as low and very heterogenic. The study published by Alho et al. revealed a gain of 8.9 days by TE (95% CI: –14– –3.9 days) compared to 35.1 days in the study of Koskenkorva et al. (95% CI: –54– –16.2 days). Days with postoperative pain were not included, presumaybly 13±4 days [401] and 17±6 days [406]. For children, an analog calculation could be performed based on the studies of Paradise et al. Days with pain after TE amounted to 4.9 and 6.3 (0–21) [404]. Deaths did not occur in any of the studies, postoperative bleeding complications (all degrees of intensity) were observed in 2 to 6%. However, complication data were not collected by Lock et al. [401]. The data regarding consumption of antibiotics and analgesics were infrequently collected, thus impeding any conclusions. The absences from school could be reduced by 2.3 days (95% CI: –3.4– –1.2 days [403], [404]), however, the absences due to surgery were not included in this calculation. In the control group, the children were absent on average for 6 days. For adults, there was no significant difference regarding the absences from work that were only reduced by 3.3 days after TE (95% CI: –7.7–1–1.1 days) [406]. The impact of the different study designs on the results can be well seen based on the heterogeneity index I² that was given for some issues. For example it amounted to 64% for sore throat episodes after 1 year comparing 3 studies [402], [403], [404] and 57% when the trial of Paradise et al. from 1984 was included [403]. In the context of moderate to severe sore throat episodes, the heterogeneity index was 0% [402], [403], [404]. Regarding the sum of days with sore throat after one year, the I² value was 43% [401], [402], [403], [404]. For adults, an I² value of 85% was calculated for the absences after 6 months [405], [406].

**Conclusion drawn by the authors:** Adeno-/tonsillectomy leads to a reduction in the number of episodes of sore throat and days with sore throat in children in the first year after surgery compared to (initial) non-surgical treatment. Children who were more severely affected were more likely to benefit as they had a small reduction in moderate/severe sore throat episodes. The size of the effect is very modest, but there may be a benefit to knowing the precise timing of one episode of pain lasting several days – it occurs immediately after surgery as a direct consequence of the procedure. It is clear that some children get better without any surgery, and that whilst removing the tonsils will always prevent ‘tonsillitis’, the impact of the procedure on ‘sore throats’ due to pharyngitis is much less predictable.Insufficient information is available on the effectiveness of adeno-/tonsillectomy versus non-surgical treatment in adults to draw a firm conclusion.The impact of surgery, as demonstrated in the included studies, is modest. Many participants in the non-surgical group improve spontaneously (although some people randomised to this group do in fact undergo surgery). The potential ‘benefit’ of surgery must be weighed against the risks of the procedure as adeno-/tonsillectomy is associated with a small but significant degree of morbidity in the form of primary and secondary haemorrhage and, even with good analgesia, is particularly uncomfortable for adults.

##### 3.2.7.2.1 assessed in the review article of the Cochrane Collaboration: Paradise et al. [403]

In the randomized trial published by Paradise et al. in 1984 [403], 2,043 children aged between 3 and 15 years were included, but only 187 were eligible for evaluation (9.2%). 91 children were randomly assigned to groups either undergoing surgery or conservative treatment, for 96 children the decision was made by the parents. The condition of the pediatric patients was assessed in a standardized way: every 2 and 6 weeks by telephone calls and in cases of acute tonsillitis by clinical examination. An evaluation was performed after 2 and 3 years. 3 years after therapy, 34 of 95 (35.8%) of the surgery group and 13 of 92 (14.1%) of the conservative treatment group were lost to follow-up. Furthermore, 20 of 95 (21%) from the study group and 11 of 92 (12%) of the control group did not completely finish the evaluation year. In the randomized population, 4.62 infections occurred within 3 years after surgery (among those 0.51 considered as moderate or severe) in contrast to 7.95 infections after conservative therapy (among those 2.65 considered as moderate or severe). In summary, the authors could confirm a significant benefit of surgery only for the first two years despite strict indication and thus selection criteria and afterwards only a tendency was described.

**Conclusion drawn by the authors:** When children meet the stringent selection criteria, TE is beneficial in comparison to antibiotic treatment, at least for the first 2 years.

##### 3.2.7.2.2 assessed in the review article of the Cochrane Collaboration: Paradise et al. [404]

In 2002, Paradise et al. published a synchronous 2- and 3-arm trial including the data of 328 children, 3 to 15 years of age [404]. The 3-arm trial compared the outcome following ATE vs. TE and conservative therapy (58, 59, 60 children, respectively), while the 2-arm study compared the outcome after ATE vs. conservative therapy (73 and 78 children, respectively). The selection criteria for the number of episodes, severity of the disease, or medical documentation were lower than former study [403] but more stringent than in the Clinical Practice Guideline of the North American ENT Society at that time (at least 3 tonsillitis episodes per year despite conservative therapy). The episodes were subdivided in “counting” (sore throat plus oral temperature ≥38.3°C or size of cervical lymph nodes ≥2 cm or tonsillar exudation or positive GABHS proof), “intermediate” (sore throat plus oral temperature between 37.0°C and 38.3°C) as well as “minor” (exclusively sore throat). A “qualifying unit” was defined as 1 “counting” or 2 “intermediate” or 3 “minor” episodes. The authors classified the disease based on a 3 level system with regard to intensity of the pain, maximum body temperature, reduced general condition, red mucosa, and involvement of the cervical lymph nodes (score ≤2: “mild”; 3–5: “moderate”; ≥6: “severe”). Surgically treated children did not benefit significantly in comparison to conservatively treated children who, however, developed only 0.16 to 0.43 tonsillitis episodes per year on average. The postoperative pain persisted for an average of 6.3 days (0–21 days). The trial revealed that additional AT had no impact on the outcome. 

**Conclusion drawn by the authors:** The modest benefit conferred by TE or ATE in children moderately affected with recurrent throat infection seems not to justify the inherent risks, morbidity, and cost of the operations. Under ordinary circumstances, neither eligibility criteria such as those used for the present trials nor the criterion for surgery in current official guidelines are sufficiently stringent for use in clinical practice.

##### 3.2.7.2.3 assessed in the review article of the Cochrane Collaboration: van Staaij et al. [402]

Van Staaij et al. included 300 of 1,226 pediatric patients aged between 2 and 8 years in their randomized trial [402]. 151 children were assigned to the ATE group and 149 were assigned to the control group. The data of 155 children were evaluated, 133 of them underwent ATE within 6 weeks. The data were compared to those collected from 124 children with a wait-and-see policy. The treating physicians had to mention if recurrent tonsillitis episodes (≥3 per year), upper airway infections, or upper airway obstruction were the main indication for surgery. Children with severe infections were explicitly excluded. The parents were asked to write a diary and to measure the body temperature with automatic recording. The follow-up intervals were 3, 6, 12, 18 months after therapy onset; the examinations were carried out after an average of 22 months after study onset. In the ATE group 0.56 throat infections developed vs. 0.77 in the control group per person and year, regarding sore throat 2.25 vs. 2.85 episodes were calculated; 5.31 days of fever vs. 5.93 days in the control group were registered. 50 of 124 children from the control group underwent ATE after the end of the study based on the parents’ wish (40.3%). The authors concluded that ATE is effective only in cases of more than 3 tonsillitis episodes per year, independent from age, and that the effect is limited to 6 months in case of less frequent episodes. 

**Conclusion drawn by the authors:** ATE has no major clinical benefits over watchful waiting in children with mild symptoms of throat infections or adenotonsillar hypertrophy.

##### 3.2.7.2.4 assessed in the review article of the Cochrane Collaboration: Lock et al. [401]

This multicenter RCT with 268 children and a simultaneous cohort study with 461 children was published in 2010 [401]. The evaluation was performed for the age categories of 4–7 years, 8–11 years, and 12–15 years in both trials. TE was indicated for ≥4 episodes of tonsillitis per year within the preceding 2 years or ≥6 episodes within the last 12 months. After randomization, 131 were assigned to the surgical and 137 to the conservative group. TE or ATE was performed within 12 months after randomization. After 24 months, 11 children of the surgery group had not been operated (8.4%) but 36 children from the control group (26%). In the cohort study, 13 patients from the TE group were not operated and 9 patients of the control group had undergone a TE. The data collection was performed by means of booklets in 4-week intervals. Phone calls were made if the monthly feedback was not submitted, and the data were assessed by means of questionnaires 3, 12, and 24 months after inclusion in the study. Endpoints were the number of episodes of sore throat within the first 2 years, the number of medical consultations for these episodes, the number of symptom-free days, and the intensity of the pain with and without relation to surgery. Furthermore the costs and the quality of life (assessed by means of PedQL) were analyzed. The authors considered the conclusions highly limited because of the low response rate. Only 41.3% of the patients in the RCT and 28.5% of the patients of the cohort study documented adequately the monthly reports. In addition, a minority (approximately 50% in the RCT and 1/3 in the cohort study) filled out the questionnaire adequtely. In the RCT, the number of monthly episodes amounted to 0.64 and 0.33 in the control group and to 0.5 and 0.13 in the surgery group. The authors proved cost savings and clinically favorable outcome with improved quality of life by TE.

**Conclusion drawn by the authors:** Children and parents exhibited strong preferences for the surgical management of recurrent sore throats. The health of all children with recurrent sore throat improves over time, but trial participants randomised to surgical management tended to experience better outcomes than those randomised to medical management. The limitations of the study due to poor response at follow-up support the continuing careful use of 'watchful waiting' and medical management in both primary and secondary care in line with current clinical guidelines until clear-cut evidence of clinical effectiveness and cost-effectiveness is available..

##### 3.2.7.2.5 assessed in the review article of the Cochrane Collaboration: Koskenkorva et al. [406]

In this study [406], 46 of 86 adult patients with ≥3 episodes of pharyngitis during the preceding 12 months underwent TE within 8 and 23 days, while 40 patients were put on a waiting list. A precise differentiation between tonsillitis and pharyngitis was not made. The age of the patients was 27±11 years in the TE group and 26±8 years in the control group. An episode was defined as condition impairing the general well-being that resulted in a medical consultation and had resulted from the tonsils. Swab testing or culture for streptococci was not performed. The follow-up was limited to 6.2±.0.5 months after TE and 5.7±0.7 months after assignment to the control group. The patients were told to consult their physicians during the study phase in case of symptoms. In those cases, besides clinical examination, also a swab test was taken and CRP was determined, with CRP values repeated after 3 days. All data were recorded by the patients in a notebook including an evaluation of the quality of life by means of the Glasgow Benefit Inventory (GBI) 6 months after TE and a diary for mild/moderate/severe sore throat, cough, rhinitis, fever, and absences from school/work. Severe symptoms were those that required medical consultation, CRP values >40 mg/l, or pathologic findings of the swab test. The primary endpoint of the study was the reduction in the number of severe sore throat episodes. Secondary endpoints were the reduction of the number of mild/moderate sore throat episodes, the interval between the episodes, the average number of episodes, the absences, and the number of days with symptoms. In the TE group, the quality of life as well as complications was assessed. 2 patients of each group lost their notebooks, but they were symptom-free. 3 of 40 patients of the control group (8%) had to undergo TE because of severe tonsillitis after the waiting time. Regarding all endpoints of the study, the results after TE were significantly superior to those of the control group. Episodes of severe intensity were very rare in both groups (1 vs. 0). The number of episodes could be reduced by TE with (38%) and without (41%) medical consultation in comparison to the control group [406].

**Conclusion drawn by the authors:** Tonsillectomy resulted in fewer symptoms of pharyngitis, consequently decreasing the number of medical visits and days absent from school or work. 

##### 3.2.7.2.6 assessed in the review article of the Cochrane Collaboation: Alho et al. [405]

Alho et al. included 70 adults aged between 16 and 42 years in this study [405]. After computer-based randomization, 36 patients underwent TE within 13 days (8–21 days) and 34 patients were put on a waiting list for surgery after 3 to 6 months. An episode was defined as sore throat persisting without interruption for at least 2 days. For every patient at least once identification of streptococci by means of bacteriological culture or quick test was required. Patients were included who had at least 3 such episodes within 6 months or 4 within 12 months. 90 days after randomization, patients experienced less frequently sore throat episodes with and without positive testing for streptococci after TE compared to the control group (1 vs. 8 patients). Sore throat was frequently associated with fever and led significantly more often to medical consultation and absences from work. This is probably why 2 of 34 patients of the control group underwent anticipated TE (6%). The mean duration of the follow-up period was comparable between both groups: 170±12 vs. 164±63 days. One patient of the control group did not undergo surgery after the waiting time because the symptoms had disappeared. In total, the number of tonsillitis episodes per year could be reduced by 3.3 and the absences from work by 20 days after TE. Secondary bleeding was observed in two cases on the 9^th^ and 11^th^ postoperative day, respectively, but they did not require surgical treatment. In comparison to the control group, TE reduced the number of episodes with (30%) and without (25%) medical consultation.

**Conclusion drawn by the authors:** Adults with a history of documented recurrent episodes of streptococcal pharyngitis were less likely to have further streptococcal or other throat infections or days with throat pain if they had their tonsils removed.

##### 3.2.7.3 Assessment by other systematic reviews or meta-analyses

##### 3.2.7.3.1 assessed by Barraclough and Anari [407]

This research of the literature [407] was limted to the time between 1960 and July 2013 and younger patients (≤16 years). 8 trials focussed on the effectiveness of TE (endpoints: number/severity of episodes), 5 of them were published 1970 and earlier. Because of methodical reasons, they appear to be inappropriate [408], [409], [410], [411], [412]. The remainder were published by Paradise et al. [403], [404] and van Staaij et al. [402] and under certain circumstances they proved the superiority of surgical therapy in comparison to conservative therapy. The authors further identified studies that discussed the costs of the therapeutic modalities as endpoints [401], [413], [414]. Buskens et al. could not reveal a significant cost reduction for the surgical approach and even higher costs for children between 2 and 8 years of age with only mild symptoms [413]. This finding contrasts to reports of Lock et al. who also registered a better outcome in children between 4 and 15 years of age after TE [401], [414]. One of the trials cited by Barraclough and Anari was not randomized and controlled and quantified postoperative satisfaction of the parents and quality of life, psychological changes and impact on the body growth in operated children [415], [416], [417], [418], [419], [420], [421], [422], [423], [424], [425], [426], [427], [428], [429], [430]. Only 6/16 studies focussed on sore throat, 3 of them did not discuss the issue [419], [429], [431]. Successful outcome after TE was reported by Conlon et al. [424], Goldstein et al. [416], and Fujihara et al. [427], All parents of 80 children (2 to 14 years) were satisfied as documented in a questionnaire. Surgery had been indicated when more than 4 episodes had occurred within the last 2 years or more than 5 during the preceding year. Endpoints were the development of the symptom severity of throat inflammations, behaviour during sleep and in general [424]. Fujihara reported on the superiority of TE in 25 children (2 to 15 years of age) as well as 16 adults (endpoints: number of episodes with fever/medical consultations/absences from school or work one year before and after TE) and they complied to the US American guideline [432]. For children, the costs after TE were amortized after 1.6 years and for adults after 2.5 years [427]. By means of a validated questionnaire, Goldstein et al. assessed the course of 38 children (2 to 16 years of age) with ≥3 tonsillitis episodes or ≥3 antibiotic therapies, or ≥3 months of continuous sore throat in their history. Those specific symptoms as well as the quality of life were described as having clearly improved in this population [416]. Barraclough and Anari refer to a publication by Blakley and Magit [426] stating that surgical therapy reduced the number of pharyngitis episodes by 43%, however, the effect is more than moderate compared to conservative therapy.

**Conclusion drawn by the authors:** The disparity between parental satisfaction rates and published clinical efficacy can be explained by a lack of parent/child outcome measures specific to tonsillectomy for recurrent sore throats. A more parent/child-centered approach may establish what tonsillectomy could offer this group of children.

##### 3.2.7.3.2 assessed by Blakley and Magit [433]

For this meta-analysis [433], RCTs published until 2007 were included. Patients with sore throat (pharyngitis) either had undergone surgical or conservative therapy with documented sore throat episodes before and after therapy. The aforementioned studies of Paradise et al. (1984 and 2002), van Staaij et al. (2004), and Alho et al. (2007) were included for analysis [402], [403], [404], [405]. The outcome of the children in both studies by Paradise et al. was listed by years and the trial by van Staaij et al. was classified into “sore throat” and “throat infections”. Calculating the odds ratio, the 95% CI of all trials was nearly 1, apart from the study by Alho et al. which corresponds to a non-significant effect of the intervention. The evaluation of all studies resulted in an odds ratio of 0.569 (95% CI: 0.433–0.748; p=0.000) which means a reduction of the sore throat episodes of 43%. Focussing on the results after one year, there is an analog result, the odds ratio was 0.502 (95% CI: 0.323–0.782; p=0.002). An impact of additionally performed AT on the clinical results could not be verified. There was no difference in the outcome between adults and children. The monthly *number-needed-to-treat* was 11 (7–23) in the first year, i.e. 11 interventions had to be performed in order to avoid one sore throat episode.

**Conclusion drawn by the authors:** Tonsillectomy reduces the incidence of recurrent pharyngitis to a modest degree.

##### 3.2.7.3.3 later excluded assessments [434, 436, 437]

Georgalas et al. performed a systematic literature research in order to answer the question whether or not children and adults suffering from sore throat benefit from TE [434]. All articles published between 1966 and April 2014 were included (systematic review articles, RCTs, at least blinded with >20 patients of which >80% were followed for >6 months). The endpoints of the study were the number of episodes as well as absences from school or work. Additional endpoints of the research were the bleeding complications associated with the surgical technique and pain as well as the consumption of analgesics. The text is preceded by an (arbitrary) definition of severe throat infection: ≥5 tonsillitis episodes per year with symptoms persisting for ≥1 year leading to an impairment of the regular functionality [435]. No data were available regarding the natural course, the spontaneous healing observed in children was deduced from reports found in the literature [403], [404], [409], [410]. The reviewers stated to have identified 3 systematic review articles. However, only 2 were available for the present trial [436], [437]. One of them probably used wrong bibliographic data (similar to Alho et al.) [438], in addition, the used numbers were not transparent. The reviewers found another trial from 2010 [406] and thus evaluated 8 RCTs (further explanations not given, since studies with unclear definitions were included [408], [410], [438]). The remainder has been extensively discussed already [401], [405].

##### 3.2.7.4 Table and conclusion on the indication of tonsillitis

See Table 8 (Tab. 8).

**Conclusion:** TE is appropriate for treatment of recurrent acute tonsillitis episodes.

### 3.3 Guidelines

#### 3.3.1 France [439]

The guidelines commission dealt with different questions [439] (what are the indications of TE?; which preoperative diagnostics are required?; which surgical techniques can be applied?; which selection criteria can be used for outpatient surgery ; how to proceed with follow-up?, how should complications be treated?) and classified the recommendations with grades of the levels of evidence (low, moderate, high level of evidence). The results were not stratified by age and the relevant scientific literature was not cited. The indication for TE is predominantly justified by upper airway obstruction due to adeno-tonsillar hyperplasia (2/3) and only by one third by recurrent tonsillitis. The national indication in France seems to be: ≥3 tonsillitis episodes per year within the last 3 years or 5 tonsillitis episodes per year within the last 2 years. In contrast to Stuck et al. [400], the term of “chronic tonsillitis” is defined by local symptoms such as sore throat, halitosis, inflammation signs of the tonsils, and regional symptoms like cervical lymph node swellings for at least 3 months that do not respond to conservative therapy. The authors considered:

High level of evidence:

- Clinical examination and assessment of the tonsillar size, the cranio-facial proportions, and the upper airway morphology in cases of SBD
- In cases of upper airway obstruction, all other possibilities should be excluded.
- Suspected malignoma
- No indication for surgery: recurrent pharyngitis
- No indication for surgery: tonsils of different size without suspected malignoma

Moderate level of evidence:

- airway obstruction as indicated by symptoms occurring during nighttime (snoring, apnea, night sweat, enuresis, parasomnia, agitated sleep, abnormal sleeping position, head in hyperextension) or symptoms during daytime (difficulties to wake up, irritability when waking up, hyperactivity, attention and memory problems, asthenia when waking up, daytime sleepiness, headaches or nausea in the morning, lack of appetite in the morning, increased oral respiration, growth retardation)

Low level of evidence:

- TE without SBD with swallowing or voice disorders
- TE in cases or orofacial deformities with extensive tonsillar hyperplasia
- Chronic tonsillitis (as defined above)
- Recurrent PTA
- “other” infections such as PFAPA syndrome, post-streptococcal syndrome; infectious mononucleosis, drainage in cases of parapharyngeal abscess

#### 3.3.2 USA [432]

Recommendations of this guideline [432] are limited to patients aged between 1 to 18 years without relevant basic diseases (diabetes mellitus; genetic, cardiac, or neurological syndromes; sickle cell anemia; coagulopathies). The literature research was limited to the literature in Englsih language and included 2 guidelines of the last 10 years, 36 systematic review articles of the last 15 years, and 705 RCTs. The statements were given in a graduated way (strong recommendation; recommendation; option, no recommendation) depending on a benefit-risk analysis as well as the quality of evidence (A = good RCTs exist; B = RCTs with limitations or good observations studies exist; C = observations studies or case control studies exist; D = case reports or basic contributions; X = exceptional situations without possibility of carrying out studies). The authors discuss the indication as well as the planning, intraoperative (steroids) and postoperative measures (antibiotics, analgesics, bleeding complications, outcome assessment in cases of SBD). So the intraoperative application of dexamethasone is recommended, the necessity of perioperative antibiotic application is denied, and pain therapy training (also for parents) is recommended. An interesting recommendation is that surgeons should perform an annual analysis of the postoperative bleeding rate.

The authors emphasize the need of an adequate documentation of the symptoms, medical diagnosis/therapy as well as absences from school. The issue has been complained by Paradise et al. already in 1978 [440]. Simple measures have been suggested to improve the documentation system [441]. Die to the potential of spontaneous healing [402], [403], [404], [426], [433], [437], [442], [443], [444], [445], [446] a wait-and-see policy for 12 months is recommend prior to indicate TE. Patients with repeated inpatient treatment of tonsillitis, Lemierre’s syndrome, PTA, or a family history of rheumatic fever with cardiac or renal involvement are not subject of this recommendation.

The indication criteria for TE of Paradise et al. are re-confirmed to balance the risk-benefit ration n [403] (Table 9 (Tab. 9)).

The few RCTs [402], [403], [404] are criticized by the authors because of several weaknesses in the design. The meta-analysis by Burton et al. [447] of that time came to the conclusion that TE may result in a reduction of 1.4 episodes during the first postoperative year in comparison to the control group. However, TE itself is associated with painful swallowing. In cases of low to moderate pain intensity, the number of episodes in children who had undergone TE was only 0.2 lower compared to the control group. Blakley and Magit [433] calculated an odds ratio of 0.57, i.e. a reduction of sore throat episodes of 43% and a number-needed-to-treat of 11. According to van Staaij et al. [437] , TE reduced the number of sore throat episodes by factor 1.2, the number of absences from school by factor 2.8, and the number of airway infections by factor 0.5 – per year per patient. In summary, the benefit of TE is considered as very limited (“the guideline panel agreed there was not a clear preponderance of benefit over harm for TE, even for children meeting the strict criteria in the first study by Paradise et al.”) and the authors admonish to make very careful indications (shared decision making).

With regard to PFAPA syndrome, the evidence situation is characterized as rather poor with steroid application as effective alternative therapy and the possibility of spontaneous healing [234], [243], [244]. For PTA, TE as routine procedure is not recommended and the indication for abscesstonsillectomy is only seen for uncooperative patients, recurrent tonsillitis in the patients’ history, or PTA recurrences [301], [366], [448]. 

The indication in cases of pediatric autoimmune neuropsychiatric disorders associated with streptococcal infections (PANDAS) is refused because of at most hypothetic reflections [449].

A very individualized indication is finally suggested for a series of diseases that were not systematically investigated in studies (chronic tonsillitis, halitosis, muffled speech, tonsillar hyperplasia, malocclusion, tonsillar crypts, febrile convulsions, streptococci carriers).

With regard to SBD due to tonsillar hyperplasia, the authors assess TE to be advantageous. Interestingly, PSG is not required for TE in every pediatric patient but the assessment of disturbances of growth, enuresis, behavioral problems, and performance at school is recommended. The symptom of snoring only plays a minor role [92] as well as daytime sleepiness that is mostly observed in adults. Apparently, the total volume of adenoids and tonsils plays a major role compared to the tonsillar volume alone. Their size should be assessed according to the Brodsky index [450]. The effects of SBD on the quality of life, performance at school, body growth, bladder continence, and social behavior benefit from TE in ost cases [105], [156], [157], [161], [451], [452], [453], [454], [455], [456], [457], [458], but the severity of SBD does not correlate with the intensity of those symptoms which are not always present [453], [459]. Less than 10% of the children undergo preoperative PSG [454] and in only 60% to 70% of the cases, TE is successful according to PSG criteria [98]. In this context, the factor of obesity seems to be of high relevance that may reduce the success rate from 70% to 80% to 10% to 25% [97], [130]. Based on this guideline, tonsillar asymmetry is not considered as indication of TE, except for suspected malignoma [460], [461], [462].

The following recommendations with limitations were made:

Recommendation:

- Grade B: “watchful waiting” for recurrent throat infections
- Grade C: particularities such as multiple allergies / intolerance against antibiotics; PFAPA syndrome; recurrent PTA; halitosis; malocclusion; febrile convulsions; SBD

#### 3.3.3 Scotland [463]

The last update of the Scottish Intercollegiate Guidelines Network (SIGN) guideline entitled “Management of sore throat and indications for TE” was published in April 2010 [463]. A systematic literature review was performed including studies published between January 2000 and December 2008. The results of the RCT [401], [403], [404], [413] and a meta-analysis [447] could not determine any or only a limited effect of TE compared to conservative treatment. This statement contrasts to reports of a normalized body growth after TE [419], [464], [465]. For adults, a RCT [405] was confirmed the efficacy of TE, unfortunately limited by the follow- period of only 90 days. The rate of spontaneous healing in children and adults still remains unclear. The indication of TE is seen exclusively for recurrent acute tonsillitis and not for pharyngitis in general. Preoperatively, the patients, who already suffer from sore throat, have to be informed about the postoperative pain. In doubtful cases, a waiting period of approximately 6 months was recommended [463].

The guideline stated:

Recommendation grade A

- In children with only mild throat infections, watchful waiting is preferred to TE.
- In adults with severe throat infections, TE is indicated.

Recommendation grade D

- In cases of sore throat caused by acute tonsillitis with impairment of the regular functionality, TE is an option if the patients experienced a certain number of tonsillitis episodes during a certain time (7 during the last 12 months; 5 per year during the last 2 years; 3 per year during the last 3 years).

Good practice points

- In cases of doubt, a waiting-and-see policy of 6 months is recommended to assess the potential of spontaneous healing.

#### 3.3.4 Australasia [40]

For this guideline [40], only 30 literature references were included and the search strategy was not described. Recommendations were also classified from grade A to D, corresponding to the Australian requirements (NHMRC = National Health and Medical Research Council). The literature is cited in a rather short way and at best grade B is assigned. Also this guideline states that PSG is not a prerequisite to indicate TE in cases with SBD. Obviously, pneumococcal vaccination had no effect on the incidence of airway infections.

In summary, the guideline gives the following recommendations: 

Recommendation grade B

- TE in cases of moderate/severe upper airway obstruction due to adeno-tonsillar hyperplasia
- Snoring is no indication
- ≥7 tonsillitis episodes during the last year; ≥5 tonsillitis episodes per year during the last 2 years; ≥3 tonsillitis episodes per year during the last 3 years
- Suspected malignoma, especially malignant lymphoma [466]

Recommendation grade C

- TE for PTA with a history of recurrent tonsillitis episodes and comorbidities

Recommendation grade D

- Tonsilloliths, tonsillar cysts, spontaneous tonsillar bleeding, diphtheria carriers

#### 3.3.5 Italy [467]

This guideline updated a previous version in 2008 with comments on indications, surgical techniques, intraoperative and postoperative management [467]. The literature review was limited to publications from 1990 to October 2007. The data quality of the papers was classified into 6 levels (I: evidence from RCTa and/or systematic reviews of RCTs trials; II: evidence from one single adequately designed RCT; III: evidence from non-randomized cohort studies with concurrent or historical control or their meta-analysis; IV: evidence from non-controlled retrospective case-control studies; V: evidence from non-controlled case series; VI: evidence from experts’ opinions or opinions from panels as indicated in guidelines or consensus conferences, or based on opinions from members of the work group responsible for this guideline). Grades of recommendations were classified from A to E (A: carrying out the specified procedure or diagnostic test is strongly recommended. The recommendation is supported by good-quality evidence, even if not necessarily type I or II; B: it would be inappropriate to always recommend the specified procedure or intervention, considered the still existing doubts, but it should anyway be carefully considered; C: significant uncertainties against recommending to carry out the specified procedure or intervention; D: the specified procedure is not recommended; E: the specified procedure is strongly not recommended). This procedure corresponds to the national recommendations of the *Programma nazionale delle linee guida (PNLG)*. A watchful waiting policy is recommended for 6 months before indicating TE. The design of the guideline is unusual and the following classification limited to indications: 

I/A

- PTA in children and adults should be treated with antibiotics and ID.

II/A

- TE for recurrent episodes of acute tonsillitis in adults and children is indicated for: ≥5 tonsillitis episodes per year for at least one year with impairment of the normal functionality at each time. Patientes with less severe episodes responding to antibiotics are not candidates for TE

II/V/D

- PFAPA syndrome as indication is not sufficiently supported by the literature, and there is a strong a tendency for spontaneous remission.

III/A

- Pediatric SBD due to upper airway obstruction with adeno-tonsillar hyperplasia respond well to ATE/TE
- AT alone is not sufficient.

VI/A

- Severe cases of SBD due to upper airway obstruction with adeno-tonsillar hyperplasia should undergo surgery as soon as possible.
- In cases of complications related to PTA, inpatient observation is recommended.

VI/B

- In children with SBD, comorbidities should be treated such as obesity, recurrent upper airway infections, nasal obstruction, cranio-facial anomalies, macroglossia, or neuromuscular/orthodontic diseases prior to ATE/TE.
- The indication of recurrent acute tonsillitis can be made less strictly if a clear cervical lymph node swelling (>2 cm) persists after antibiotic therapy or ≥1 PTA was observed in the patient’s history or febrile convulsions, deformities of the airways, cardiovascular or other severe pathologic conditions are diagnosed.
- AT should be performed at the same time if indicated by the findings or symptoms.
- TE is an option for recurrent PTA
- TE should be considered for PFAPA syndrome with rapidly recurring fever and unfavorable course

### 3.4 Evidence-based indications – previous publications

Beside the single diseases and guidelines in the context of indication of TE, finally another group of articles could be identified that discussed evidence-based indication criteria of this surgical intervention. According to the above-mentioned search strategy, 4 articles published between 2004 and 2014 matched our search criteria [37], [468], [469], [470]. The publications summarized the status of knowledge at the time of publication, however, they are written in very different ways. The most recent papers are reduced to the indications in children [468], [469], a very short article dealt with the practice in England [470]. One paper [37] does not reflect the exhaustive literature review of the co-author Mund, obtainable from his doctoral thesis [38].

#### 3.4.1 Isaacson [468]

18 pages describe the development, histological figures, and the immune function of the tonsils as well as information on wound healing, indications, surgical procedures, postoperative complications/morbidities and their management [468]. In the summary, Isaacson classified several aspects into 3 categories of knowledge:

1. Things we know (or we are rather sure of to know)
  TE decreases the frequency of severe recurrent sore throats in children who meet the “Paradise criteria”.
  ATE improves symptoms of SBD in children with adeno-tonsillar hypertrophy and thus improves their quality of life.
  Obese children with SBD do not benefit from TE.
2. Things we know (that are possibly true)
  Children with mild courses of tonsillitis episodes, multiple antibiotic allergies, PFAPA syndrome, or PTA in their history benefit from TE.
3. Things we do not know (but should know)
  Which children suffering from recurrent sore throat episodes benefit from TE?
  Validity of rare indications (PFAPA syndrome, febrile convulsions, halitosis, malocclusion, pediatric neurological deficits due to streptococcal infection-associated autoimmune reaction (PANDAS).
  Value of TE in comparison to TOTO in the long-term course of upper airway obstruction and recurrent acute tonsillitis.

SBD, as a result of adenotonsillar hypertrophy was acknowledged as a valid indication for TE [471], [472]. Snoring was accepted as indication for TE only, if accompanied by growth retardation, enuresis, bad school performance, or behavioral problems. In difficult cases, PSG is recommended. The second major group of indications was related to tonsillitis [401], [402]. However, a subgroup of children without spontaneous healing could not be identified by the results of the aforementioned studies. Other indications were called “relative” and were only listed in a table (PTA, peritonsillitis, PANDAS, “chronic” tonsillitis, febrile convulsions, halitosis, malocclusion, tonsillar crypts, hemorrhagic tonsillitis, rheumatic fever, permanent carriers of ß-hemolytic streptococci of group A).

#### 3.4.2 Oomen [469]

On 10 pages, the authors dealt with different aspects of the intervention such as frequency in the USA, indications, diagnostics, PSG, surgical procedures, postoperative morbidity, and postoperative follow-up concepts [469]. The authors cited the well-known trials of Paradise et al. (level 1b) and iterated the known contents: indications for surgery only for severe courses of recurrent acute tonsillitis [404] which was also recommended in the guideline published by Baugh et al. [432]. With reference to a publication of Friedmann et al. (level 2a) [98] and the contemporary guideline of the USA [432], TE is recommended for SBD caused by adeno-tonsillar hyperplasia with grade of recommendation B-C. Detailed information concerning halitosis, malocclusion, or tonsillitis were not provided [447], [473], [474]. 

#### 3.4.3 Munir and Clarke [470]

This article [470] consists of 3 pages and cited the Scottish guideline [437] suggesting the following indication criteria: origin of sore throat episodes for ≥1 year, impaired daily life and ≥5 episodes of sore throat per year. The cited US American guideline stated for children: ≥3 tonsillitis episodes per year [475]. In relation to SBD, only Smith and Pereira [476] were cited. Based on the American guideline, children with SBD are good candidates for TE or TOTO [475]. Concerning recurrent tonsillitis episodes, the authors cited the most significant papers [434], [436], [447], [477] and stressed the low data quality and positive reports concerning an improved quality of life and high postoperative patient satisfaction after TE [416], [426], [429], [478], [479]. The contents of the RCTs of Paradise et al. [403], [404] and van Staaij et al. [402] were briefly described. Supported by Khayr and Taepke [480] TE is indicated for PTA in patients with a history of recurrent tonsillitis. Further publications were not mentioned. SBD as indication for TE with potential benefit in the long-term was shortly described with only few studies cited by the authors [104], [105], [477], [481], [482]. Hemorrhagic tonsillitis as indication for TE was suggested but not supported by citations. Tonsillar asymmetry without an additional sign of a malign disease is not considered as valid indication [483] and in children it is considered as being probably due to the surrounding muscles. The authors concluded, that recurrent acute tonsillitis and SBD were the most frequent indications for surgery and surgery should be indicated after individual patient selction.

#### 3.4.4.1 Wolfensberger and Mund [37]

Wolfensberger and Mund [37] reviewed and classified the literature of the past 25 years according to the levels of evidence. Articles on tonsillogenic focus diseases were not cited explicitly in this article. Primary diseases of the tonsils in children (≤12 years) and adults (>12 years) and numerous attributable diseases were discussed in brief (for details: 3.4.4.2). Recurrent acute tonsillitis prevailed as indication for TE, only 25% were associated with SBD. Enuresis, halitosis, simple snoring only played a role as co-factors, but they were no independent factor. A historical overview included the studies of Kaiser, Roydhouse, and Mawson [408], [412], [484] and the aforementioned RCTs of Paradise et al. as well as the review of Marshall [436] and Burton et al. [485]. In relation to SBD, ATE was associated with a success rate of 66% to 90% and improvement to some extent was registered for almost all patients (data not proven by RCT). Infectious diseases of the tonsils prevailed as indication for TE in adults, including PTA. In contrast to children, spontaneous remission of recurrent acute tonsillitis does not occur in adults. Only one single RCT [486] was identified that could not confirm a benefit of TE in comparison to conservative therapy. This was in contradiction to an own study of the authors, a trial published by Laing and McKerrow [487] and a study of Mui et al. [488]. In cases of PTA, needle aspiration and incisional drainage were recommended as primary measure, with a risk of recurrence of about 10%. Abscesstonsillectomy could only be recommended for recurrent PTA and is not adequate to prevent recurrences. Regarding a history of recurrent acute tonsillitis, the indication for surgery could be made more generously. TE in 23 Patients with a proven infectious mononucleosis resulted in a significant shortening of the reconvalescence. But also an increased susceptibility for a recurrence [489]. Tonsillar asymmetry was acknowledged as proper indication for TE, potentially as bilateral procedure. Other indications like snoring or halitosis were poorly defined and TE not recommended for snoring. In contrast to children, TE without adjuvant therapy is not an option for adults with SBD. 

#### 3.4.4.2 Mund [38]

Complementary details to 3.4.4 are obtainable from this doctoral thesis [38]. The author had performed a Medline review. 428 of initially 3,453 screened articles were eligible for analysis. Filters included publication date (1967 to the beginning of 1998) and language (German, English, French) and the literature classified according the levels of evidence-based medicine:

- Type A studies: highest level of evidence
  Meta-analyses, RCTs, systematic and exhaustive reviews, prospective-controlled but not randomized studies (the study conditions were actively changed by the investigators, which is important when RCT is not possible due to technical or ethical reasons).

- Type B studies: middle level of evidence
  Review articles with several non-experimental studies (including high-quality descriptive studies), non-experimental studies, observational studies (without active changes of the study conditions, generally retrospective, non-randomized trials), high-quality descriptive studies, reports on follow-up of an investigated group of ≥50 participants (in general, assessment of patient files, long-term trials with follow-up over at least 5 years, non-controlled, non-randomized).

- Type C studies: lowest level of evidence
  Descriptive studies, follow-up reports of investigation groups with <50 participants (in general, assessment of patient files, reduced follow-up), case reports, expert opinions.

The literature was classified according to the following 6 groups of indication. The very heterogenic quality of the studies was criticized by Burton et al. already who therefore excluded several trials [33].

1. Infectious diseases

- Recurrent acute tonsillitis
  The efficacy of TE in children with recurrent tonsillitis is proven by several studies. Regarding adults, only one low-quality study was performed that could not confirm a benefit after TE [486]. The number of “necessary” tonsillitis episodes for indication of surgery could not be clearly determined by the literature. It is expected to be more than 3 per year. It is noteworthy to repeat that the number of medically confirmed tonsillitis episodes is certainly lower than the one reported by the parents. “Chronic tonsillitis” is not a scientifically defined term. Since the disease itself is unclear, a recommendation regarding indication for TE could not be given. In contrast to adults, children experience a decreasing number of tonsillitis episodes with time [13], [180], [403], [409], [410], [436], [438], [447], [484], [486], [487], [490] (grade A), [478], [488], [491] (grade B).

- PTA
  RCT were not found the the review. In compliant patients, NA seems to be adequate. If anesthesia is necessary for drainage, abscesstonsillectomy is an option. Abscesstonsillectomy in children is clearly indicated if the patient has a history of recurrent tonsillitis (about 20–30% of the children) or if NA had failed. The hemorrhage risk after abscesstonsillectomy is comparable to elective TE. ITE after successful NA is not required. With regard to adults, several scientifically high-quality studies including a systematic literature research exist. NA is suggested as first-line therapy for PTA, alternatively ID. Abscesstonsillectomy is indicated in patients with a history of recurrent tonsillitis or PTA [13], [263], [301], [349], [364], [398], [492], [493], [494] (grade A), [307], [386], [495] (grade B).

- Infectious mononucleosis (IM)
  In childhood this infection is clinically not relevant, trials with children were not found. Common cases of IM are not an indication for TE. If complicated by airway obstruction or tonsillar hemorrhage TE is an option to alleviate the symptoms. Although TE can reduce the duration of the disease but may also compromise the immune system [489] (grade A), [496] (grade C).

- Plaut-Vincent angina
  Studies involving adults or children were not found.

- Sinusitis
  No scientific data suggest TE for (rhino)sinusitis sinusitis in children or adults [497], [498], [499] (grade B).

- Bronchitis/cough
  TE is not an option to treat bronchitis/cough, trials do not exist [409], [410] (grade A).

- Chronic otitis media with effusion
  Several RCT could show that TE is not appropriate to treat otitis media with effusion [13], [54], [410], [411], [500], [501], [502], [503] (grade A).

2. Upper airway obstruction

- Tonsillar hyperplasia without symptoms
  The literature does not justify TE for this pediatric subgroup [13] (grade A), [192], [504] (grade B).

- Snoring
  In children, snoring may be alleviated by TE, but the risk-benefit ratio is not in favor for the intervention without additional symptoms. Daytime sleepiness, impaired vigilance, frequent headaches may indicate an OSAS. Snoring in adults itself is no indication for TE. The intervention has a little prophylactic value in children [13], [180], [409], [505], [506], [507] (grade A), [508], [509] (grade B).

- OSAS
  The benefit of ATE in otherwise healthy children with OSAS is clearly documented with several RCTs. TE apparently plays a key role. In contrast to children, TE is efficient only as part of the Uvuluo-Velo-Palatoplasty in adults with OSAS [13], [505], [507] (grade A), [99], [164], [166], [510], [511], [512], [513], [514], [515], [516], [517] (grade B).

- Rhinolalia
  Larger control studies with children do not exist, open nasality is rather deteriorated by TE than improved. Trials with adults were not found [518], [519] (grade C).

3. Focal and systemic diseases

- IGAN
  TE seems to have at least some value to be determined for children with glomerulonephritis and IGAN. In adults, several studies revealed a positive effect of TE on IGAN. [62], [84], [88] (grade A), [83], [87], [520], [521], [522], [523] (grade B).

- Arthropathy
  In children, TE seemed to avoid arthritis occurring during rheumatic fever. Patients with recurrent rheumatic fever may benefit from TE. Scientific data do not suggest TE for reactive and focal arthritis in adults [524] (grade A), [522], [525], [526], [527] (grade B).

- Dermatoses
  Prospective studies with children were not found. Several retrospective investigations, revealed a benefit from TE to some extent in patients with psoriasis vulgaris as well as palmoplantar pustulosis. Adults suffering from psoriasis vulgaris, however, seem to benefit less from surgery [208], [520], [523], [525], [528], [529], [530] (grade B). Annotation: the above-mentioned psoriasis guttata is not explicitly mentioned.

- Intestinal diseases
  TE does not seem to have a protective effect on colitis ulcerosa or celiac disease. TE is under discussion to be a risk factor for chronic inflammatory bowel diseases but rials in adults or children are missing [531] (grade A), [532] (grade B).

- Cardiac diseases
  There were no relevant trials on this topic, one single study with adults did not seem to be appropriate for sound conclusions [527] (grade B).

- Unclear fever
  There were not studies involving children that would justify this indication [533] (grade B), [240], [534] (grade C).

4. Atopies

- Bronchial asthma
  In children, TE had no impact on bronchial asthma or allergic rhinitis. Studies with adults were not performed [535] (grade A), [527], [536], [537], [538] (grade B), [539] (grade C).

- Atopies
  Atopic diseases were neither an indication nor a contraindication of TE. Studies with adults were not found [535] (grade A), [536], [540] (grade B).

5. Non-classifiable indications

- Malocclusion, palatal cleft
  There seemed to be no correlation between tonsillar hyperplasia, oral breathing, and malocclusion. An interdisciplinary approach was suggested to justify TE for this group of indication [541], [542] (grade A), [543], [544], [545] (grade B).

- Halitosis
  In rare cases, the palatal tonsils may be an origin of halitosis and justify TE. Trial were not found [546] (grade B).

- Hemorrhagic tonsillitis
  Spontaneous tonsillar bleedings are very rare and usually affect adults, the indication for TE has to be individualized. In cases of suspected malignoma, the indication is not under discussion and clear [547] (grade B), [548], [549] (grade C).

- Growth retardation
  In the context of upper airway obstruction, children benefit from TE, the indication depends on the extent of the infections or the airway obstruction but not only the growth retardation itself [419], [550], [551] (grade B), [465], [552], [553] (grade C).

- Nocturnal enuresis
  Children with simultaneous symptoms of upper airway obstruction benefit from TE [151], [554] (grade B), [555] (grade C).

6. Special diseases

- Down syndrome
  Due to the common finding of a macroglossia, children with Down syndrome benefit from TE to alleviate the upper airway obstruction TE. Trials with adults do not exist [166], [556], [557] (grade B).

- Sickle cell anemia
  Recurrent episodes of tonsillitis may be followed by hemolytic fever or even aplastic crises. TE is therefore indicated [558] (grade A).

- Cerebral paresis
  In contrast to children with SBD without comorbidities, the possible benefit for children with cerebral paresis seems to be extremely limited [559], [560], [561], [562] (grade C).

## 4 Discussion

In the handbook of Denker and Kahler (1928), Zarniko published a chapter describing the intervention as “industrially exploited mania of surgery” (author’s translation) [563]. In 1969, Bolande classified TE as “ritual surgery” [564]. In “Tonsillectomy – Trials and Tribulations” from Terrence S. Carden (1978) stated that TE has lost none of its actuality topicality. One group accepted malign diseases, recurrences after PTA, and symptomatic airway and/or digestive pathway obstructions as indication for TE. A second group of physicians denied any value of th intervention [565]. In the 1970s, Jack L. Paradise and Charles D. Bluestone headed a committee that prepared a trial to demonstrate the efficacy of AT, TE, and ATE [413]. It should be noted that even today, the resulting trials are still cited in the contemporary scientific literature in national guidelines and publications [403]. Despite the extraordinary high frequency of the interventions [566], [567] plus the increasingly performed TOTO [107], [568] only few trials analyzed the clinical value of the interventions. This deserves a comment:

1. The application of filters in literature reviews is commonly associated with a reduction to a surprisingly low number of matching hits which is confirmed by the results of the present study, emphasized by the meta-analyses of the Cochrane Collaboration. The readers should keep in mind the risk of overlooking important trials, an aspect that will be discussed later.
2. The assessment of the literature discussed in this article according to the criteria of evidence-based medicine does not automatically mean a classification of the surgical indications into “correct” and “wrong”. According to Perleth and Raspe, evidence-based medicine just assesses the internal validity, i.e. the proximity of the observed to the real effect. Ideally, this is performed by systemativ reviews a meta-analysis. We therefore limited our literature review to this kind of scientific database to filter the best possible evidence from the large quantity of publications.
3. The evidence situation (meta-analyses, case control studies, cohort studies, case series, case reports, expert opinions) directly determines the grade of recommendation as for example in guidelines (see Table 10 (Tab. 10), Table 11 (Tab. 11), Table 12 (Tab. 12)). More recent approaches on grades of recommendation consider parameters such as the number-needed-to-treat or the CI to improve assessment of the data quality. We therefore explicitly searched for the CIs and p-values.
4. Serous otitis media is the only disease with a clear recommendation: TE is not indicated.
5. All other indications for TE still mandate further research. For instance, a most recent meta-analysis [57] revealed a positive impact of TE on IGAN, which was denied 4 years earlier [36].

The positive impact of ATE on children with SBD is only limited to an unclear extent in case of comorbidities. However, the indication is increasingly under discussion due to the rising acceptance of TOTO. The main advantage of TOTO is a significantly lower postoperative morbidity. However, the individually unpredictable risk of tonsillar regrowth has to be included in the informed consent prior to surgery. It should be emphasized, that TOTO rather than ATE is predominantly performed in Sweden since many years [16], [568], [569].

Psoriasis is a good example for the susceptibility for bias of literature reviews. According to our primary results, the obtainable data from the retrieved articles do not suggest TE for psoriasis. Unfortunately, the review of Sigurdardottir et al. was not identified with our search criteria which was based on an analysis of case series and case reports from the time between 1964 and 2012 including 659 patients (4 to 54 years of age) [197]. The authors concluded from the data that TE should be suggested in children and adolescents within the first year the first symptoms of psoriasis guttata. TE for adults was recommended only under certain circumstances such as a history of recurrent tonsillitis associated with severe psoriasis (>10% of affected body surface) with poor response to dermatologic therapies or immediate recurrence after stopping dermatologic therapy [205].

Garavello et al. concluded for PFAPA syndrome, that TE is not an option for therapy due inconclusive data and very heterogenic data quality. In the light of a potential spontaneous remission and the benefit from steroid therapy the value of the intervention appears at least questionable.

A differentiated consideration is also appropriate for PTA because less invasive methods such as ID or NA have turned out to be very effective [267]. Before indicating abscesstonsillectomy, several issues should be considered, such as the patient’s history and age, compliance, and potential complications. It is noteworthy to repeat the little prophylactic values ot TE on PTA recurrence [315], [322], [323]. Even after previous TE patients may experience an abscess formation. Moreover, a considerable number of patients deny a history of recurrent tonsillitis or PTA [295]. It appears, as if PTA is much more a different entity than a complication of tonsillitis. Probably the abscess-forming inflammation develops from remnants of the second pharyngeal pouch, or from Weber’s glands, or from the teeth [295], [296], [297], [298], [299]. ITE is generally not recommended or only, if an adequately skilled staff or instruments is not available [570], [571]. Elective ITE as a standard is not justified by the scientific data [301].

Recurrent acute tonsillitis is repeatedly mentioned as the most common indication for TE. According to the meta-analysis of Burton et al. this indication is supported by a low to moderate data quality. Surprisingly enough, only 2 relevant studies with adults and apart from the 2 trials from Paradise et al. only 2 others with children matched the search criteria of the Cochrane Collaboration. Therefore, high-quality RCTs are required to overcome this lack of scientific information. Interestingly, an increasing number of contemporary reports suggests rather TOTO than TE for tonsillitis, at least for some patients. It should be emphasized, that a history of tonsillitis apparently does not contraindicate a TOTO [16].

Diseases such as infectious mononucleosis, sinusitis, bronchitis, cough, arthritis, bowel diseases, cardiac diseases, bronchial asthma, or atopies were not mentioned in any of the systemativ reviews or guidelines. Moreover, blood values such as the anti-streptolysin (ASL) titer, were also not recommended in any of the retrieved articles. It is worth to repeat that the ASL titer is totally irrelevant for the indication of TE [572]. The prevalence of asymptomatic GABHS carriers varies significantly in the literature between 6% to 40% [573]. A study of 87 healthy persons did not reveal a significant correlation between the blood values and high-sensitive C-reactive protein. A correlation between the ASL-titer and clinical state was denied [574].

The available national guidelines were addressed for children [439], children and adolescents [432], or children and adults [40], [41], [467]. Interestingly, the indication criteria of Paradise et al. [403] were more or less included in all guidelines. Obviously, no further significant trails are obtainable from the literature databases. However, it should be emphasized, that both trials were designed for children and adolesents (3 to 15 years of age). In Italy, another criterion was applied, i.e. the assessment of the previous 12 months with at least 5 clinically relevant sore throat episodes [467]. Furthermore, a wait-and-see policy was recommended to better estimate the potential of a spontaneous remission. A comparable recommendation was suggested in the Scottish guideline [41], and in the USA even a 12-month watchful waiting period is recommended for the same reason [432].

## Summary

Sytematic reviews and meta-analysis of the last 30 years dealing with TE covered only 7 main diseases. 1) In cases of otitis media with effusion TE is not justified and further research is not indicated 2) For patients with PFAPA syndrome, benefit-risk ratio appears imbalanced due to the alternative steroid therapy and potential spontaneous remission in a considerable number of patients. 3) TE as first-line for PTA is not indicated and limited to patients with PTA recurrences, complications, a history of recurrent tonsillitis. ITE is not suggested. 4) Tonsillectomy in terms of ATE for pediatric OSAS patients resulting from adeno-tonsillar hyperplasia is substantially supported by scientific data. Comorbidities have a tremendous impact on the success rate, predominantly obesity. Further research is needed to clarify, whether or not the less-invasive TOTO is capable to replace ATE. 5) There is a growing tendency to suggest TE for patients with psoriasis guttata in children as well as therapy refractory psoriasis vulgaris in adults, but further large-scale studies are needed. 6) Further studies will have to clarify the values of TE for patients with IGAN. 7) If TE aims at healing streptococci-associated tonsillitis, the intervention competes with antibiotic therapy and an unpredictable rate of spontaneous remssion. In the light of the considerable postoperative morbidity it is imperative to evaluate a clear cut-off value for the number of episodes required to suggest TE in children, adolescents and adults rather than any other treatment. 

## Notes

###  Competing interests

The author declares that he has no competing interests.

## Figures and Tables

**Table 1:**
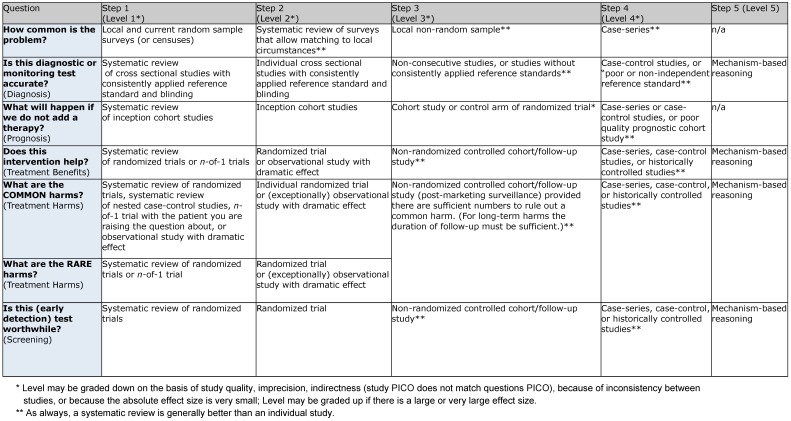
The Oxford 2011 Levels of Evidence [31] (Licensed under a Creative Commons Attribution 4.0 International License https://creativecommons.org/licenses/by/4.0/)

**Table 2:**
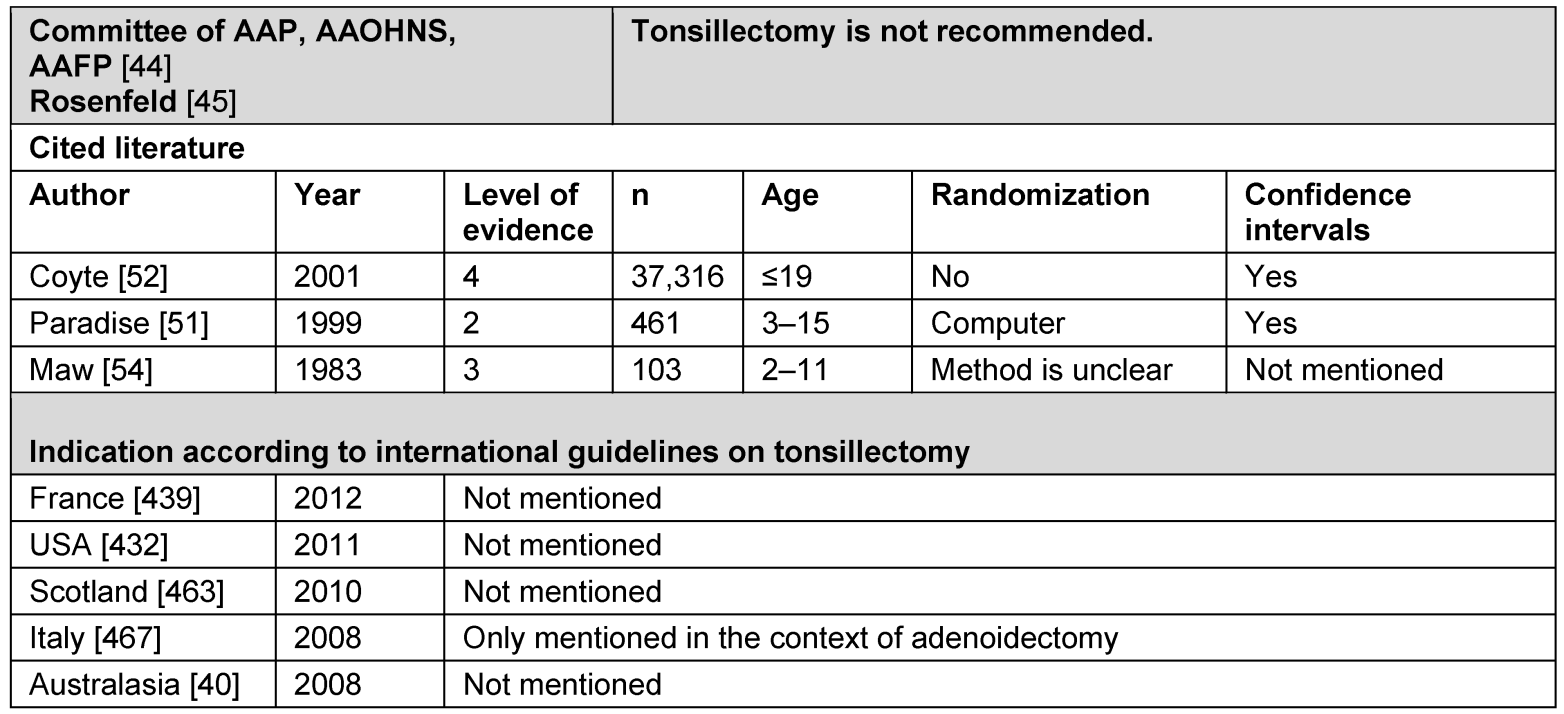
Trials and guidelines on the indication of otitis media with effusion

**Table 3:**
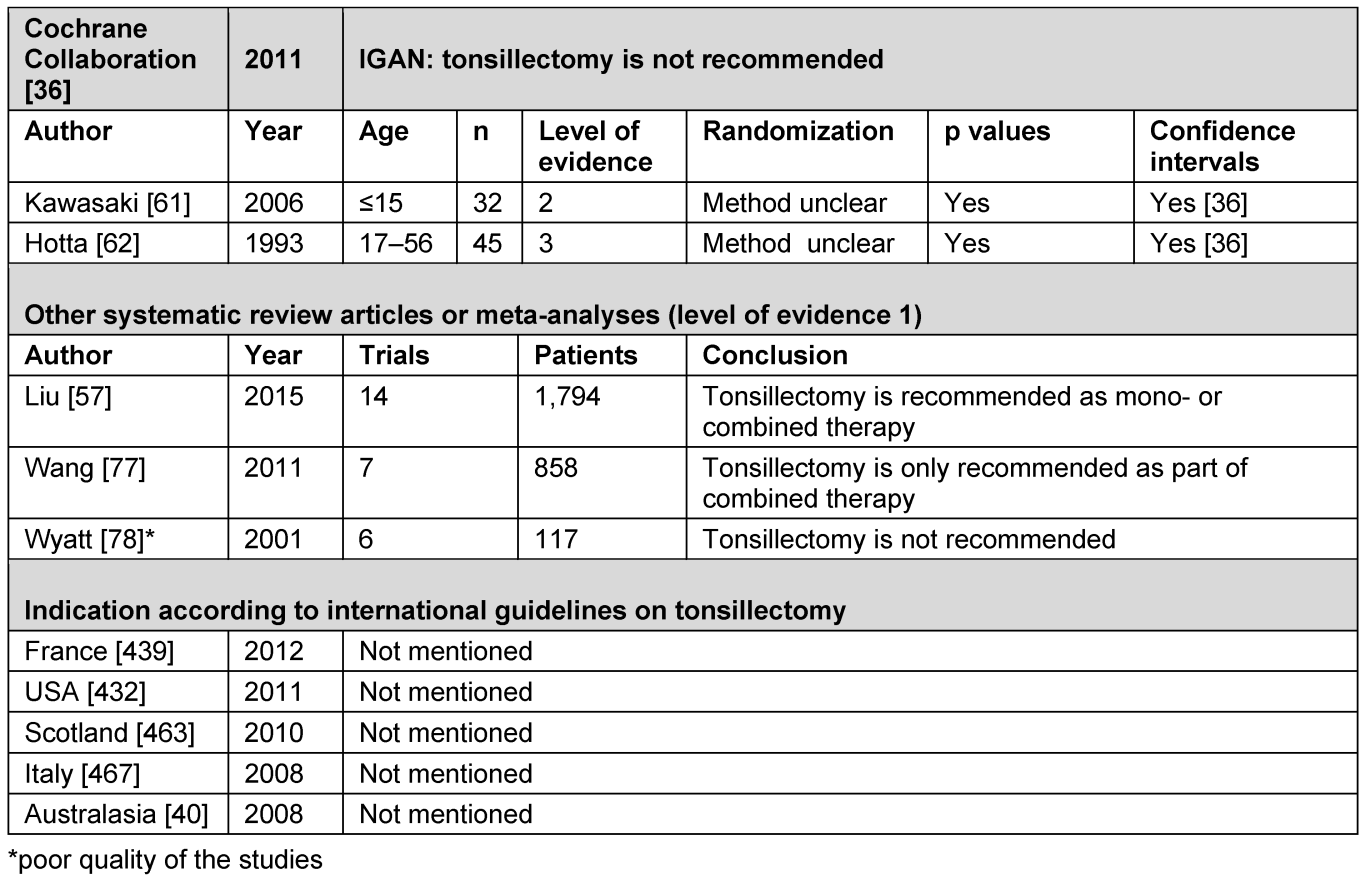
Trials and guidelines for the indication of IGAN

**Table 4:**
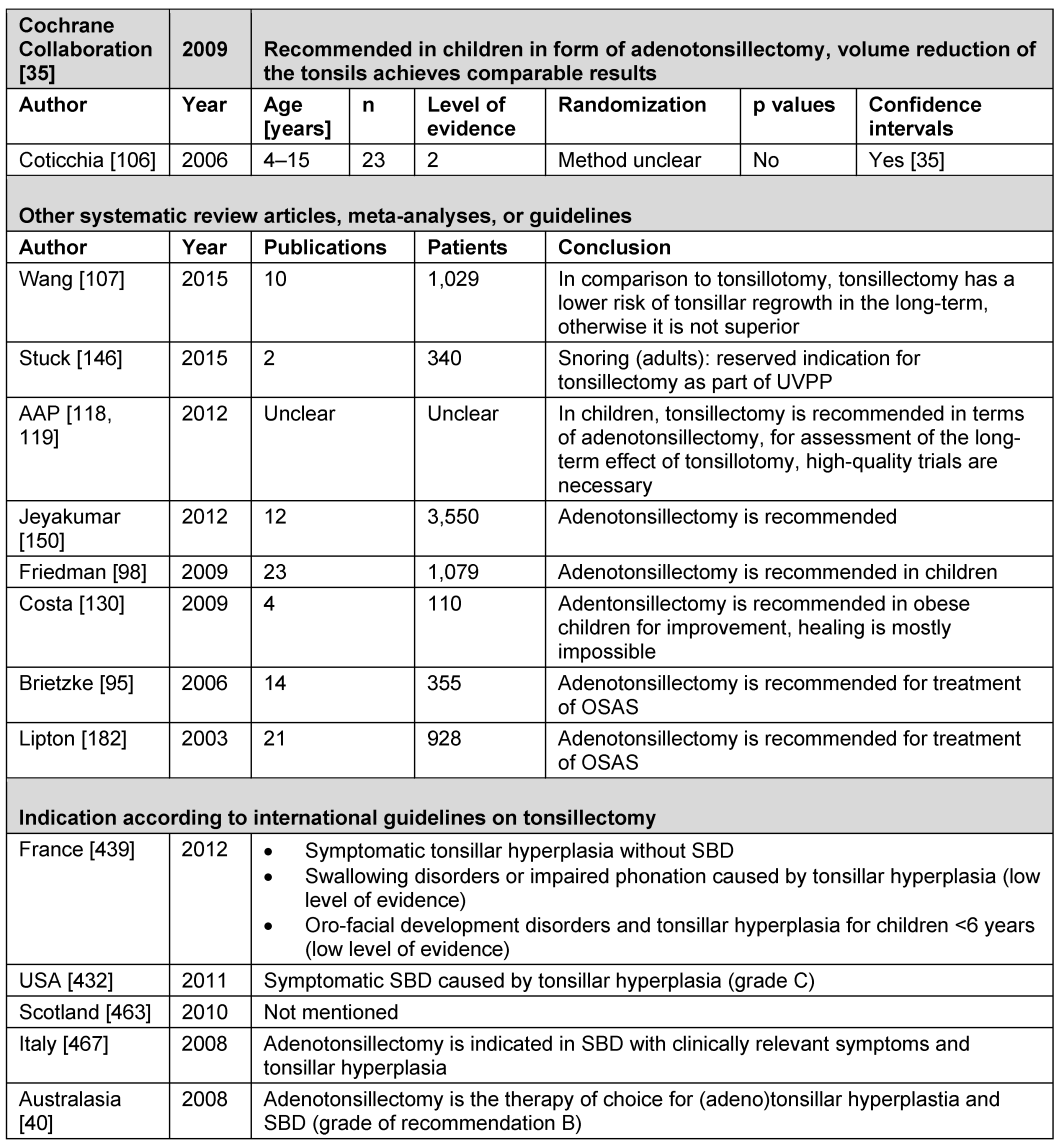
Trials and guidelines on the indication of sleep-related breathing disorders

**Table 5:**
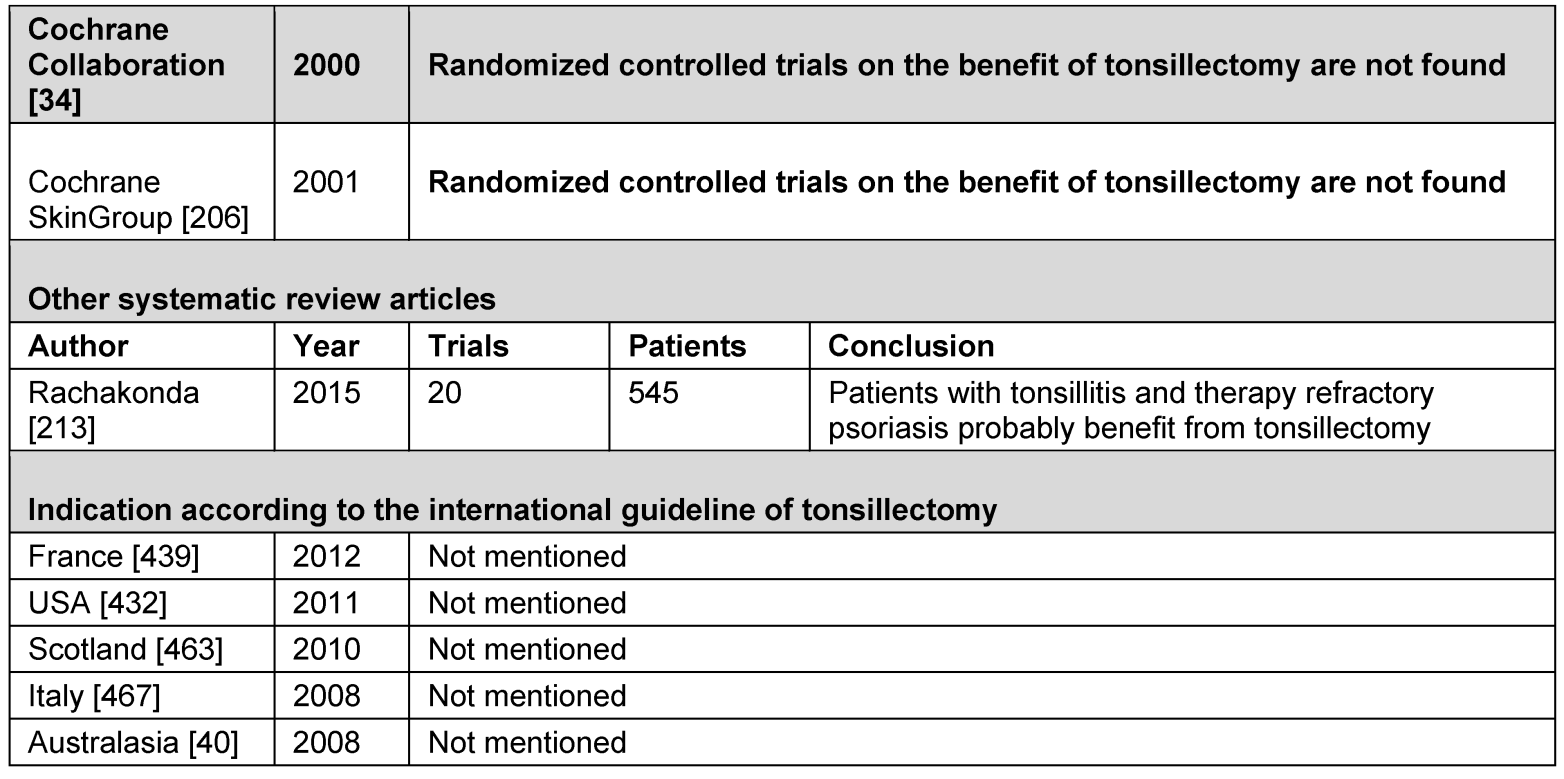
Trials and guidelines on the indication of psoriasis

**Table 6:**
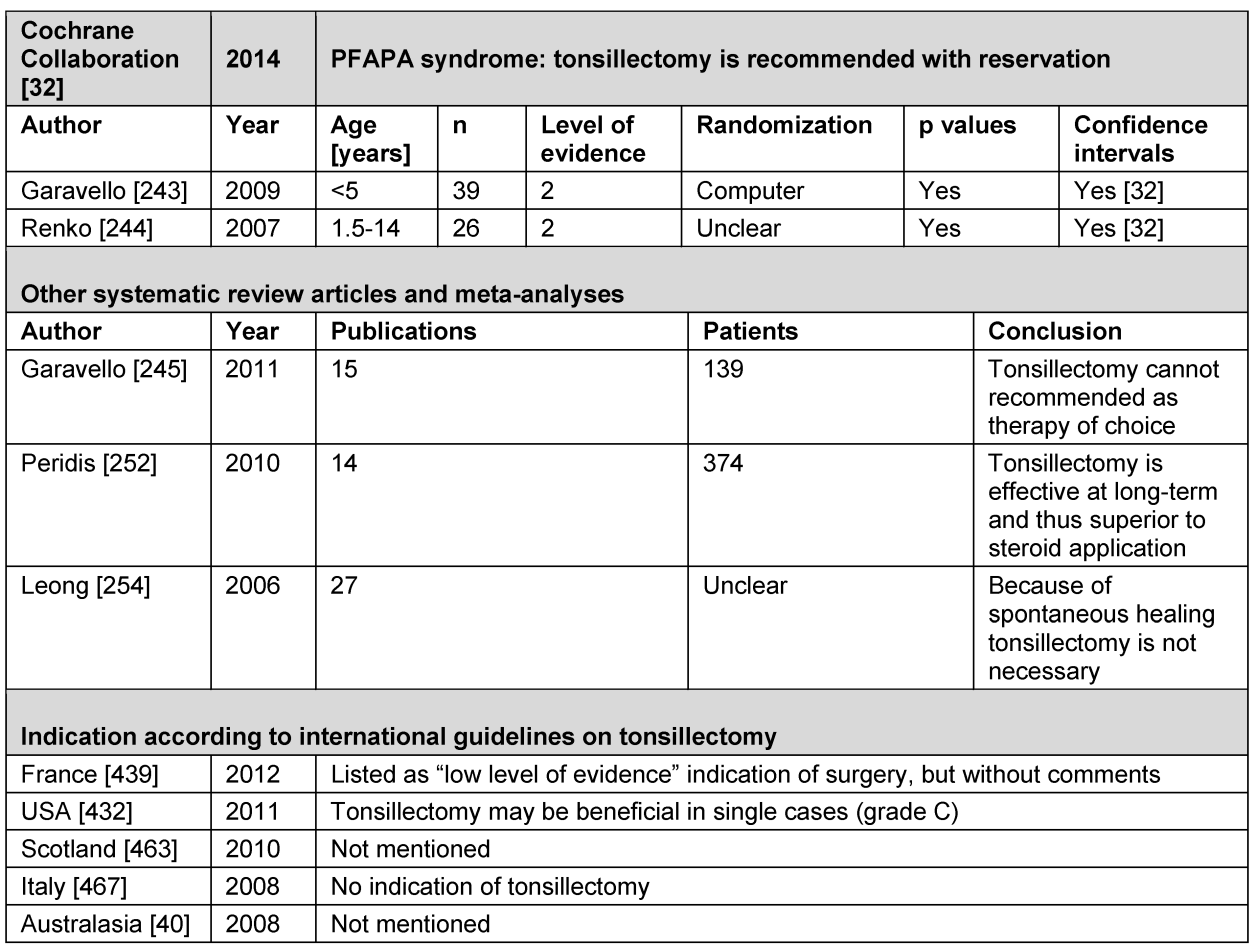
Studies and guidelines on the indication of PFAPA syndrome

**Table 7:**
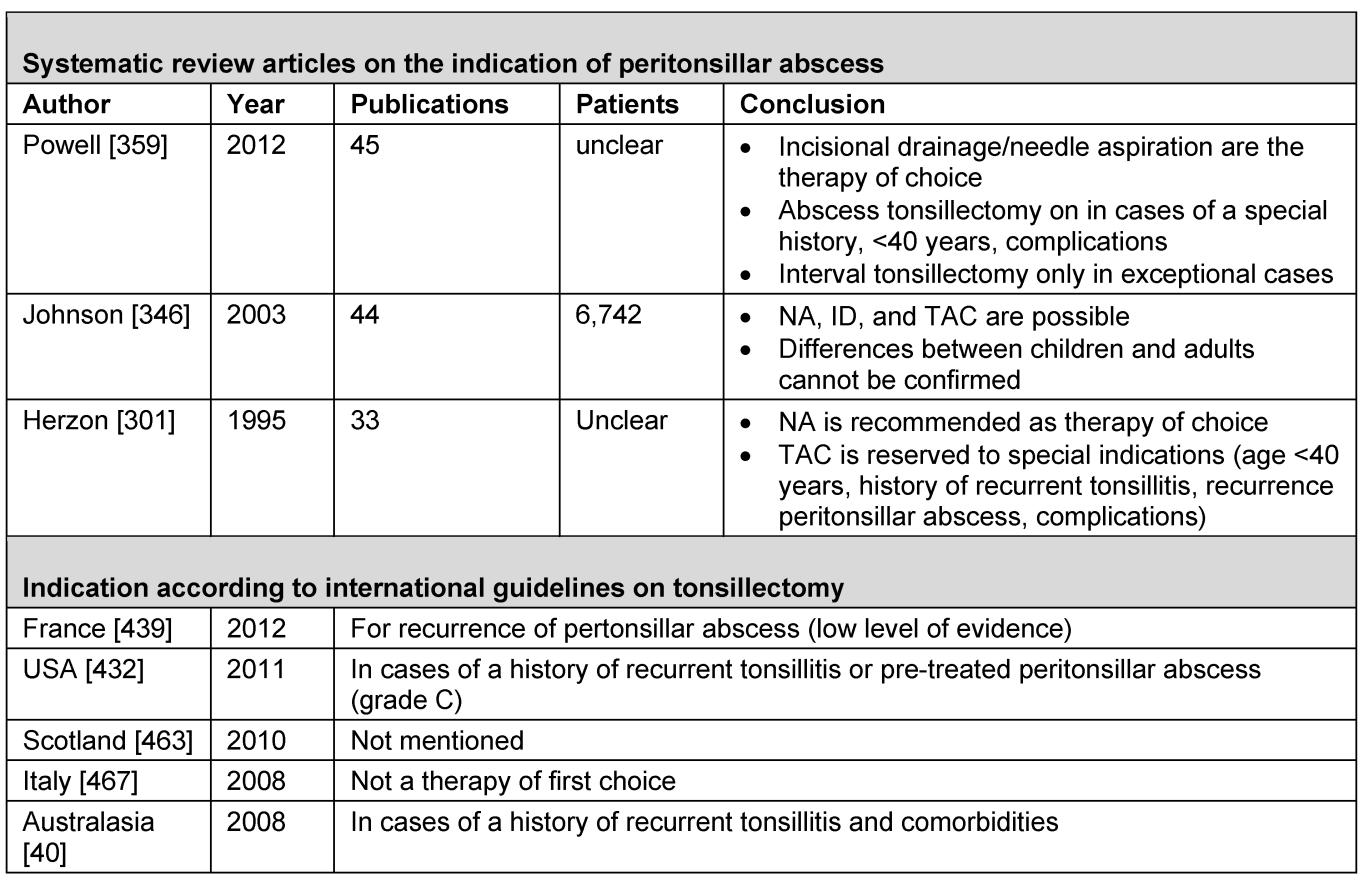
Trials and guidelines on the indication of peritonsillar abscess

**Table 8:**
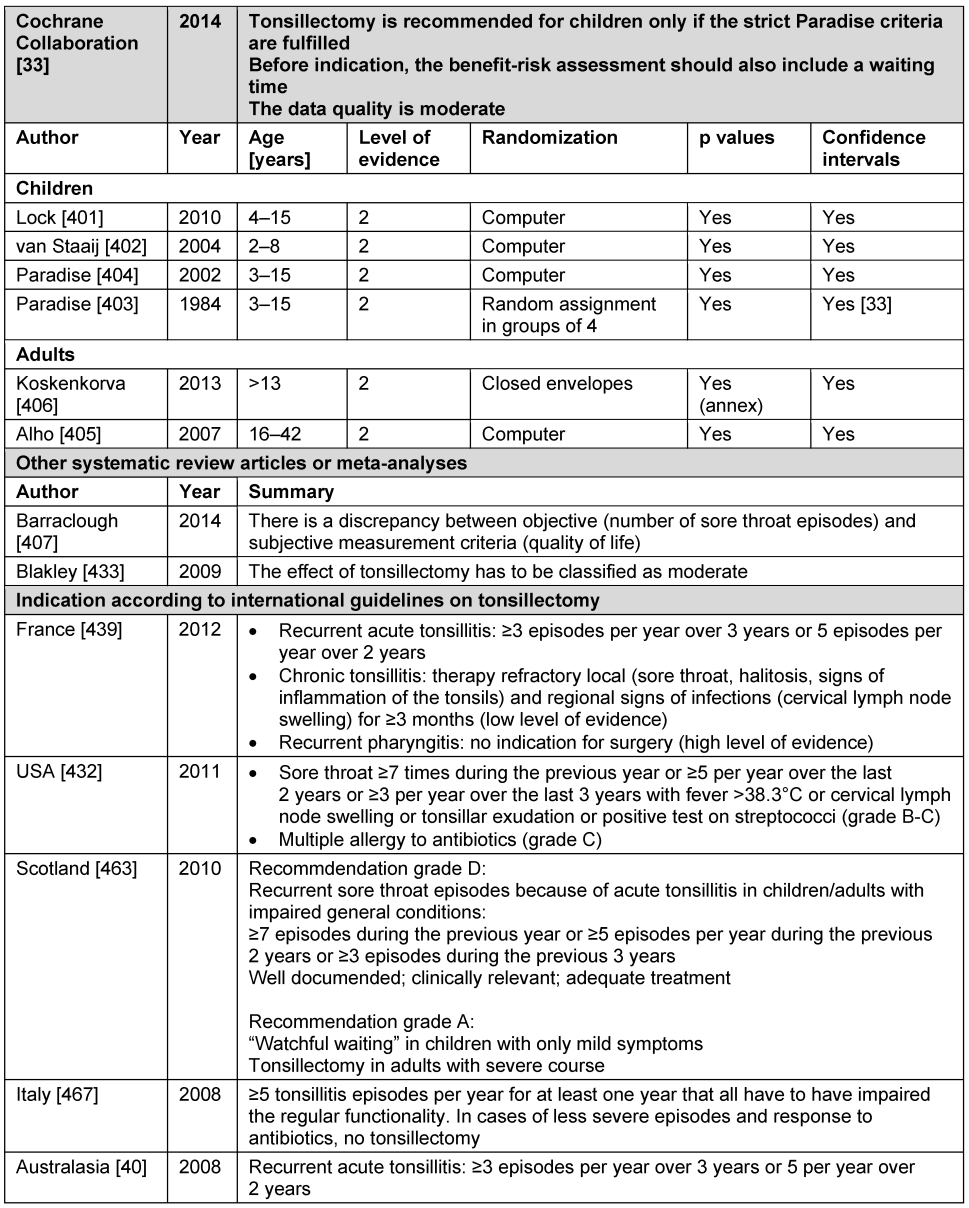
Trials and guidelines on the indication of tonsillitis

**Table 9:**
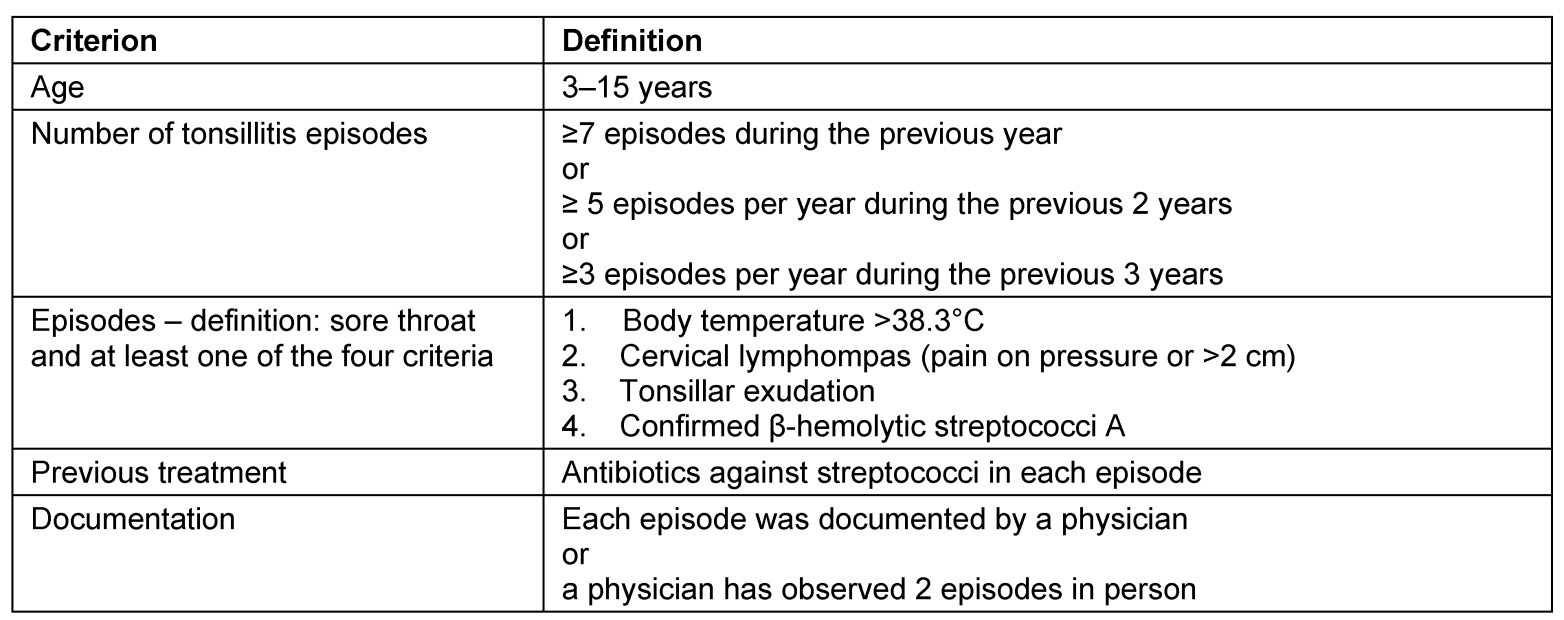
Indications for surgery according to Paradise et al. [403]

**Table 10:**
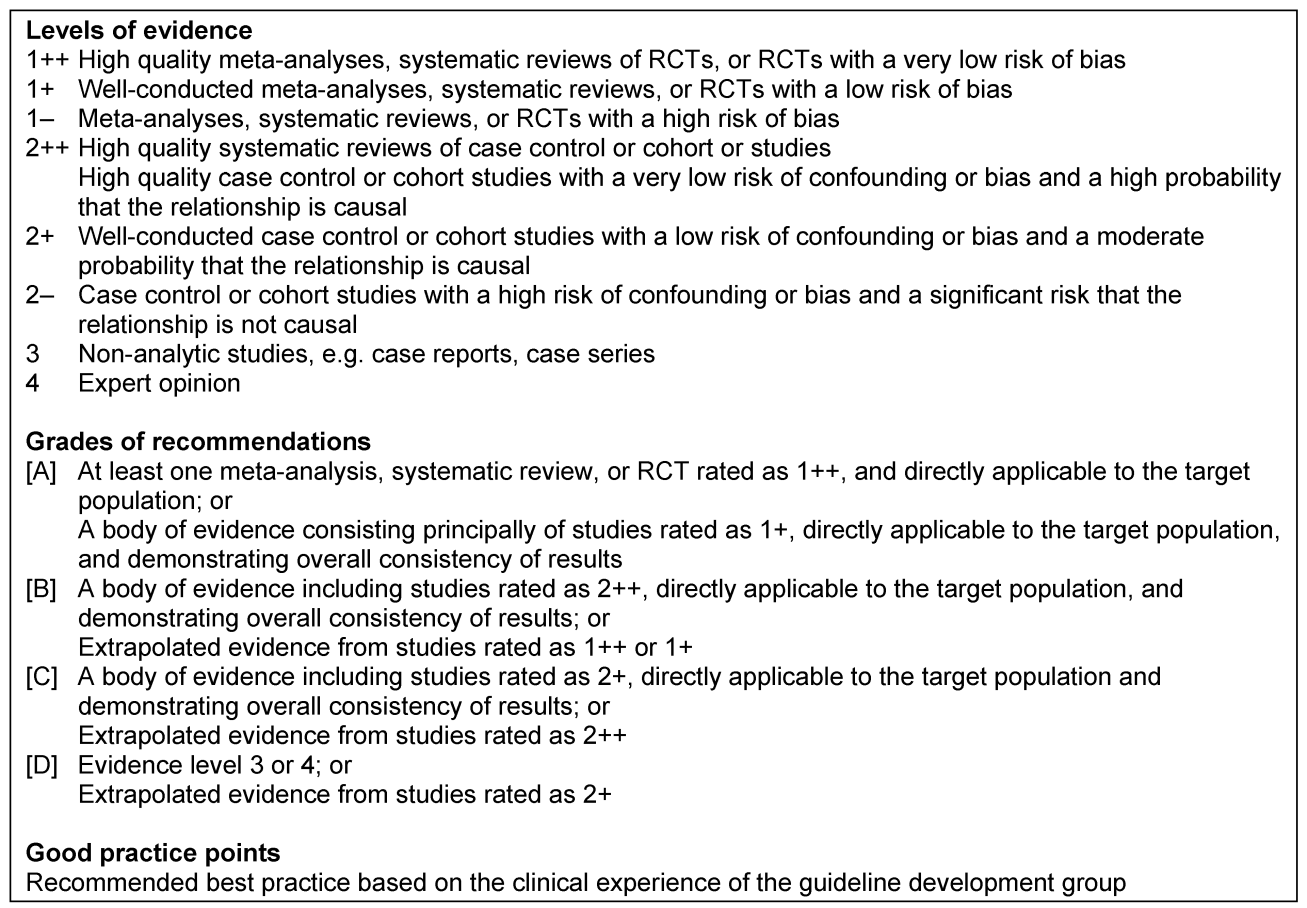
Scottish Intercollegiate Guidelines Network grading system 1999–2012 [360]

**Table 11:**
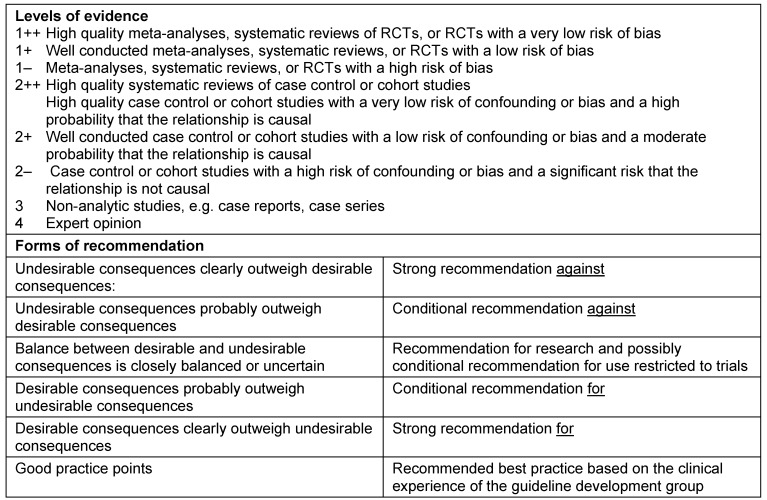
Scottish Intercollegiate Guidelines Network grading system 2014 [575]

**Table 12:**
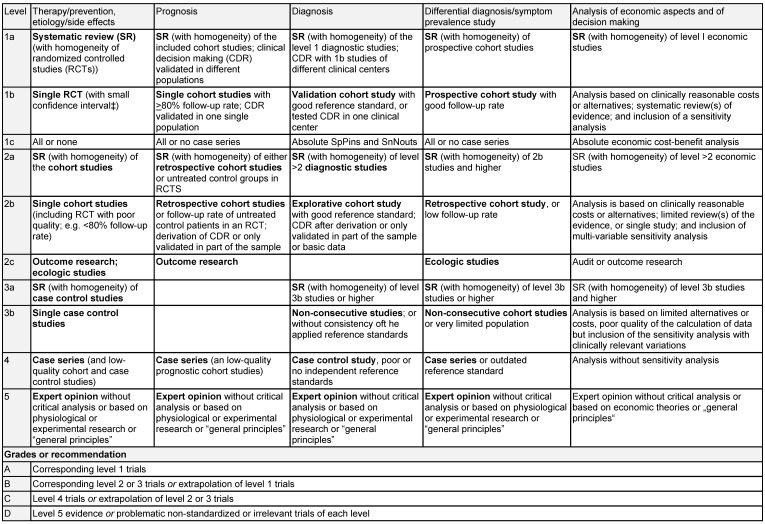
Oxford Centre for Evidence-based Medicine – levels of evidence (March 2009) [373] (Licensed under a Creative Commons Attribution 4.0 International License https://creativecommons.org/licenses/by/4.0/)
